# microRNA-7-5p inhibits melanoma cell proliferation and metastasis by suppressing RelA/NF-κB

**DOI:** 10.18632/oncotarget.9421

**Published:** 2016-05-17

**Authors:** Keith M. Giles, Rikki A.M Brown, Clarissa Ganda, Melissa J. Podgorny, Patrick A. Candy, Larissa C. Wintle, Kirsty L. Richardson, Felicity C. Kalinowski, Lisa M. Stuart, Michael R. Epis, Nikolas K. Haass, Meenhard Herlyn, Peter J. Leedman

**Affiliations:** ^1^ Laboratory for Cancer Medicine, Harry Perkins Institute of Medical Research and University of Western Australia Centre for Medical Research, Nedlands, WA, Australia; ^2^ Ronald O. Perelman Department of Dermatology, New York University School of Medicine, New York, NY, United States of America; ^3^ The University of Queensland Diamantina Institute, Translational Research Institute, Brisbane, Queensland, Australia; ^4^ Molecular and Cellular Oncogenesis Program, The Wistar Institute, Philadelphia, PA, United States of America; ^5^ School of Medicine and Pharmacology, The University of Western Australia, Nedlands, WA, Australia

**Keywords:** microRNA, miR-7-5p, RelA, melanoma, metastasis

## Abstract

microRNA-7-5p (miR-7-5p) is a tumor suppressor in multiple cancer types and inhibits growth and invasion by suppressing expression and activity of the epidermal growth factor receptor (EGFR) signaling pathway. While melanoma is not typically EGFR-driven, expression of miR-7-5p is reduced in metastatic tumors compared to primary melanoma. Here, we investigated the biological and clinical significance of miR-7-5p in melanoma. We found that augmenting miR-7-5p expression *in vitro* markedly reduced tumor cell viability, colony formation and induced cell cycle arrest. Furthermore, ectopic expression of miR-7-5p reduced migration and invasion of melanoma cells *in vitro* and reduced metastasis *in vivo*. We used cDNA microarray analysis to identify a subset of putative miR-7-5p target genes associated with melanoma and metastasis. Of these, we confirmed nuclear factor kappa B (NF-κB) subunit RelA, as a novel direct target of miR-7-5p in melanoma cells, such that miR-7-5p suppresses NF-κB activity to decrease expression of canonical NF-κB target genes, including IL-1β, IL-6 and IL-8. Importantly, the effects of miR-7-5p on melanoma cell growth, cell cycle, migration and invasion were recapitulated by RelA knockdown. Finally, analysis of gene array datasets from multiple melanoma patient cohorts revealed an association between elevated RelA expression and poor survival, further emphasizing the clinical significance of RelA and its downstream signaling effectors. Taken together, our data show that miR-7-5p is a potent inhibitor of melanoma growth and metastasis, in part through its inactivation of RelA/NF-κB signaling. Furthermore, miR-7-5p replacement therapy could have a role in the treatment of this disease.

## INTRODUCTION

Melanoma is the most aggressive form of skin cancer and its incidence continues to increase globally [1]. Through early detection, primary melanoma may be cured by surgery, however metastatic disease is typically refractory to chemotherapy and radiation therapy, and these patients generally have a poor prognosis. Increased understanding of the key molecular events that drive melanoma development and progression has led to the development of targeted therapies to treat metastatic melanoma, such as small molecule inhibitors that block the mutant BRAF V600 kinase found in 40-50% of tumors [2, 3]. However to date, the impact of targeted therapy in melanoma has been limited by the rapid and almost inevitable onset of therapeutic resistance *via* many different molecular mechanisms [4], the paucity of actionable mutations identified in NRAS mutant and BRAF/NRAS wild-type melanomas [5, 6], and the considerable inter- and intra-tumoral heterogeneity of the disease [7]. In parallel, advances in immunotherapy, particularly from understanding immune checkpoint pathways, have improved melanoma patient survival [8]. However, despite a number of durable clinical responses, many patients do not benefit from these therapies and toxicity is a significant concern, particularly with combined immune checkpoint blockade [8]. Thus, there remains an urgent need to develop novel approaches to treat melanoma, especially those able to target the inherent metastatic potential of the disease.

MicroRNAs (miRNAs) are a class of small non-coding RNAs that regulate gene expression at the post-transcriptional level by binding to the 3′-untranslated region (3′-UTR) of specific mRNAs, either targeting those transcripts for degradation and/or repressing their translation. Altered miRNA expression has been widely reported in cancer, and models in which high levels of oncogenic miRNAs downregulate tumor suppressor genes and under-expressed tumor suppressor miRNAs result in elevated oncogene expression have been proposed [9]. Aberrant miRNA expression has been linked to various stages of melanomagenesis, including transformation initiation, cell proliferation, apoptosis resistance, metastasis, and therapy resistance [10–13]. In addition to their prognostic or predictive utility as biomarkers, miRNAs have emerged as novel molecules for cancer treatment [14–16], where modulating expression and activity of a single cancer-associated miRNA has the potential to coordinately alter the function of an entire biological pathway within a given tumor type. miRNA replacement therapy is emerging as a potential therapeutic strategy in several cancers, and is being actively evaluated in clinical trials (eg. NCT01829971, clinicaltrials.gov). This emphasizes the emerging potential of using miRNAs to treat patients with metastatic melanoma by coordinate inhibition of key signaling pathways.

microRNA-7-5p (miR-7-5p) has diverse roles in development and disease and its expression is often aberrant in the latter [17]. In humans, miR-7 is transcribed from three genes, *miR-7-1*, *miR-7-2* and *miR-7-3*, and these transcripts are processed to yield the same mature miRNA sequence, of which the 5 strand is the most commonly studied. In the context of cancer, microRNA-7-5p (miR-7-5p) is a well characterized tumor suppressor that is frequently downregulated in a variety of cancers [18–21]. miR-7-5p has been reported to decrease tumor cell proliferation, anchorage-independent growth, migration and invasion, and to promote apoptosis and chemosensitivity by repressing expression of specific oncogenic target genes [22–28], suggesting that it has potential utility in the treatment of certain cancers. We previously characterized the role of miR-7-5p as a tumor suppressor and inhibitor of epidermal growth factor receptor (EGFR) expression and signaling in lung, glioblastoma, breast and head and neck cancers [27, 29]. miR-7-5p has been reported previously to be downregulated in several cases of metastatic *versus* primary melanoma, consistent with a tumor suppressor role, but, crucially, no studies have systematically investigated the role of miR-7-5p in metastatic melanoma development or its clinical significance [30–32]. We reported previously that miR-7-5p overexpression significantly inhibits the migration and invasion of melanoma cells *in vitro*, in part through regulation of insulin receptor substrate 2 (IRS-2) expression [30], but there are no reports investigating these effects and their mechanisms *in vivo*, or addressing the role of miR-7-5p in regulating melanoma cell growth.

Here, we demonstrate that miR-7-5p is a potent suppressor of melanoma cell growth and viability, and inhibits metastasis of melanoma cells to the lung. We also show that the anti-metastatic function of miR-7-5p is mediated, in part, *via* its direct suppression of RelA expression and NF-κB signaling. Importantly, high RelA expression is associated with a worse clinical outcome in melanoma patients.

## RESULTS

### miR-7-5p inhibits melanoma cell proliferation

**Figure 1:**
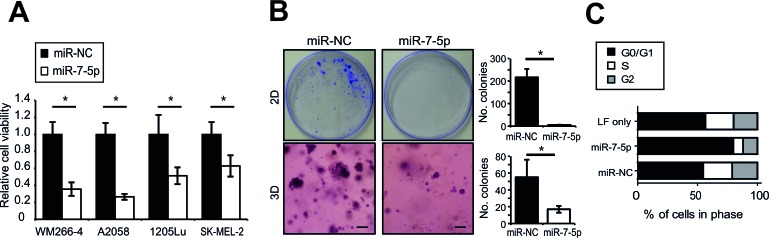
miR-7-5p inhibits melanoma cell proliferation *in vitro* **A.** Cell viability (MTS) assay of WM266-4, A2058, 1205Lu and SK-MEL-2 cells transfected with miR-7-5p or miR-NC precursor molecules for 5 d at 5 nM. *, *p*-value < 0.01. **B.** Representative images of 2D (plastic) and 3D (Matrigel) colony formation assay of WM266-4 cells transfected with miR-7-5p or miR-NC precursor molecules for 14 d period (30 nM) and stained with crystal violet. Scale bar = 200 μm. Colonies were counted manually with ImageJ software. *, *p*-value < 0.001. **C.** Flow cytometric cell cycle analysis of WM266-4 cells transfected with miR-7-5p or miR-NC precursor molecules for 3 d at 30 nM.

To investigate the functional role of miR-7-5p in melanoma we first assessed its impact on proliferation *in vitro*. Our results show a significant reduction in cell viability 5 days after transfection of the metastatic melanoma cell lines WM266-4, A2058, 1205Lu (all BRAF V600-mutant) and SK-MEL-2 (BRAF WT, NRAS^Q61K^) with miR-7-5p (Figure 1A). In parallel, we transfected these cells with an antagomiR to inhibit miR-7-5p function (anti-miR-7-5p), and observed no significant change in cell viability (Figure S1A), most likely due to the very low endogenous levels of miR-7-5p in melanoma cells. To further investigate the effect of ectopic miR-7-5p expression on melanoma cell proliferation, we assessed 2D and 3D colony formation, and observed fewer and smaller colonies in miR-7-5p-overexpressing WM266-4 cells in both assays (Figure 1B). Similar results were obtained with 1205Lu cells (Figure S2A). To investigate the mechanism underlying these effects, we assessed cell cycle distribution following propidium iodide (PI) staining and flow cytometry analysis. These experiments revealed that miR-7-5p induces G0/G1 cell cycle arrest in WM266-4 and 1205Lu cells (Figure 1C and Figure S2B). However, the reduction in cell number following miR-7-5p transfection does not appear to be due to apoptosis, evidenced by the lack of pre-G1 peak in cell cycle analysis, absence of PARP cleavage or caspase 3 and 7 activation, and lack of an apoptotic fraction following annexin V and PI staining and flow cytometry (Figure S3A-C). Together, these results indicate that miR-7-5p inhibits melanoma cell proliferation, but does not induce apoptosis.

### miR-7-5p inhibits melanoma cell migration and invasion *in vitro* and metastasis *in vivo*

**Figure 2:**
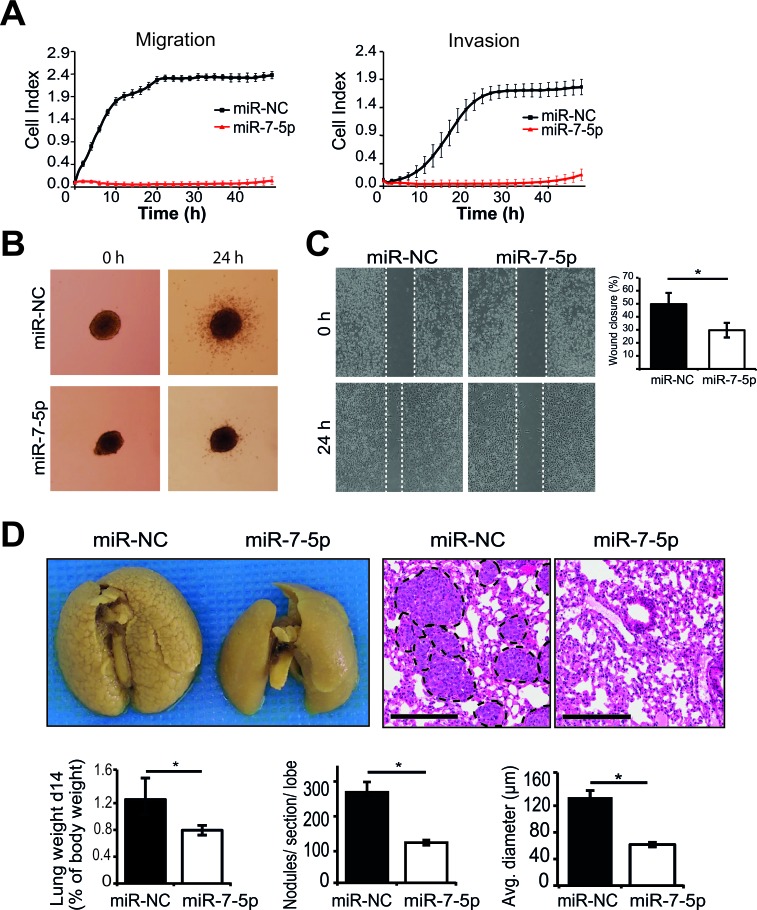
miR-7-5p inhibits migration and invasion *in vitro* and melanoma metastasis to the lung *in vivo* **A.** xCELLigence real-time assays of migration and invasion of 1205Lu cells following transfection with miR-7-5p or miR-NC for 48 h at 30 nM. **B.** 3D melanoma spheroid assay of 1205Lu cells transfected with miR-7-5p or miR-NC precursor molecules (30 nM), cultured to form spheroids and embedded in a matrix of collagen I. Invasion was monitored for 24 h and representative images are shown. **C.** Wound healing assay of 1205Lu cells transfected with either miR-7-5p or miR-NC precursor molecules (30 nM) for 48 h. Percentage wound closure was calculated using edge detection and area calculation with ImageJ software. *, *p*-value < 0.01. **D.** Microscopic and macroscopic examination of lung metastases in NSG mice 14 d post tail vein injection of 1205Lu cells transfected with miR-7-5p or miR-NC precursor molecules (30 nM for 48 h prior to injection, 9 mice/group). Summaries of the weight of lungs normalized to body weight, average number of metastases formed per lung section per lobe, and average tumor diameter are presented (bars represent mean ± SE) along with representative H&E stained sections and photographs of lungs harboring metastases. Scale bar = 300 μm. *, *p*-value < 0.01.

To further elucidate the functional role of miR-7-5p in melanoma, we assessed the impact of its overexpression on metastatic processes. We found that miR-7-5p abrogated migration and invasion of 1205Lu cells *in vitro* using real-time monitoring by xCELLigence (Figure 2A). In parallel, we measured cell proliferation in real-time and observed no difference within the first 24 h, therefore the early repression of cell migration and invasion cannot be due to accounted for by cell death or altered proliferation in this time period (Figure S4A). Next, we utilized a 3D spheroid invasion assay [33] as a more representative model of *in vivo* invasion, and found that miR-7-5p overexpression markedly inhibited the ability of 1205Lu cells to invade into the surrounding collagen (Figure 2B). Similar results were observed in WM266-4 cells (Figure S4B). Additionally, fewer cells migrated across a scratched wound following miR-7-5p augmentation, as assessed by wound healing assay of 1205Lu cells (Figure 2C). However, transfection of 1205Lu with anti-miR-7-5p to inhibit miR-7-5p function resulted in no significant difference in wound closure (Figure S4C), which is most likely due to the very low endogenous miR-7-5p expression in melanoma cells. To assess the impact of miR-7-5p on melanoma metastasis *in vivo*, 1205Lu cells with transient overexpression of miR-7-5p or miR-NC were injected into the tail vein of NSG mice and the lungs examined for metastatic deposits after 14 days. We found that the lungs from mice injected with 1205Lu cells that overexpressed miR-7-5p were significantly lighter and had markedly fewer tumors visible on the surface of the lungs (Figure 2D). Histological examination confirmed the significant reduction in lung tumor burden, with both fewer and smaller 1205Lu metastatic nodules being present in lungs from the miR-7-5p group (Figure 2D). 1205Lu cells formed tumors exclusively in the lungs of mice, as no nodules were found in the liver, heart or brain (data not shown), consistent with previous studies [34]. Collectively, these *in vitro* and *in vivo* data demonstrate the capacity for miR-7-5p to potently suppress melanoma metastasis *in vitro* and *in vivo*, and provide a foundation for subsequent studies to define the mechanisms responsible for these effects.

### miR-7-5p represses a pro-metastatic gene expression program in melanoma

**Figure 3:**
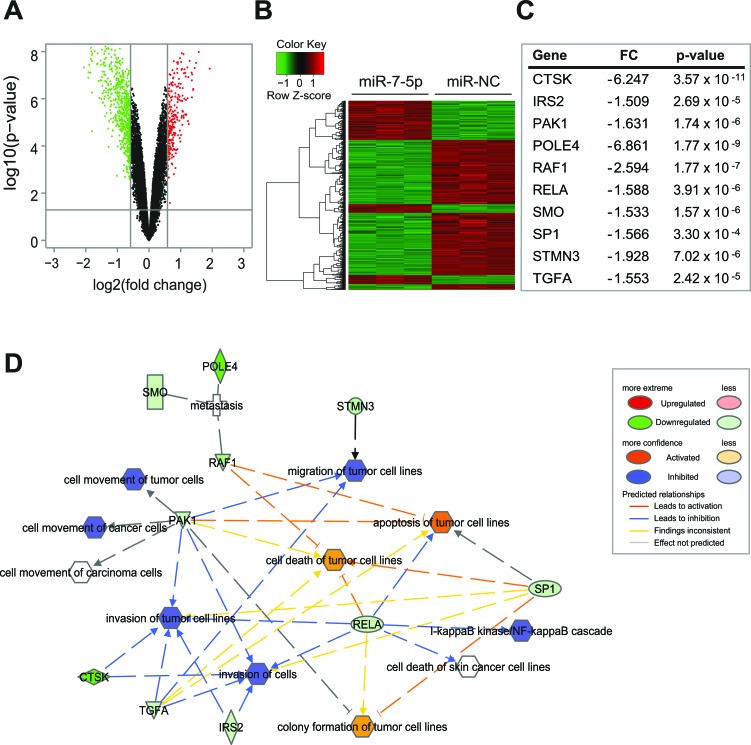
miR-7-5p downregulates a program of gene expression associated with melanoma, growth and metastasis **A.** Volcano plot representing fold change and significance of altered microarray probes in WM266-4 cells 24 h post-transfection with miR-7-5p or miR-NC precursor molecules (30 nM). **B.** Cluster analysis of genes downregulated (green) or upregulated (red) following miR-7-5p transfection. **C.** Signature of putative miR-7-5p target genes that contain a miR-7-5p seed sequence, are downregulated in the microarray and are associated with melanoma and metastasis. FC = Fold change. **D.** IPA network analysis showing growth- and metastasis-associated functions of putative miR-7-5p target genes in melanoma.

To better understand the mechanisms through which miR-7-5p regulates melanoma growth and metastasis, we performed gene expression array analysis using RNA isolated from WM266-4 cells that were transfected with miR-7-5p or miR-NC for 24 h. After data normalization, differentially expressed genes were assigned based on a cut-off of a fold-change of at least 1.5 and *p*-value < 0.05 (Figure 3A). Cluster analysis identified a total of 817 genes that were significantly upregulated or downregulated by miR-7-5p transfection, relative to miR-NC (Figure 3B and Supplementary Tables S1 and S2).

To identify direct putative targets of miR-7-5p in melanoma that might account for its anti-proliferative and anti-metastatic effects, we focused on those genes that were significantly downregulated in WM266-4 cells following transfection of miR-7-5p, which refined the list to 575 genes, of which 129 were identified by Ingenuity Pathway Analysis (IPA) software as being predicted (by TargetScan) or proven miR-7-5p targets (Supplementary Table S3). Biofunctional analysis of this gene list with IPA showed enrichment for genes involved in cell movement, cell cycle, colony formation, proliferation and cancer (Supplementary Table S4). Further analysis of this putative target list identified 18 genes that are associated with growth and/or metastatic processes (Supplementary Table S5), and of these 10 (CTSK, IRS2, PAK1, POLE4, RAF1, RELA, SMO, SP1, STMN3, TGFA) have been previously implicated in melanoma, thus representing a pro-metastatic gene signature that is repressed by miR-7-5p (Figure 3C). RT-qPCR analysis confirmed significant downregulation of mRNA expression of each of these genes by miR-7-5p in WM266-4, 1205Lu and A2058 cells (Figure S5). Additional bioinformatics analyses using this 10-gene signature identified an integrated network of interactions between the miR-7-5p-downregulated genes related to metastasis and invasion in melanoma (Figure 3D), that potentially confer the *in vitro* and *in vivo* anti-metastatic and anti-proliferative effects.

### Impact of pro-metastatic gene signature on melanoma patient survival

**Figure 4:**
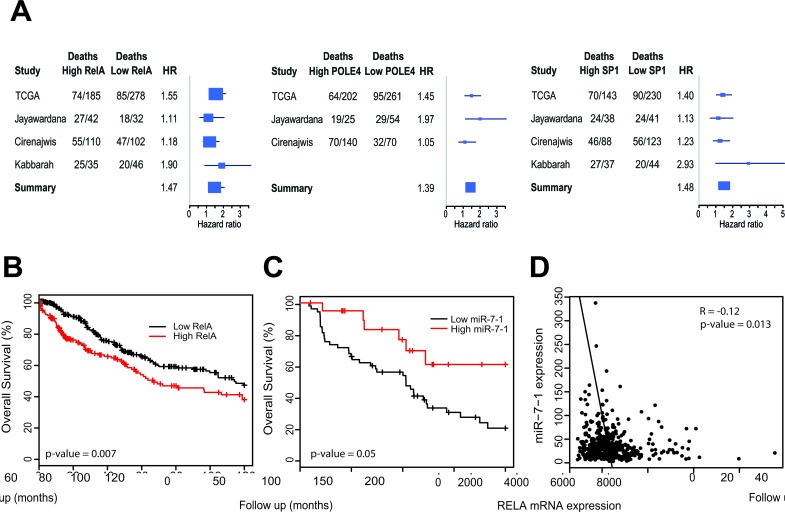
Prognostic significance of RelA, POLE4, SP1 and miR-7-1 in melanoma **A.** Forest plots summarizing risk of death associated with high or low expression of RelA, POLE4 and SP1, across four melanoma patient cohorts (TCGA, Jayawardana, Cirenajwis and Kabbarah). HR = Hazard ratio. **B.** Kaplan-Meier curve representing overall survival for melanoma patients with tumors expression low or high tumor RelA mRNA, after adjustment for age and sex over 10-year follow-up period from TCGA, *n* = 463. **C.** Kaplan-Meier curve representing overall survival for melanoma patients with tumors expression low or high miR-7-1 levels, after adjustment for age and sex over 200-month follow-up from Jayawardana, *n* = 74. **D.** Negative correlation between expression of RelA and miR-7-1 in TCGA melanoma data, *n* = 463.

To assess the clinical significance of increased expression of the above miR-7-5p target genes in melanoma, we analyzed publically available microarray data from The Cancer Genome Atlas melanoma study (TCGA; http://cancergenome.nih.gov). Data on survival and mRNA expression for 463 melanoma patients was analyzed by Cox regression, and after adjustment for age and sex, high expression of RelA (hazard ratio (HR) = 1.55, *p*-value = 0.007; Figure 4B), POLE4 (HR = 1.45, *p*-value = 0.023) and SP1 (HR = 1.40, *p*-value = 0.032) was predictive of poorer overall survival (Table 1). To validate this finding, we performed meta-analyses of these genes across three additional melanoma cohorts [35–37], in addition to the TCGA study, to increase the sample size and statistical power. Comparisons across these four datasets showed high expression of either RelA (consensus HR = 1.47), POLE4 (consensus HR = 1.39) or SP1 (consensus HR = 1.48) was consistently associated with an increased risk of death in melanoma (Figure 4A and 4B). Furthermore, low expression of miR-7-1, which is the predominant isoform that gives rise to mature miR-7-5p, was associated with an increased risk of death in melanoma patients, with HR = 0.558, *p* = 0.05 (Figure 4C) [37]. Additionally, TCGA melanoma data was analyzed for an inverse correlation between expression of miR-7 isoforms and putative target gene expression, and revealed a negative association between the miR-7-1 isoform and RelA with *R* = −0.12, *p* = 0.013, but no significant negative correlations with the other genes that were also associated with survival (Figure 4D and Supplementary Table S6). Taken together, these data suggest that RelA overexpression and low miR-7-1 levels are indicators of a poor prognosis in melanoma. In addition, the data identified a negative correlation between levels of miR-7-5p and RelA in these tumors, supporting a model whereby miR-7-5p loss may contribute to increased expression of RelA.

**Table 1:** Prognostic significance of putative miR-7-5p target gene expression on melanoma patient survival in TCGA melanoma cohort

Gene Symbol	HR	*p*-value	95% CI
RELA	1.53	0.010	1.11-2.10
IRS2	0.93	0.641	0.68-1.27
POLE4	1.45	0.023	1.05-2.00
PAK1	0.80	0.167	0.59-1.10
STMN3	1.13	0.451	0.83-1.54
SP1	1.40	0.035	1.02-1.92
TGFA	0.90	0.498	0.66-1.23
RAF1	1.17	0.337	0.85-1.60
CTSK	1.04	0.791	0.76-1.43
SMO	1.17	0.319	0.86-1.60

Cox regression analysis, adjusted for age and gender Deaths=160, *n* = 463

Abbreviations: HR, Hazard ratio; CI, Confidence interval of HR.

### miR-7-5p reduces RelA expression by directly targeting its 3′-UTR

**Figure 5:**
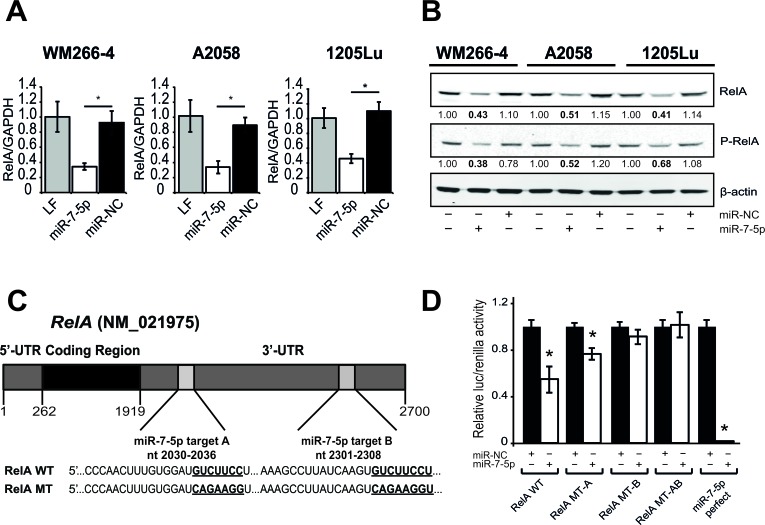
miR-7-5p downregulates RelA expression *via* specific binding to two target sites within the RelA 3′-UTR **A.** RT-qPCR analysis of RelA expression 24 h after transfection of miR-NC or miR-7-5p precursor molecules in WM266-4, A2058 and 1205Lu melanoma cells. *, *p*-value < 0.01. **B.** Western blotting analysis of RelA and phospho-RelA protein levels 3 d after transfection of miR-NC or miR-7-5p precursor molecules. β-actin, loading control. **C.** Schematic representation of the *RelA* mRNA with two 3′-UTR miR-7-5p binding sites (A and B) predicted by TargetScan and sequences of wildtype (RelA WT) and mutant (RelA MT) miR-7-5p target sites. Seed regions are in bold and underlined. **D.** Luciferase reporter assay with 1205Lu cells that were transfected with wildtype (RelA WT) or mutant (RelA MT-A, MT-B, or MT-AB) 3′-UTR, or miR-7-5p perfect firefly-luciferase reporter plasmids, control *Renilla*-luciferase plasmid, and either miR-7-5p or miR-NC precursor molecules at 2.5 nM. *, *p*-value < 0.01.

We elected to focus on the NF-κB subunit RelA (p65) for further functional studies, given its prognostic significance in melanoma and its inverse correlation with miR-7-5p levels. To confirm the direct regulation of RelA expression by miR-7-5p in melanoma, we used quantitative real-time PCR (RT-qPCR) and western blotting and found that miR-7-5p reduces RelA mRNA (Figure 5A) and protein expression (Figure 5B) in WM266-4, A2058 and 1205Lu melanoma cells. TargetScan analysis identified two putative miR-7-5p binding sites within the RelA 3′-UTR at positions 2030-2036 nt (site A, poorly conserved) and at 2301-2308 nt (site B, highly conserved, Figure 5C). To test the direct binding of miR-7-5p to these sites, wild type or mutant full length RelA 3′-UTR luciferase reporter constructs were generated that included either: (i) wild type miR-7-5p sites (RelA WT) (ii) mutant miR-7-5p site A (RelA MT-A), (iii) mutant miR-7-5p site B (RelA MT-B), or (iv) both mutant miR-7-5p sites (RelA MT-AB). Mutant miR-7-5p sites were engineered to completely disrupt binding within seed regions. In 1205Lu cells, miR-7-5p significantly reduced luciferase activity of the RelA WT 3′-UTR or miR-7-5p perfect target reporter (positive control), compared to miR-NC (Figure 5D). When the first, poorly conserved miR-7-5p site was mutated (RelA MT-A), miR-7-5p significantly reduced luciferase activity, but to a lesser extent than the RelA-WT reporter. However, mutation of the highly conserved site (RelA MT-B) or both sites (RelA MT-AB) eliminated miR-7-5p's ability to abrogate reporter activity. Similar results were obtained with WM266-4 and A2058 melanoma cells (Figure S6A). Together, these data suggest that in melanoma cells, miR-7-5p directly targets two sites within the 3′-UTR of RelA to inhibit RelA mRNA and protein expression.

### miR-7-5p inhibits NF-κB transcriptional activity and downstream signaling

**Figure 6:**
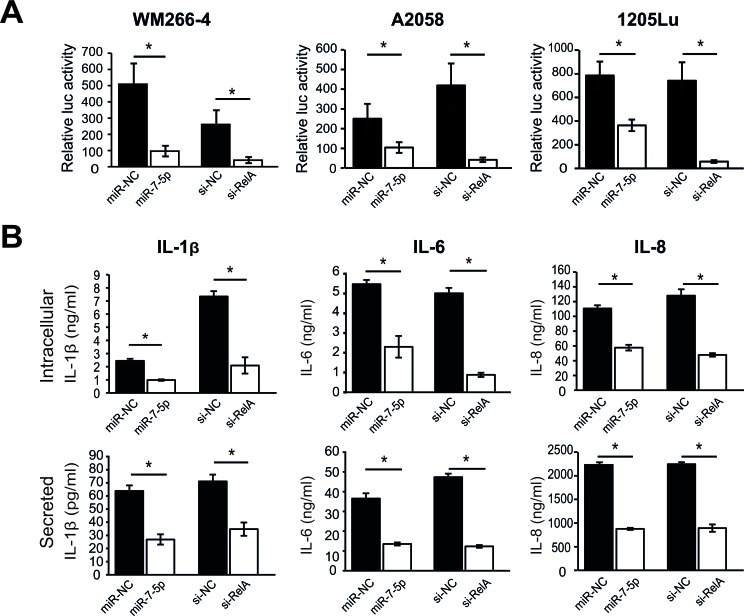
miR-7-5p downregulates NF-κB transcriptional activity and downstream NF-κB targets in melanoma cells **A.** Luciferase reporter assay with WM266-4, A2058 and 1205Lu cells that were transfected with NF-κB cignal luciferase reporter or negative control luciferase reporter, and either miR-7-5p or miR-NC precursor molecules (10 nM), RelA siRNA (si-RelA7, positive control for assay) or negative control siRNA (si-NC) at 5 nM. *, *p*-value < 0.01. **B.** ELISA analysis of secreted and intracellular expression of IL-1β, IL-6 and IL-8 in 1205Lu cells 3 d post-transfection with either miR-7-5p or miR-NC precursor molecules, or si-RelA7 or si-NC. *, *p*-value < 0.01.

As RelA is a NF-κB subunit responsible for activating the transcription of many genes, we assessed the impact of miR-7-5p on NF-κB transcriptional activity in melanoma cells. Cells were co-transfected with a luciferase reporter construct containing multiple canonical NF-κB transcriptional regulatory elements (TREs) or a negative control luciferase plasmid lacking TREs (for background measurement), plus either miR-7-5p, miR-NC, RelA siRNA (si-RelA, positive control) or non-targeting siRNA (si-NC). Cells transfected with miR-7-5p or si-RelA had significantly reduced NF-κB reporter activity, compared to their respective controls (Figure 6A). In view of the reduced NF-κB transcriptional activity in melanoma cells, we next hypothesized that expression of key NF-κB target genes would also be reduced. To test this, we screened for miR-7-5p-regulated genes using a NF-κB pathway PCR array with WM266-4 cells transfected with miR-NC or miR-7-5p for 3 d. Multiple NF-κB target genes were found to be deregulated in response to miR-7-5p overexpression, and these changes were subsequently verified by RT-qPCR in WM266-4 and 1205Lu cells (Supplementary Table S7). We further investigated IL-1β, a cytokine associated with melanoma metastasis [38] as a miR-7-5p-regulated NF-κB target gene. Transfection of 1205Lu cells with either miR-7-5p or si-RelA resulted in reduced secretion and intracellular expression of IL-1β protein (Figure 6B), as measured by ELISA. As IL-1β has also been linked to the expression of other cancer-associated cytokines [38], we tested the impact of miR-7-5p or si-RelA on expression of IL-6 and IL-8, and found that both miR-7-5p and si-RelA reduced the levels of secreted and intracellular IL-6 and IL-8 (Figure 6B and Supplementary Table S7). These data indicate that the direct targeting of RelA by miR-7-5p reduces NF-κB activity and consequently expression of many of the NF-κB downstream target genes associated with tumor metastasis.

### RelA is a functional target of miR-7-5p

**Figure 7:**
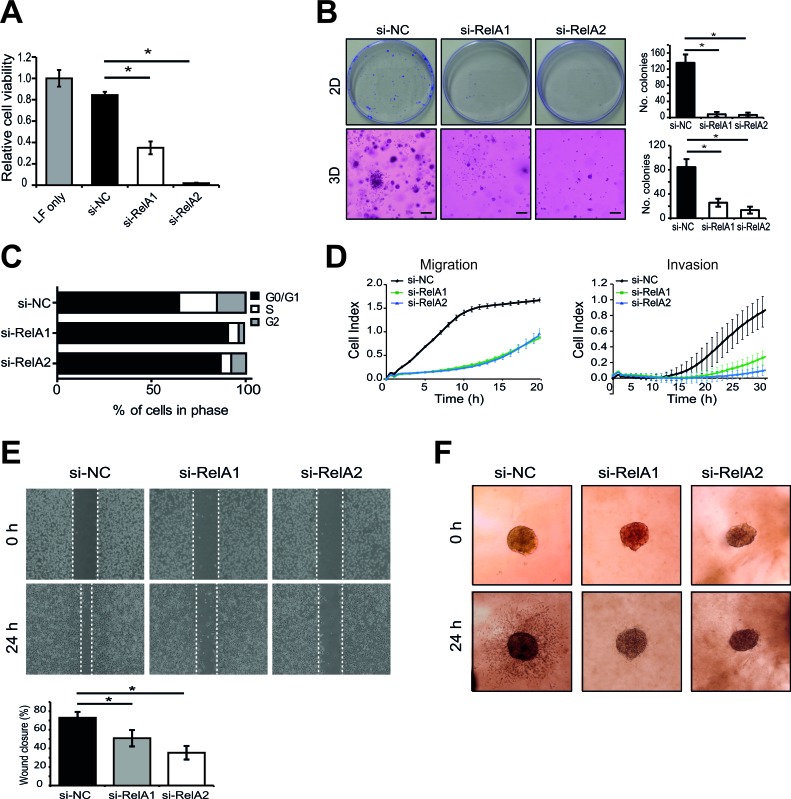
RelA knockdown recapitulates the effects of miR-7-5p on melanoma cell viability, cell cycle, migration and invasion **A.** Cell viability assay (MTS) of WM266-4 cells transfected with RelA siRNAs (si-RelA1 or 2) or si-NC for 5 d at 5 nM. **B.** Representative images of 2D (plastic) and 3D (matrigel) colony formation assay of WM266-4 cells transfected with RelA siRNAs (si-RelA1 or 2) or si-NC (5 nM) for 14 d period and stained with crystal violet and number of colonies shown graphically. *, *p*-value < 0.001. **C.** Flow cytometric cell cycle analysis of WM266-4 cells transfected with RelA siRNAs (si-RelA1 or 2) or si-NC (5 nM) for 3 d. **D.**. xCELLigence real-time assays of migration and invasion following transfection of RelA siRNAs (si-RelA1 or 2) or si-NC (5 nM) for 48 h. **E.** Wound healing assay of 1205Lu cells transfected with either RelA siRNAs (si-RelA1 or 2) or si-NC (5 nM) for 48 h prior to addition to 35 mm μ-dishes. Bar graph showing percentage differences in wound closure after 24 h. *, *p*-value < 0.01. **F.** 3D melanoma spheroid assay of 1205Lu cells transfected with RelA siRNAs (si-RelA1 or 2) or si-NC (5 nM), cultured to form spheroids and embedded in a matrix of collagen I. Invasion was monitored for 24 h and representative images are shown.

Next, we sought to establish a functional role for RelA as a miR-7-5p target in melanoma, such that repression of RelA and its downstream signaling might in part explain the capacity of miR-7-5p to inhibit proliferation, migration and invasion of melanoma cells. WM266-4 and 1205Lu cells were transfected with five different RelA siRNAs, each of which reduced RelA mRNA and protein expression by approximately 70-95% in both cell lines (Figure S6B-C). Silencing of RelA expression in WM266-4 cells significantly reduced their viability (Figure 7A), colony forming potential in both 2D and 3D models (Figure 7B), and also resulted in G0/G1 phase cell cycle arrest (Figure 7C). Similar results were obtained with 1205Lu cells (Figure S7A-C). Furthermore, transfection of 1205Lu cells with RelA siRNAs significantly reduced the rate of cell migration and invasion, impaired wound closure in a wound healing assay, and suppressed invasion of melanoma spheroids (Figure 7D-F). Thus, RelA knockdown was able to recapitulate the phenotypic effects of ectopic miR-7-5p overexpression in melanoma cells. Collectively, these data suggest that miR-7-5p is a potent inhibitor of melanoma growth, migration and invasion, at least in part through the targeted reduction of RelA expression/activity and its downstream targets.

## DISCUSSION

miR-7-5p suppresses the growth and metastasis of several tumor types, including hepatocellular carcinoma, breast cancer, glioma and gastric cancer, by inhibiting the expression of specific target genes, including EGFR and its signaling pathways [21, 39, 40]. Given that melanoma is typically not driven by aberrant EGFR expression and/or signaling, this study aimed to clarify the functional and clinical relevance of miR-7-5p in melanoma, and to identify critical pathways through which miR-7-5p acts in this disease. We found that overexpression of miR-7-5p not only suppresses melanoma cell growth and cell cycle progression, but also tumor cell migration, invasion and metastasis *in vivo*. Gene expression analyses revealed a signature of putative miR-7-5p target genes associated with melanoma and metastasis. Further, aberrant expression of some of these genes including RelA, POLE4 and SP1 had prognostic implications in terms of melanoma patient survival. We focused on RelA, due to its negative association with miR-7-5p expression and emerging evidence establishing it as a potential target for metastatic melanoma treatment [41–43]. Our study shows that RelA is a direct target of miR-7-5p, *via* two independent binding sites in its 3′-UTR, which reduces its expression, activity and downstream signaling, and our functional experiments validate RelA as a critical mediator of the anti-metastatic effects of miR-7-5p in melanoma.

Analysis of four independent melanoma clinical cohorts showed that RelA overexpression is associated with worse overall survival, supporting its tumor-promoting role in this system [44–46]. Further analyses of these data revealed an inverse correlation between miR-7-1 and RelA levels in melanoma and that miR-7-1 levels were associated with survival, consistent with a model in which reduced expression of miR-7-5p promotes RelA overexpression and facilitates melanoma progression. While this manuscript was in preparation, Hanniford and coworkers reported that reduced miR-7-5p expression was associated with melanoma recurrence and reduced invasion *in vitro* [47]. Another recent report suggested that serum miR-7-5p levels might be a useful biomarker for tumors of the esophagus [48]. Certainly, additional studies are required to more clearly define the prognostic significance of miR-7-5p expression in large cohorts of primary and metastatic melanoma.

Our expression array data indicate that miR-7-5p functions to regulate large networks of genes in melanoma, and our network analysis supports the concept of regulation *via* “hub genes”, which includes RelA, a transcriptional regulator that itself regulates expression of many other genes, and hence altering miR-7-5p expression produces a significant “flow on” effect in terms of broadly altering gene expression [62]. We found that downregulation of RelA by miR-7-5p resulted in reduced expression of various canonical targets of NF-κB, including IL-1β, thus providing additional evidence for the indirect inhibition of broad transcriptional networks. Many of these canonical NF-κB target genes, including IL-1β, IL-6 and IL-8, have been associated with melanoma tumorigenicity, metastasis and angiogenesis, and represent potential targets for therapy [38, 49, 50]. Importantly, using siRNAs against RelA, we were able to recapitulate the functional effects of miR-7-5p on melanoma cell proliferation, migration and invasion, thus validating RelA as a direct, functional target of miR-7-5p in melanoma.

Interesting links between miR-7-5p and the NF-κB pathway have recently emerged. A study by Ning et al. identified loss of expression of the transcription factor Hepatocyte Nuclear Factor 4α (HNF4α) as mediating reduced miR-7-5p levels in metastatic hepatocellular carcinoma, and showed that ectopic expression of HNF4α reduced NF-κB activity and inhibited tumor cell migration, invasion and lung metastasis [39]. It is unclear whether the reduced miR-7-5p levels observed in some melanomas can be explained by loss of HNF4α expression or activity. Recently, a report by Zhao et al. showed that miR-7-5p reduced gastric carcinoma cell proliferation, induced G0/G1 cell cycle arrest, promoted apoptosis, suppressed tumor growth *in vivo*, and suggested that RelA and FOS could mediate these effects [51]. Of particular interest, they demonstrated that RelA/NF-κB could repress miR-7-5p transcription. Thus, it seems possible that miR-7-5p can directly target RelA in melanoma, and that, in turn, NF-κB can act in a reciprocal feedback loop to downregulate expression of miR-7-5p [51].

Our study identified several other putative miR-7-5p target genes relevant to melanoma. Most notably, we report that high expression of specific protein 1 (SP1) and polymerase (DNA-directed), epsilon 4, accessory subunit (POLE4) was associated with significantly worse overall survival in the TCGA melanoma cohort. SP1 is a ubiquitous transcription factor that has been implicated in driving constitutive expression of cell adhesion molecules in metastatic melanoma [52], and Li et al. recently showed that miR-7a/b overexpression in rat cardiac fibroblasts (CFs) suppresses SP1 expression and CF cell growth and migration [53]. Interestingly, miR-22 has been shown to form an autoregulatory loop with SP1 in breast cancer, whereby miR-22 binds to the 3′-UTR of SP1 and represses its expression, SP1 binding to the miR-22 promoter suppresses miR-22 transcription, and miR-22 overexpression suppresses breast cancer cell growth, invasion, and metastasis [54]. In this regard, it would be interesting to examine whether SP1 binding to the miR-7-5p promoter can directly inhibit miR-7-5p expression, suggesting a similar feedback loop may also exist in melanoma. Intriguingly, there are SP1 binding sites in the RelA promoter and SP1 has been shown to interact with and form a complex with RelA *in vitro* [55, 56]. As miR-7-5p can co-ordinately downregulate RelA and SP1, these observations may explain the altered expression of many genes by miR-7-5p that do not contain classic miR-7-5p seed regions. Elevated levels of POLE4 and other DNA repair genes have been associated with shorter time to melanoma relapse [57], and in previous studies we identified POLE4 as a putative miR-7-5p target gene in lung and head and neck cancer cell lines [27, 29]. Together, our findings suggest that SP1 and POLE4 could represent additional functional targets of miR-7-5p in melanoma, emphasizing the coordinate manner in which miRNAs suppress expression of multiple target genes to exert their biological effects.

Therapy for metastatic melanoma has undergone dramatic change in the recent years with the development of molecularly-targeted therapies and immune checkpoint inhibitors. miR-7-5p is known to target several of the molecules, such as IGF1R, EGFR and IRS1/2, that promote resistance to BRAF inhibitors [58–60]. Thus, patients who have developed resistance to BRAF inhibitor therapy could potentially benefit from miR-7-5p replacement therapy, which might resensitize tumors to these treatments. Further, we and others have shown that miR-7-5p is capable of sensitizing other resistant tumor types to targeted therapies, such as the EGFR tyrosine kinase inhibitor erlotinib [61]. In support of this concept, Stark et al. have shown that miR-514a modulates BRAF inhibitor sensitivity in melanoma through its regulation of the tumor suppressor NF1 [62]. In addition, miR-7-5p could potentially augment the anti-melanoma immune response, by blocking expression of immunosuppressive cytokines, such as IL-6 [63]. Ultimately, this could lead to studies investigating the efficacy of combining miR-7-5p with immune checkpoint blockade therapy. As treatment of metastatic melanoma increasingly involves combination therapies, it will be of great interest to define which combinations of miR-7-5p and targeted therapies (BRAF/MEK inhibitors) or immunotherapies are most effective or yield synergistic anti-melanoma effects.

**Figure 8:**
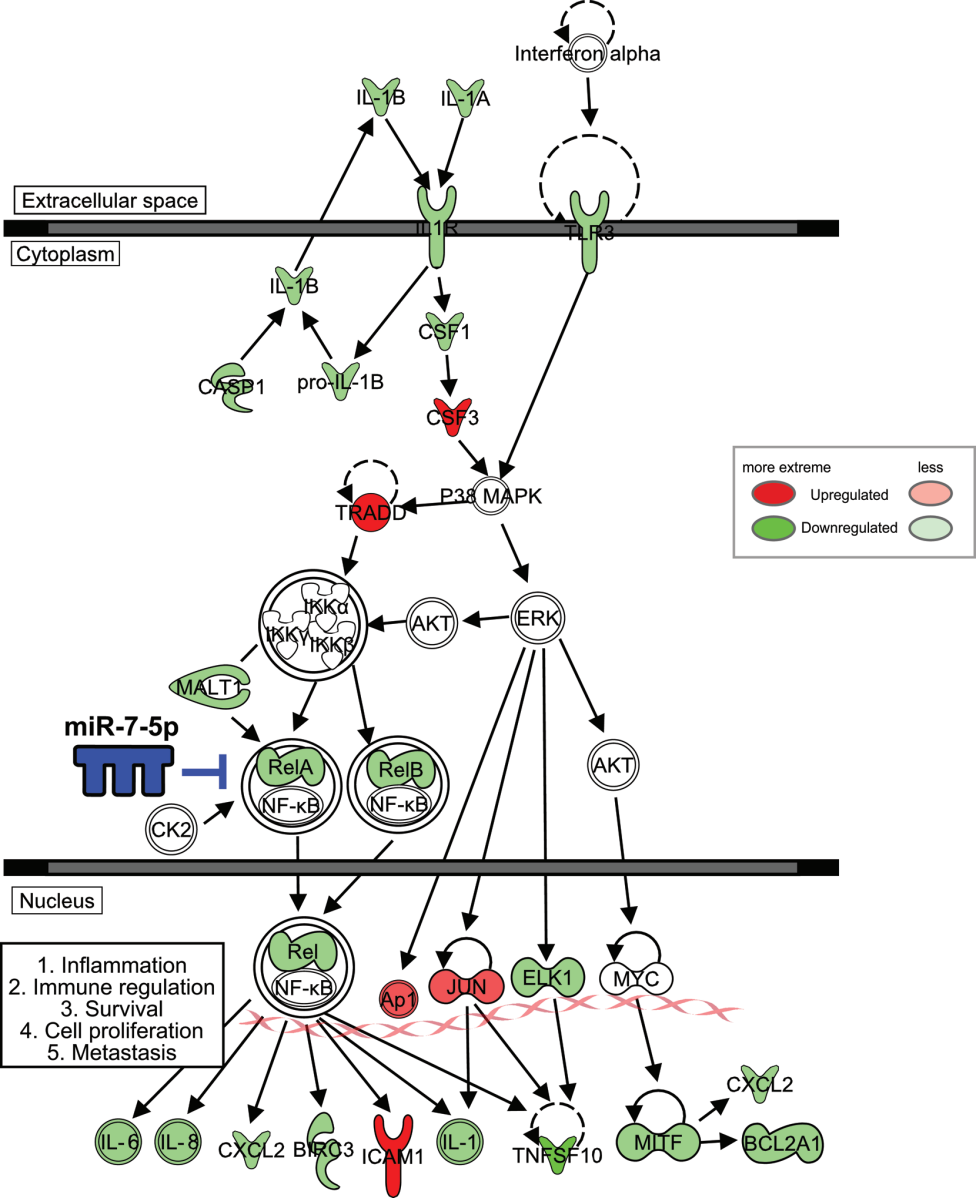
Coordinate regulation of NF-κB signaling in melanoma cells by miR-7-5p Schematic of genes downregulated (green) or upregulated (red) by miR-7-5p in melanoma, as a summary of data from the NF-κB PCR array and further validation by RT-qPCR and ELISA. IPA software was used to map common miR-7-5p-downregulated genes (shown in green) in WM266-4 and 1205Lu melanoma cells onto the canonical NF-κB pathway. The density of shading represents the degree of gene regulation by miR-7-5p.

In summary, we found miR-7-5p markedly suppresses the growth, migration and invasion of melanoma cells, and inhibits melanoma lung metastasis. Our data indicate miR-7-5p directly inhibits RelA expression, resulting in reduced NF-κB/RelA transcriptional activity. In turn, this leads to reduced expression and secretion of NF-κB downstream target molecules, including IL-1β, IL-6 and IL-8, which ultimately suppresses melanoma growth and metastasis (Figure 8). Thus, loss of miR-7-5p in melanoma promotes RelA overexpression, facilitating melanoma growth and metastasis and ultimately resulting in a worse clinical outcome. miRNA-based cancer therapies have entered the clinic, suggesting that they may represent a viable strategy to inhibit tumor growth and metastasis and extend survival in some patients. This study provides novel insight into the anti-melanoma activity of miR-7-5p, and support further preclinical development of miR-7-5p replacement therapy for patients with metastatic melanoma.

## MATERIALS AND METHODS

### Cell culture and reagents

WM266-4 and SK-MEL-2 cells were purchased from the American Type Culture Collection (ATCC), A2058 cells were a gift from Peter Klinken (Harry Perkins Institute of Medical Research) and 1205Lu cells were provided by NKH and MH. The identities of 1205Lu and A2058 cells were confirmed *via* short tandem repeat (STR) profiling by Cell Bank Australia (Westmead, Australia). Cells were maintained in RPMI 1640 supplemented with 10% fetal bovine serum (FBS) at 37°C in 5% CO_2_ and used within 20 passages of initial stock. Synthetic miRNA precursor molecules (Ambion) and FlexiTube siRNAs (Qiagen), were prepared in RNase free water including: hsa-miR-7-5p mimic (PM10047) and negative control miRNA #1 (miR-NC; AM17100), anti-microRNA-7-5p inhibitor (anti-miR-7-5p, MH10047); and anti-miR miRNA inhibitor negative control #1 (anti-miR-NC, AM17010); All Stars Negative Control (si-NC; SI03650318), si-RelA5 (SI00301672), si-RelA7 (SI02663094), si-RelA8 (SI02663101), si-RelA12 (si-RelA1, SI04437062) and si-RelA13 (si-RelA2 SI04437069). Transfections were performed with Lipofectamine 2000 (Invitrogen) and all assays included Lipofectamine (LF only) alone, as a transfection vehicle control.

### Cell proliferation and colony formation assays

Cell viability and 2D colony formation assays were performed as described [61], using miRNA precursors and anti-miRs at 5 nM for viability or 30 nM for clonogenicity or with siRNAs at 5 nM (final concentrations). For 3D colony formation assays, cells were transfected with miRNA precursors (30 nM) or siRNAs (5 nM) for 48 h, resuspended in a 1:1 mixture of RPMI 1640 and Matrigel Matrix (BD Biosciences) and added to 12-well plates, pre-coated with Matrigel. Cells formed colonies over a 14 d period, before staining with crystal violet for visualization.

### Cell cycle analysis

Cells were transfected with miRNA precursors (30 nM) or siRNAs (5 nM) for 72 h, trypsinized and fixed with ethanol, before propidium iodide (PI) staining and analysis by Flow cytometry on a BD Accuri C6 with Flowjo software (version 7.6.5).

### Migration and invasion assays

Cells were transfected with miRNA precursors (30 nM) or siRNAs (5 nM) for 48 h. Cell migration and invasion was monitored with a Real-Time Cell Analyzer dual-plate (RTCA-DP) xCELLigence instrument as described previously [30]. For invasion assays, CIM-plates were coated with Matrigel Matrix (diluted 1:40 in serum-free medium) 4 h prior to seeding cells and media with 20% FBS was used as a chemoattractant.

For wound healing assays, culture inserts (Ibidi) in 35mm μ-Dishes were used. Following transfection for 48 h with miRNA precursors and anti-miRs (30 nM) or siRNAs (5 nM), 70 μl of cell suspension (3 × 10^5^ cells/ml) was seeded into each well. After 24 h the inserts were removed, leaving a gap of 500 μm and wound closure was determined using ImageJ 1.48v imaging software (NIH).

The 3D spheroid invasion assay was performed as described previously [33, 64]. Briefly, cells were transfected with miRNA precursors (30 nM) or siRNAs (5 nM) for 24 h, seeded into 96-well plates coated with 1.5% agarose and incubated for 72 h to form single spheroids. The spheroids were then harvested, embedded into bovine collagen I (Cultrex) matrix and invasion monitored by microscopy.

### Animals

The metastasis study was conducted in male, 10-11 week old, NOD.CB17-Prkdcscid Il2rgtm1Wjl/SzJ (NSG) mice (Jackson Laboratory). Use of animals was in accordance with the guidelines of the University of Western Australia Animal Ethics Committee under approval numbers RA/3/100/1216.

### *In vivo* metastasis study

1205Lu cells were transfected with miRNA precursors (30 nM) for 48 h, at which time a total of 20 mice (randomized groups of 9 each for miR-NC or miR-7-5p, and 2 with non-transfected cells) each received tail vein injections of 1 × 10^6^ cells in 0.1 ml of PBS. Mice were monitored for 14 d, sacrificed and lungs excised, weighed and examined for visible tumor burden. Lungs were next stained with Bouin's solution for 24 h (lung parenchyma stains yellow and tumor nodules are white) and then paraffin-embedded, sectioned and stained with H&E. Five sections at various depths were imaged by microscopy and the 1205Lu lung tumor nodules manually quantified by three independent researchers under blind conditions using ImageJ software. The liver, heart and brain were also analyzed for presence of tumor nodules.

### cDNA microarray expression profiling and analysis

Total RNA was extracted from WM266-4 cells transfected with miR-NC or miR-7-5p precursor molecules (30 nM) for 24 h, using Qiazol reagent (Qiagen). The quantity and integrity of RNA samples was assessed using a 2100 Bioanalyzer (Agilent Technologies). Gene expression profiling by microarray hybridization was performed with three experimental replicates for each condition by the Australian Genome Research Facility (AGRF; Victoria, Australia) using a HumanHT-12 v4 array chip (Illumina). Signal intensity values underwent normalization, variance stabilization transformation and log_2_ transformation using the lumiR package of R Bioconductor (www.Bioconductor.org). ANOVA analysis of normalized probe intensities values was performed in Partek® Genomic SuiteTM software (version 6.6 build 6.12.0420). Probes with an unadjusted p-value of 0.05 or less and an absolute fold change of 1.5 or more between miR-NC and miR-7-5p samples were classified as being differentially expressed. Clustering and volcano plots showing the distributions and correlations of gene expression between miR-NC and miR-7-5p samples were produced using normalized data and the R ‘graphics’ package. A heat map comparing significant differential gene expressions between the miR-NC and miR-7-5p microarrays was produced with the R ‘gplots’ package. Ingenuity Pathway Analysis (Ingenuity Systems, Inc) was used to identify canonical pathways and cellular functions affected by miR-7-5p, some of which were presented figuratively using Ingenuity's Pathway Designer. TargetScan (Version 6.2: June 2012) was used to identify putative miR-7-5p seed sites within the set of differentially expressed mRNAs.

### Microarray data

The microarray data for WM266-4 cells transfected with miR-7-5p or miR-NC have been deposited in the Gene Expression Omnibus under Accession Number GSE77196.

### Publicly available microarray datasets

Clinicopathologic and tumor gene expression data from four cohorts of melanoma patients (all accessed June 2015) was analyzed. The Cancer Genome Atlas (TCGA) cohort consisted of 463 melanoma patients, 289 men and 174 women, with 77 stage I, 130 stage II, 172 stage III, and 24 stage IV tumors, and 60 melanomas of unknown staging, with a mean age of 58.4 years. The Cirenajwis cohort consisted of 214 melanoma patients, 124 men and 90 women, with a mean age of 62.3 years (Gene Expression Omnibus (GEO) accession number GSE46517 [35]). The Kabbarah cohort consisted of 81 melanoma patients, 54 men and 27 women, with 50 primary and 31 metastatic melanomas, and a mean age of 58 years (GEO accession number GSE46517 [36]). The Jayawardana cohort consisted of 74 melanoma patients, with 29 stage I, 29 stage II, and 20 stage III tumors, and 1 melanoma of unknown staging (GEO accession numbers GSE54467 and GSE59334 [37]).

### Reverse transcription and quantitative polymerase chain reaction (RT-qPCR)

Cells were transfected with miRNA precursors (30 nM) or siRNAs (5 nM) for 24 h and RNA isolated using Qiazol. For cDNA synthesis, 0.5 μg of total RNA was reverse transcribed using QuantiTect Reverse Transcription Kit (Qiagen). Quantitative PCR was performed with a Rotor-Gene 6000 thermocycler (Qiagen) or ViiA7 Real-Time PCR system (Life Technologies), using SensiMixPlus SYBR (Bioline). Primer sequences are listed in Supplementary Table S8. Validated primer sequences from PrimerBank were used where available [65]. Relative expression for each sample was calculated, taking into account PCR efficiency [66] with GAPDH or HPRT1 used as reference genes. A NF-κB pathway PCR array (RT^2^ Profiler PCR Array, SABiosciences, Qiagen) was performed with WM266-4 cells transfected with miR-NC or miR-7-5p for 72 h, according to manufacturer's instructions and analyzed with the PCR Array Data Analysis Software provided from the company.

### Western blotting

Protein lysates were prepared as described [67] from cells transfected with miRNA precursors (30 nM) or siRNAs (5 nM) for 72 h. 10-15 μg of each protein was resolved on 4-12% Bis-Tris gels (Invitrogen) and transferred to Immobilon-FL membranes (Millenium Science), that were probed with anti-RelA (#8242, Cell Signaling Technology), anti-phospho-RelA (#3033, Cell Signaling Technology), anti-PARP (#9532, Cell Signaling Technology), and anti-β-actin (ab6272, Abcam) antibodies. Antibody detection was performed using the Odyssey Infrared Detection System (Li-Cor), and band intensities normalized to β-actin loading control.

### Luciferase plasmid construction

Construction of the miR-7-5p perfect target reporter has been described previously [68]. Full-length wild-type (WT) and mutant (MT) *RelA* 3′-UTR reporter plasmids were synthesized by GenScript Inc. (Piscataway, NJ) to include the full length *RelA* 3′-UTR (nt 1919-2700 of GenBankTM accession number NM_021975) within the pmiR-REPORT firefly luciferase reporter vector (Ambion). Modifications were made to the *RelA* 3′-UTR at predicted miR-7-5p binding regions by replacing nt 2030-2036 (site A) and/or nt 2301-2308 (site B) with the sequence 5J9-CAG AAG GU-3′. All constructs were verified by DNA sequencing.

### Luciferase reporter assays

*RelA* 3′-UTR luciferase reporter assays were performed as described [67], and using miRNA precursor molecules at 2.5 nM final concentration. NF-κB transcriptional activity was determined by co-transfection of a NF-κB-driven luciferase reporter or a negative control reporter lacking transcriptional response elements (TREs) (Cignal Reporter Assay, SABiosciences, Qiagen), with miRNA precursor molecules (10 nM) or siRNAs (5 nM). Transfection efficiency was assessed using a green fluorescent protein (GFP)-expressing positive control plasmid. Lysates were assayed for firefly and *Renilla* luciferase activities 48 h post-transfection with the Dual Luciferase Reporter Assay System (Promega). Firefly luciferase activity was first normalized to *Renilla*, then each condition expressed relative to the negative control reporter.

### Enzyme-linked immunosorbent assay (ELISA)

1205Lu cells were transfected with miRNA precursors (10 nM) or siRNAs (5 nM) and tissue culture media or cytoplasmic protein extracts were harvested after 72 h. Commercially available kits were used to quantify expression of IL-1β (R&D Systems, DLB50), IL-6 (Abcam, ab100572) and IL-8 (Abcam, ab100575).

### Statistical and bioinformatic analyses

Gene expression data were analyzed using the R programming platform. Kaplan-Meier survival curves were produced using the R “survival” package. Cox regressions were calculated using the R “coxph” function from the “survival” package with ties addressed by Efron's method. Forest plots were generated using the R “forestplot” package. For Forest plots, Cox regressions and Kaplan-Meier survival curves, deaths were used as an endpoint. Analyses were adjusted for age and gender. The validity of proportional hazards assumptions were assessed by scaled Schoenfeld residuals [69]. Correlation coefficients were calculated using the R “cor.test” function. Unless otherwise indicated, all experiments are presented as a representative of three independent experiments and quantitative data shows mean ± SD. Student's *t*-test was used to compare test (miR-7-5p or si-RelA) to negative control and detect statistical significance. *P*-values < 0.05 were considered as statistically significant.

### Supplementary methods

#### Annexin V-FITC and PI apoptosis assay

WM266-4 cells were transfected with miR-7-5p or miR-NC at 30 nM for 3 d, then both floating and adherent cells (harvested by trypsinization) were pooled and stained with Annexin V-FITC and PI using the Annexin V FITC Apoptosis Detection Kit I (BD Biosciences, 556547), as per manufacturer's instructions and analyzed on a BD Accuri C6.

#### Caspase 3/7 assay

WM266-4 cells were transfected with miR-7-5p or miR-NC at 30 nM for 3 d, then caspase-3/caspase-7 activity was measured using the Caspase-Glo 3/7 assay (Promega, G8091) according to manufacturer's instructions. Relative apoptosis was determined by normalizing luminescence values to vehicle (LF only) control.

#### xCELLigence proliferation assay

1205Lu cells were transfected with miR-7-5p or miR-NC at 30 nM for 48 h. Cells were seeded in CIM-plates to measure migration and invasion (80,000 cells/well) and E-plates (5000 cells/well) for proliferation and loaded into the xCELLigence instrument. Cell Index was normalized to 5 h for proliferation to account for time for cells to adhere.

## SUPPLEMENTARY MATERIAL FIGURES AND TABLES


















