# Supplementary material for: microRNA-7-5p inhibits melanoma cell proliferation and metastasis by suppressing RelA/NF-κB
Source: Oncotarget. 2016 May 17;7(22):31663–80. doi: 10.18632/oncotarget.9421 (PMC5077967; doi:10.18632/oncotarget.9421)
Supplement: Supplementary file 1 [file oncotarget-07-31663-s001.pdf]

# microRNA-7-5p inhibits melanoma cell proliferation and metastasis by suppressing RelA/NF-κB

**Supplementary Material**

**A**

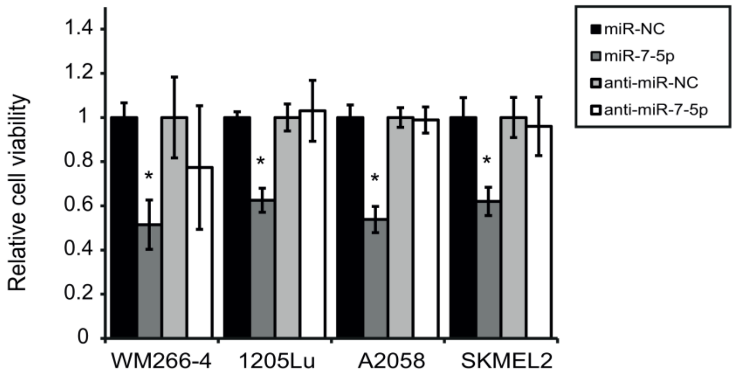


**Figure S1. Inhibition of endogenous miR-7-5p and effect on viability.** (A) Cell viability (MTS) assay at 5 d of WM266-4, A2058, 1205Lu and SK-MEL-2 cells transfected with miR-7-5p or anti-miR-7-5p or their respective negative controls at 5 nM final concentration. *, p-value < 0.05.

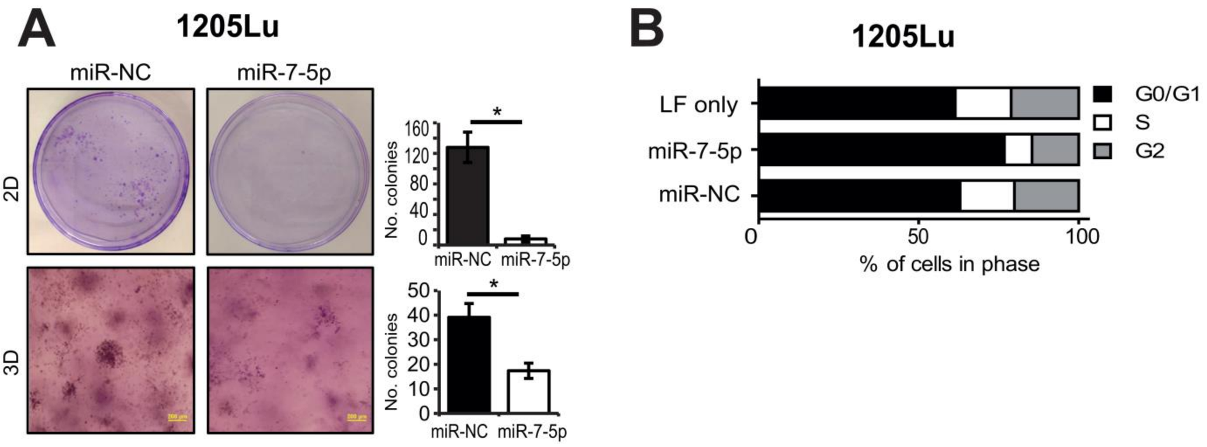


**Figure S2. Reduced colony formation and cell cycle progression in 1205Lu cells transfected with miR-7-5p.** (A) Representative images of 2D (plastic) and 3D (Matrigel) colony formation assay of 1205Lu cells transfected with miR-7-5p or miR-NC precursor molecules (30 nM), and then cultured for 21 d, fixed and stained with crystal violet. Scale bar = 200 µm. Number of colonies counted manually using ImageJ software. *, p-value < 0.001 (B) Flow cytometric cell cycle analysis of 1205Lu cells transfected with miR-7-5p or miR-NC precursor molecules (30 nM) for 3 d.

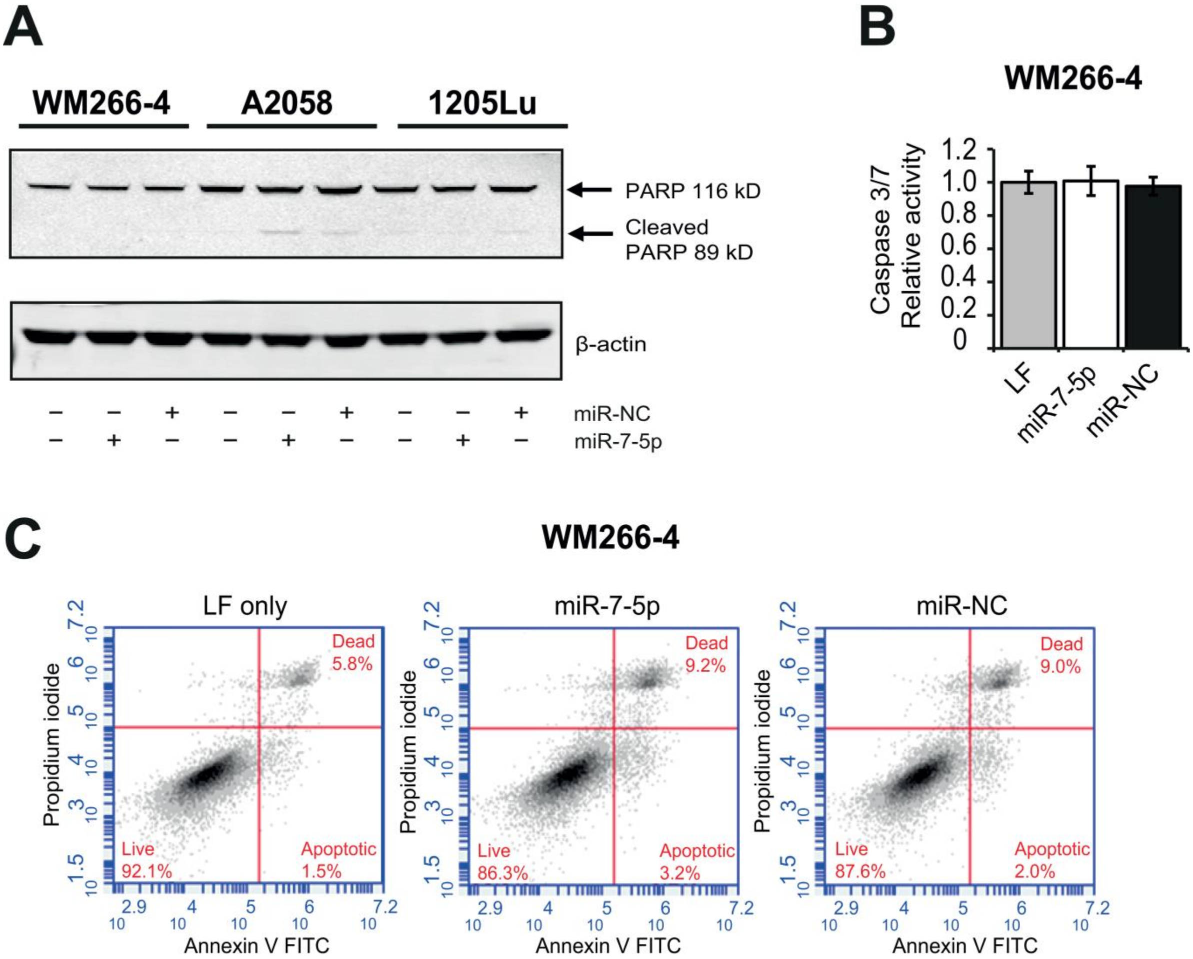


**Figure S3. Apoptosis analysis of melanoma cells following transfection with miR-7-5p.** (A) Western blotting analysis of PARP cleavage following transfection of miR-NC or miR-7-5p precursor molecules (30 nM) in WM266-4, A2058 and 1205Lu cells for 3 d. β-actin, loading control. (B) Caspase 3/7 glo assay of WM266-4 cells transfected with miR-7-5p or miR-NC (30 nM) for 3 d. (C) Annexin V FITC and PI staining of WM266-4 cells transfected with miR-7-5p or miR-NC (30 nM) and analyzed by flow cytometry at 3 d.

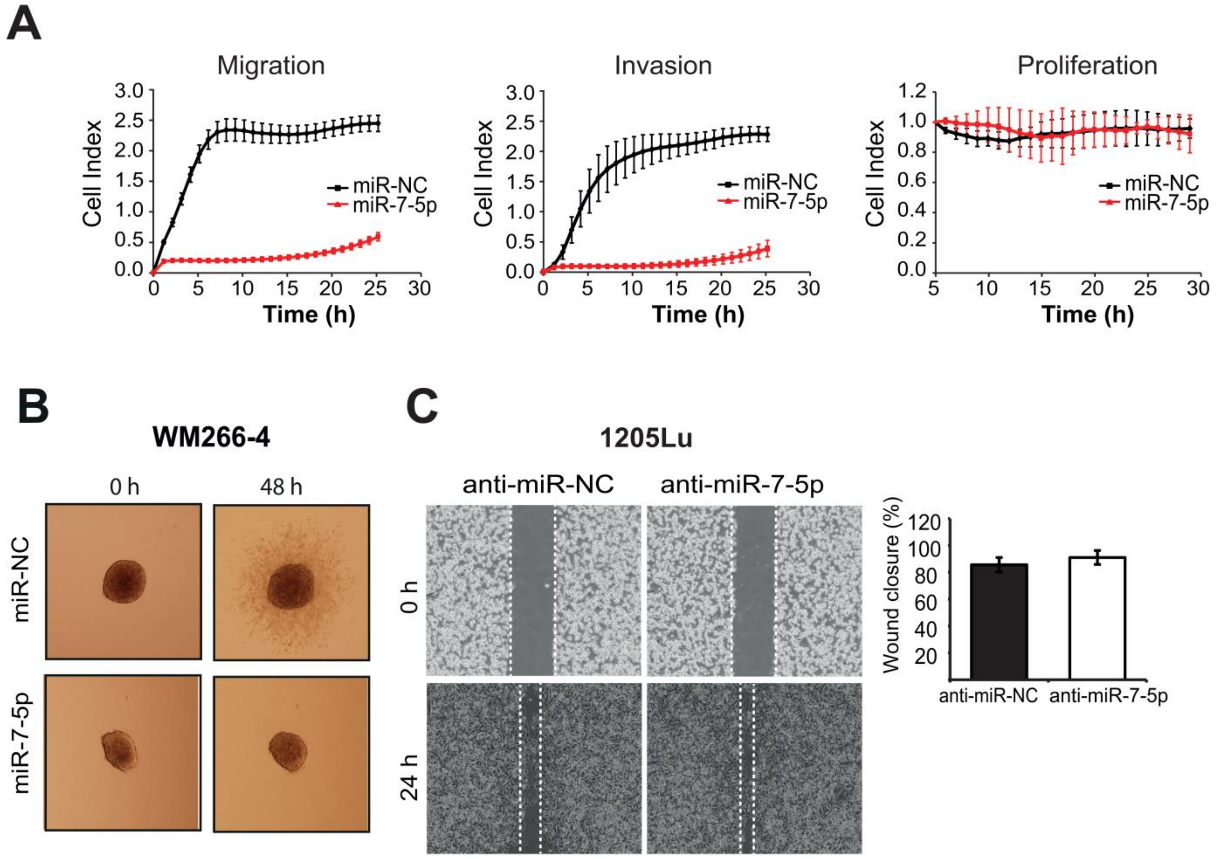


**Figure S4. Migration, invasion and proliferation analysis of 1205Lu and WM266-4 cells.** (A) xCELLigence real-time assays of migration, invasion and proliferation of 1205Lu cells following transfection with miR-7-5p or miR-NC (30 nM) for 48 h. (B) Representative images of spheroid invasion of WM266-4 cells transfected with miR-7-5p or miR-NC (30 nM) after 48 h following embedding in collagen (C) Wound closure assay of 1205Lu cells transfected with anti-miR-7-5p or anti-miR-NC (30 nM) for 48 h.

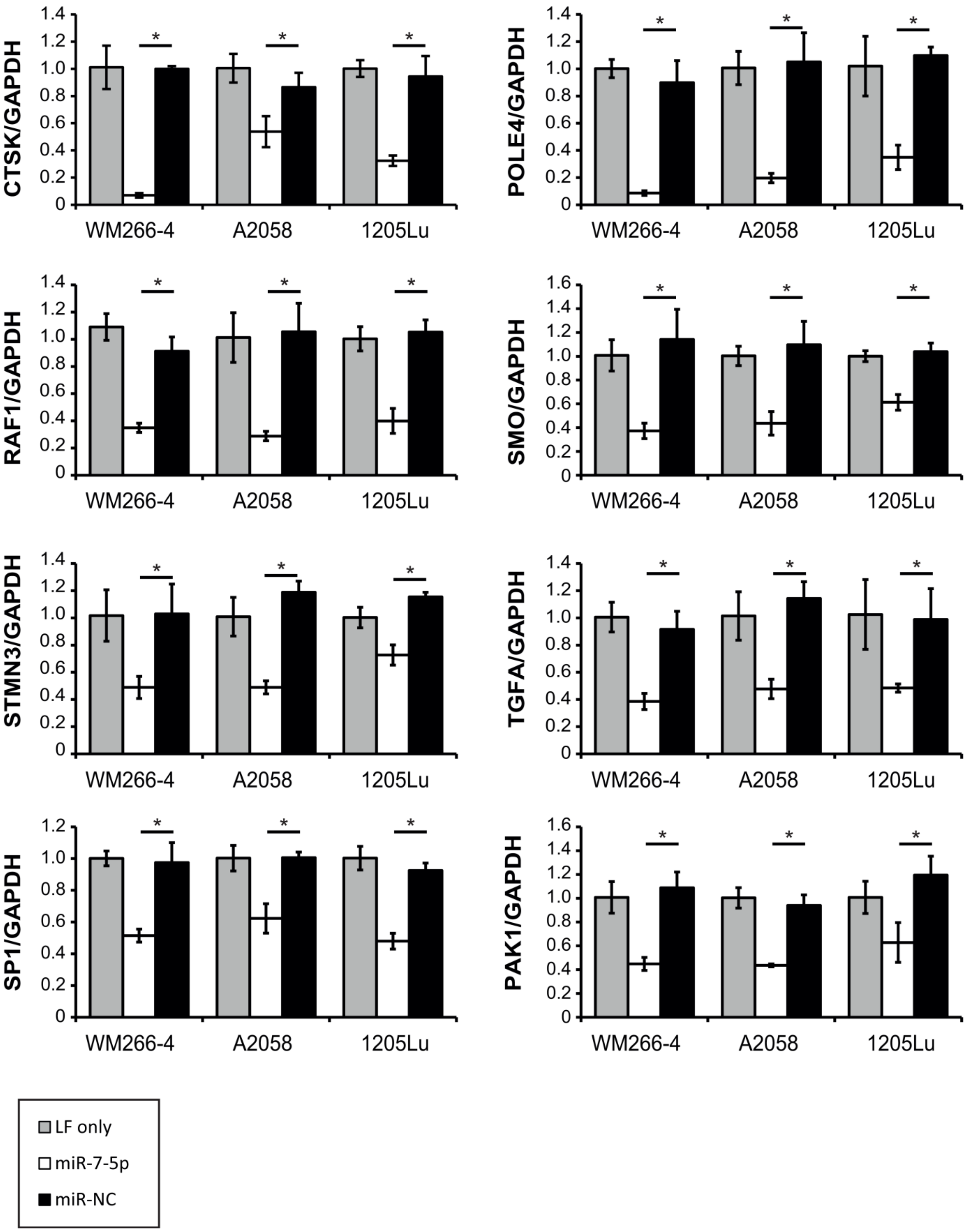


**Figure S5. RT-qPCR validation of miR-7-5p-downregulated genes.** RT-qPCR analysis of miR-7-5p-downregulated genes in WM266-4, A2058 or 1205Lu cells transfected with miR-7-5p or miR-NC precursor molecules (30 nM) for 24 h. *, p-value < 0.05.

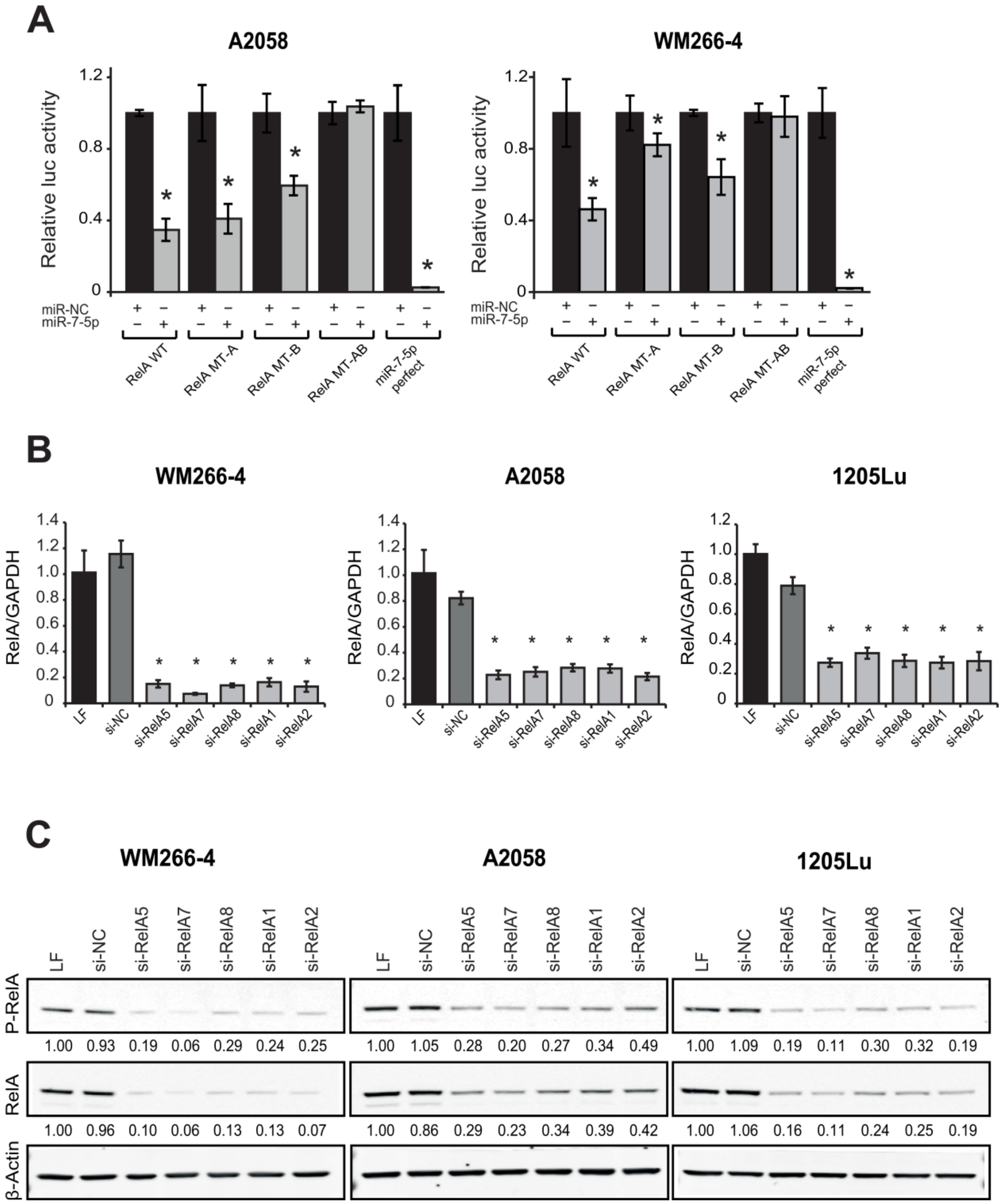


**Figure S6. RelA 3'-UTR reporter assays and validation of RelA knockdown.** (A) Luciferase reporter assay with A2058 and WM266-4 cells that were transfected with RelA 3’UTR reporter constructs or miR-7-5p perfect firefly-luciferase reporter, control *Renilla*-luciferase plasmid, and either miR-7-5p or miR-NC precursor molecules at 2.5 nM. *, p-value < 0.01. (B) RT-qPCR analysis of RelA mRNA for WM266-4, A2058 and 1205Lu cells

transfected with RelA or negative control siRNAs for 24 h at 5 nM. *, p-value < 0.01. (C) Western blotting analysis of RelA, P-RelA and β-actin protein 3 d post-transfection with RelA or negative control siRNAs at 5 nM. *, p-value < 0.01.

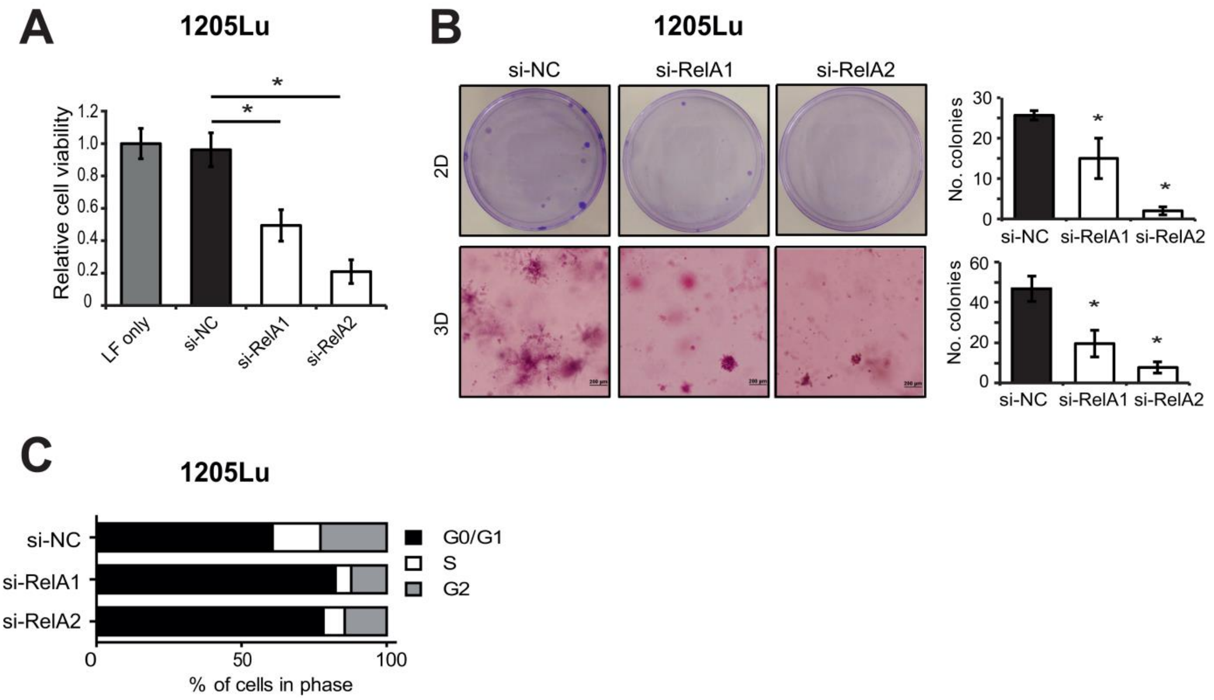


**Figure S7. Effect of RelA knockdown on 1205Lu melanoma cell viability, cell cycle, migration and invasion.** (A) Cell viability (MTS) assay of 1205Lu cells transfected with RelA siRNAs or si-NC (5 nM) for 5 d. *, p-value < 0.01. (B) Representative images of 2D and 3D colony formation assay of 1205Lu cells transfected with RelA siRNAs or si-NC (5 nM), and then cultured for 21 d, fixed and stained with crystal violet and the number of colonies counted manually. *, p-value < 0.001 (C) Flow cytometric analysis of 1205Lu cells transfected with RelA siRNAs or si-NC (5 nM) for 3 d.

**Supplementary Table S1: mRNAs upregulated by miR-7-5p in WM266-4 cells.** List of mRNAs identified by microarray analysis as significantly upregulated (p-value < 0.05) by greater than 1.5 fold in WM266-4 cells 24 h after transient transfection with miR-7-5p relative to miR-NC.

**Supplementary Table S2: mRNAs downregulated by miR-7-5p in WM266-4 cells.** List of mRNAs identified by microarray analysis as significantly downregulated (p-value < 0.05) by greater than 1.5 fold in WM266-4 cells 24 h after transient transfection with miR-7-5p relative to miR-NC.

**Supplementary Table S3. Genes downregulated in the WM266-4/miR-7-5p microarray that contain a miR-7-5p seed sequence.**

IPA pathway analysis tool was used to identify genes that were downregulated greater than 1.5 fold and contained a miR-7-5p seed region according to TargetScan V6.2.

**Supplementary Table S4. Functional pathways enriched for genes downregulated with miR-7-5p in WM266-4 cells.** IPA software was used to assign biological significance to the genes downregulated with miR-7-5p relative to miR-NC in WM266-4 cells, based on annotated functional pathways.

**Supplementary Table S5. Literature review of function of putative miR-7-5p target genes.** A literature review (PubMed) was conducted, linking putative miR-7-5p target genes to melanoma and metastasis.

**Supplementary Table S6. Correlations of miR-7 isoforms with expression of the putative miR-7-5p target genes in the TCGA melanoma cohort.** Pearson's correlation coefficient was used to calculate the association between miR-7-1, miR-7-2 and miR-7-3 expression with each of the putative target genes in the TCGA melanoma cohort.

**Supplementary Table S7. Genes up or downregulated in response to miR-7-5p overexpression in the NF-κB RT$^2$ PCR profiler array.** WM266-4 and 1205Lu cells were transfected with 30 nM miR-7-5p or miR-NC precursor molecules for 3 d. Bold genes were selected for further validation by RT-qPCR and fold change, confidence interval (CI) and p-value expressed from an average of three independent experiments.

**Supplementary Table S8: Primer Sequences.** Sequences of primers used for RT-qPCR validation of mRNA expression.
