# Supplementary material for: microRNA-7-5p inhibits melanoma cell proliferation and metastasis by suppressing RelA/NF-κB
Source: Oncotarget. 2016 May 17;7(22):31663–80. doi: 10.18632/oncotarget.9421 (PMC5077967; doi:10.18632/oncotarget.9421)
Supplement: Supplementary file 2 [file oncotarget-07-31663-s002.pdf]

Supplementary Table S1: mRNAs upregulated by miR-7-5p in WM266-4 cells.

Gene Symbol	Fold Change	p-value
ALDH9A1	1.500218	2.07E-05
COPS8	1.503097	1.30E-06
ISCU	1.506694	6.69E-04
ANKRD46	1.50828	5.62E-06
CEP135	1.508291	1.10E-04
HOXA4	1.510246	7.47E-04
UPF3A	1.512464	8.13E-05
ISCU	1.513847	9.47E-04
RP2	1.51424	3.18E-05
CEP55	1.514942	4.37E-05
H19	1.515252	2.90E-04
PPP2R3C	1.518449	1.15E-05
LCLAT1	1.520093	1.02E-04
SPECC1L	1.520297	2.48E-06
ZC3H7A	1.521982	1.43E-05
OSGIN1	1.522811	2.21E-03
FLRT3	1.523127	7.50E-05
WDR54	1.523221	1.18E-05
TPM1	1.523415	1.27E-04
HKDC1	1.523438	4.02E-06
MLX	1.525396	8.59E-06
MAP4K2	1.527159	1.08E-04
C20orf20	1.527995	2.41E-05
ARID5B	1.528829	1.19E-05
AZIN1	1.529535	2.53E-05
SLC35B3	1.530318	6.19E-07
SPC25	1.531898	2.89E-04
ST6GALNAC2	1.533134	5.55E-05
NCOA7	1.533375	3.77E-05
C9orf119	1.53531	3.85E-04
ARMCX1	1.5379	3.62E-07
SPA17	1.539049	1.65E-04
LOC728734	1.539261	1.96E-04
SEMA4F	1.54008	1.04E-04
PSMD7	1.540935	4.06E-06
YTHDC1	1.541275	2.41E-05
TOR1AIP1	1.541968	6.15E-05
LLPH	1.542103	2.17E-03
CFLAR	1.542551	2.81E-04
HPS5	1.542597	9.88E-06
TDG	1.543517	2.13E-04
C9orf123	1.544998	4.76E-07

SYNM	1.546626	6.03E-05
ROD1	1.546684	1.34E-04
RASSF1	1.549014	2.57E-05
ZDHHC6	1.54923	3.41E-05
PSMC3IP	1.551684	3.01E-05
AMACR	1.552848	3.67E-07
C15orf17	1.553905	2.52E-07
PRPSAP1	1.554229	1.75E-06
H3F3C	1.559432	1.66E-05
VBP1	1.560058	7.41E-07
IFT88	1.562342	1.80E-05
STK19	1.562448	2.27E-04
TFB1M	1.565649	1.03E-04
TRIB2	1.566082	6.66E-06
KATNA1	1.568182	9.15E-08
LCOR	1.568517	8.03E-05
DYRK2	1.570052	1.35E-06
PLDN	1.570415	3.41E-06
HNRNPR	1.570709	5.88E-05
SRP72	1.57083	3.57E-04
PBK	1.573062	5.44E-05
RNF182	1.573443	1.02E-04
TGFBR3	1.573926	1.43E-04
CAB39	1.574832	1.99E-04
C19orf2	1.574964	2.20E-06
DICER1	1.578885	2.58E-05
C5orf28	1.579227	2.63E-02
CTNNB1	1.580038	2.02E-04
METAP2	1.583897	2.15E-05
MZT1	1.584257	1.40E-05
UBE2T	1.585781	5.63E-05
LLPH	1.592755	2.17E-05
DYRK2	1.598205	9.74E-08
EPHB4	1.600823	5.95E-05
TBC1D19	1.603569	2.90E-06
CXorf57	1.605502	8.79E-06
AGPAT9	1.606812	2.02E-05
C16orf87	1.610061	2.82E-05
ERBB3	1.610985	2.56E-05
C16orf48	1.611555	8.65E-06
ZSWIM7	1.611841	1.36E-04
EML4	1.614678	1.23E-04
PJA1	1.61592	8.91E-07
VHL	1.619558	2.34E-07

BRCC3	1.621528	4.80E-07
TBC1D9	1.62296	1.83E-05
RNF14	1.622999	2.14E-07
CKAP2	1.623241	7.88E-06
DNAL4	1.623447	2.23E-06
SYTL2	1.623611	1.81E-05
MAD2L1	1.624925	5.79E-05
IGF2BP3	1.625163	1.98E-05
POU3F2	1.630748	1.54E-05
MRPL19	1.63188	1.64E-05
UBE2V1	1.632748	1.16E-06
PIAS2	1.63639	4.80E-07
C1GALT1	1.638292	4.31E-06
CYR61	1.64336	9.52E-06
ITGA4	1.643627	5.57E-04
TJP1	1.644941	1.51E-05
PSMC3IP	1.646208	2.14E-05
UBE2E3	1.650177	3.14E-04
KIF14	1.651298	5.47E-06
TBC1D4	1.652608	7.40E-05
SPECC1L	1.65455	8.10E-07
ACLY	1.657267	2.41E-04
UBE2Q2	1.66003	5.37E-05
NEIL3	1.66018	7.57E-06
POU3F2	1.660236	2.68E-07
GPR180	1.660663	3.44E-05
NEK2	1.664477	6.19E-04
HMGB2	1.66638	1.47E-05
CLUAP1	1.667334	6.21E-08
SRI	1.66778	8.13E-07
ADAM17	1.670951	1.52E-04
PDIA5	1.673457	3.46E-06
MAGEA12	1.673977	8.93E-05
RASSF1	1.676928	2.77E-07
CDC42SE2	1.676936	1.23E-05
HNRNPM	1.678083	2.00E-07
GALC	1.679366	5.58E-05
PSMC3IP	1.685947	1.34E-07
SENP2	1.686179	1.73E-05
HMGB2	1.686339	1.02E-04
CDKN2D	1.691843	1.04E-05
DENND5B	1.693054	3.59E-06
CRIPT	1.700795	4.35E-05
ANGPTL4	1.702901	1.26E-07

TOR1AIP1	1.704871	5.51E-06
ARL6IP6	1.706671	1.53E-05
CAV2	1.707681	7.40E-06
ACLY	1.710438	7.79E-05
HNRNPH2	1.710962	5.12E-06
CTNNB1	1.711193	2.84E-04
MTAP	1.71555	2.56E-08
CPOX	1.719113	2.76E-05
SMTN	1.719812	1.58E-03
HIRIP3	1.721586	1.35E-04
SYPL1	1.723553	3.22E-05
BIRC5	1.723713	4.77E-06
GPN1	1.734028	8.37E-08
LCOR	1.737932	8.35E-08
KIFAP3	1.743649	1.62E-05
SENP2	1.743702	1.89E-06
SRA1	1.749729	6.84E-06
MMP1	1.750538	3.51E-05
MACF1	1.755997	3.19E-06
GPR19	1.756073	9.48E-06
NDUFA6	1.75812	9.60E-06
CNIH4	1.75874	3.46E-05
CP110	1.765468	2.49E-07
CDK14	1.766438	5.03E-06
IMPA1	1.767822	1.45E-04
ETF1	1.769518	8.17E-07
FBXO33	1.778737	2.88E-05
HMGCS1	1.780099	8.76E-04
RSL24D1	1.780442	5.90E-07
HNRNPM	1.780499	1.03E-05
TJP1	1.785846	4.76E-06
RRM2	1.792213	2.06E-04
HCCS	1.813681	5.84E-06
RNF14	1.814703	1.52E-04
SCML1	1.819322	5.82E-06
SYTL2	1.826395	3.07E-07
PPM1B	1.835744	2.32E-06
GAL3ST4	1.846842	2.86E-08
NT5E	1.847701	6.23E-06
PAG1	1.847728	5.32E-08
HNRNPM	1.848594	7.84E-06
HNRNPR	1.855392	8.85E-07
ODF2L	1.856864	8.03E-08
AIDA	1.861889	1.39E-04

MAP2K1	1.865599	1.37E-06
SRI	1.869579	3.34E-08
IDI1	1.874999	3.87E-06
GXYLT1	1.880626	1.04E-06
ITGA6	1.881398	3.51E-05
SRI	1.881615	2.82E-08
PMM1	1.884533	3.02E-07
ETF1	1.901352	6.69E-07
CAV2	1.907688	2.29E-07
NUP35	1.920402	1.45E-07
HNRNPR	1.930669	1.13E-06
PROSC	1.93308	7.86E-07
EIF2S1	1.939538	8.60E-06
ATPAF1	1.945914	8.19E-07
CTGF	1.948971	4.65E-05
C7orf42	1.952727	2.24E-05
BTG3	1.961712	3.50E-07
VKORC1L1	1.962747	1.90E-08
CKAP2	1.981484	6.56E-05
SLC35A1	1.994111	2.32E-07
RHOB	2.003638	1.21E-06
TXNDC5	2.007702	3.44E-05
MAGEA12	2.016249	2.74E-08
PITHD1	2.022927	3.28E-09
ARPC4	2.02656	5.94E-08
ARPC4	2.030432	6.47E-06
RIT1	2.039858	5.33E-06
C12orf76	2.043936	1.18E-08
MAPK1IP1L	2.060014	5.43E-08
RDH11	2.062459	1.24E-06
IKBIP	2.068561	1.28E-05
ARMCX1	2.071935	3.25E-06
FECH	2.072688	7.44E-07
YWHAG	2.078332	3.51E-07
ANGPTL4	2.098909	3.57E-06
C5orf15	2.119844	5.31E-06
RTCD1	2.126101	1.30E-08
GNA13	2.12914	6.45E-06
RHPN2	2.138565	3.21E-09
GNB1	2.142676	8.27E-08
RTCD1	2.156772	9.97E-07
YWHAG	2.165631	5.60E-08
RAB11FIP1	2.195026	4.67E-06
PNMA1	2.204723	7.93E-08

C5orf51	2.225137	1.16E-07
C5orf51	2.238236	1.12E-06
RDH11	2.246545	4.78E-06
RDX	2.251674	4.59E-06
NUP35	2.305793	4.67E-08
SLC2A3	2.321157	9.45E-07
SYT11	2.341349	8.84E-06
C12orf76	2.351214	1.33E-09
NARF	2.355279	1.21E-07
CTGF	2.383213	1.17E-05
NPTN	2.423185	2.48E-08
COMMD10	2.442951	2.59E-09
ICMT	2.529495	7.74E-08
NPTN	2.54421	1.65E-07
RAB8B	2.547302	8.78E-07
SCML1	2.561286	9.37E-08
BTG3	2.622336	3.92E-07
CYBRD1	2.623774	4.31E-07
SNRPC	2.704316	4.97E-08
CAV2	2.71329	9.33E-10
COMMD10	2.78941	3.03E-08
CYBRD1	2.958742	1.05E-08
SNRPC	3.217349	2.83E-09
TMEM2	3.74921	5.24E-08
