# Supplementary material for: microRNA-7-5p inhibits melanoma cell proliferation and metastasis by suppressing RelA/NF-κB
Source: Oncotarget. 2016 May 17;7(22):31663–80. doi: 10.18632/oncotarget.9421 (PMC5077967; doi:10.18632/oncotarget.9421)
Supplement: Supplementary file 3 [file oncotarget-07-31663-s003.pdf]

Supplementary Table S2: mRNAs downregulated by miR-7-5p in WM266-4 cells.

Gene Symbol	Fold Change	p-value
POLE4	-6.86147	1.77E-09
CRTAP	-6.72923	1.23E-09
CTSK	-6.27368	3.57E-11
PSME3	-5.86241	4.52E-09
PSME3	-5.80604	1.44E-10
POLE3	-4.63569	1.24E-11
CKAP4	-4.22576	1.72E-09
LITAF	-4.12253	4.75E-08
C18orf10	-3.95152	6.94E-10
TMEM158	-3.91059	1.16E-09
ACO2	-3.75155	1.28E-07
C18orf10	-3.73459	7.53E-08
TMEM43	-3.68198	3.70E-08
SMARCD1	-3.59651	7.90E-08
BCL2L12	-3.54424	2.05E-08
CNRIP1	-3.54388	6.91E-09
SCARB2	-3.52772	1.25E-10
GLO1	-3.50827	9.73E-09
LBH	-3.4838	1.46E-08
DYM	-3.46537	2.52E-08
DHCR24	-3.46082	5.40E-07
BCL2L12	-3.45895	2.40E-05
TMEM97	-3.43507	1.21E-08
DAZAP2	-3.33766	1.13E-09
RUSC1	-3.26108	9.68E-09
AGK	-3.18013	3.17E-10
VPS26A	-3.11009	3.40E-08
RRP7A	-3.09954	1.15E-09
RCC2	-3.05653	3.24E-08
EIF2S3	-3.0527	1.19E-08
UBQLN4	-3.02856	3.27E-09
SLC35A4	-2.99728	1.78E-07
ZDHHC9	-2.99471	2.75E-09
TSEN34	-2.96632	3.71E-09
TIMP3	-2.95049	9.18E-07
TMED9	-2.91086	2.46E-09
ARMC10	-2.9025	3.32E-09
RNF181	-2.87154	1.67E-07
CNN3	-2.86512	2.41E-08
AUP1	-2.84888	3.62E-08
PIGH	-2.84378	4.67E-09

SLC39A3	-2.82561	1.01E-06
FAM46C	-2.81157	1.65E-07
CALM3	-2.80305	1.73E-09
EHD1	-2.79865	1.20E-07
MGLL	-2.79167	8.25E-09
CYBASC3	-2.75985	2.81E-07
SKP1	-2.75531	6.98E-08
ARSB	-2.74111	5.74E-07
TMED10	-2.74096	1.51E-06
C1orf198	-2.72783	5.64E-07
SIGMAR1	-2.71809	1.43E-06
TGOLN2	-2.70253	3.21E-08
NDUFA4	-2.68953	1.36E-09
AKR7A2	-2.66653	2.92E-08
GAD1	-2.6631	3.84E-08
MFSD10	-2.66304	4.53E-07
SLC25A15	-2.6517	7.15E-08
OLFML2A	-2.65125	1.66E-09
SLC9A1	-2.64893	1.26E-08
NR1H2	-2.6485	9.36E-08
PI15	-2.64268	2.96E-08
CITED4	-2.64189	9.93E-07
GRINA	-2.62131	1.96E-06
HIATL1	-2.61088	7.85E-07
ELK1	-2.60808	9.44E-09
AKR7A3	-2.60688	4.24E-10
GAS7	-2.60066	2.08E-08
SHISA5	-2.59383	5.60E-06
RAF1	-2.59379	1.77E-07
SEPW1	-2.58376	9.84E-08
RNF144A	-2.58116	6.07E-09
SUPT4H1	-2.5771	4.06E-08
RNF144A	-2.55689	1.85E-08
XPR1	-2.5476	3.54E-07
UBE3C	-2.54192	5.29E-07
SIRPA	-2.53249	7.71E-09
UBE3C	-2.522	1.94E-07
PIK3R2	-2.51883	4.09E-06
MGLL	-2.47964	2.44E-09
CTDSP2	-2.45749	7.07E-09
XPR1	-2.45535	1.39E-07
ATP1B3	-2.44044	7.30E-07
C20orf4	-2.43756	4.31E-07
SPATS1	-2.43372	2.30E-07

ZYX	-2.43112	2.39E-07
NBL1	-2.43032	1.86E-05
MFSD5	-2.42972	2.67E-07
ZMIZ1	-2.42806	5.54E-07
TSEN34	-2.41153	9.78E-07
GLS	-2.41055	7.69E-09
KPNA6	-2.40658	1.10E-04
SIGMAR1	-2.39271	1.24E-08
SIGMAR1	-2.3832	9.01E-06
RNF114	-2.38309	1.17E-09
NUDCD3	-2.37332	3.84E-07
EIF2AK1	-2.36616	3.74E-08
PQLC1	-2.35822	4.37E-08
CNRIP1	-2.35037	4.63E-09
TRIML2	-2.34907	4.26E-08
MX2	-2.34319	4.11E-07
PATL1	-2.33737	5.62E-08
PAQR4	-2.33507	8.77E-07
CTDSPL	-2.32824	1.84E-07
ZNF395	-2.32656	3.03E-06
ZYX	-2.31123	2.13E-06
PRMT2	-2.30951	4.20E-07
RAB5B	-2.29708	7.10E-06
GRINA	-2.29654	4.05E-06
STX5	-2.28576	2.42E-07
NBL1	-2.28499	1.25E-06
SNAP29	-2.2837	3.21E-08
TMEM179B	-2.27629	6.44E-07
AP3M1	-2.2756	1.77E-06
TRPV2	-2.27067	1.72E-08
COMMD7	-2.26953	5.68E-08
C20orf24	-2.26711	2.78E-08
SDC4	-2.26114	8.45E-09
RNF5	-2.26109	4.82E-09
TRIM8	-2.25988	2.91E-08
YWHAB	-2.25424	8.29E-09
EPB41L3	-2.25205	3.87E-07
AMMECR1	-2.24772	3.39E-09
PI15	-2.24393	6.07E-07
NPTX1	-2.2429	3.98E-05
PIGH	-2.23916	5.09E-08
TMEM179B	-2.22362	6.93E-08
VGLL4	-2.19766	2.15E-05
GHDC	-2.19513	2.27E-05

DCAF7	-2.18965	9.80E-06
CRTAP	-2.17854	6.68E-06
PDXDC1	-2.16828	1.14E-06
DCAF12	-2.16117	3.18E-07
C3orf21	-2.13698	1.75E-06
CMTM7	-2.1353	4.05E-07
UBE2D4	-2.13236	1.12E-07
SLC25A39	-2.12009	1.35E-08
GPM6B	-2.10705	1.17E-09
UBAP1	-2.10512	3.92E-08
RPL22	-2.09881	1.39E-08
HMGN4	-2.09842	8.83E-09
ADPRHL2	-2.09192	5.66E-06
PHF16	-2.08892	8.22E-06
RHBDF2	-2.08673	4.10E-08
PFN2	-2.07903	5.43E-07
TTLL12	-2.07556	1.15E-06
FAM82A2	-2.0731	4.73E-06
AP3M1	-2.07087	1.19E-06
TMEM69	-2.06916	3.05E-07
FAM89B	-2.06271	3.87E-07
CAV1	-2.06251	6.65E-06
ZNF828	-2.06112	1.01E-06
HPCAL1	-2.05848	2.72E-08
PLAGL2	-2.05494	5.74E-08
PRMT2	-2.05467	2.34E-06
PLOD1	-2.0525	8.63E-08
TSGA14	-2.05024	2.89E-08
DNAJC15	-2.04867	9.28E-08
SRF	-2.04806	2.84E-07
RNF114	-2.04264	1.85E-08
SLC6A10P	-2.03875	1.42E-05
PAK2	-2.03627	1.59E-07
RNF38	-2.0338	2.34E-07
SLC2A10	-2.03324	5.18E-06
IDE	-2.03255	2.88E-08
RPL15	-2.02949	1.33E-07
UHRF1	-2.02905	4.49E-08
HSPA5	-2.02291	2.39E-07
ALG3	-2.02064	8.10E-06
CDC37	-2.01885	1.39E-07
SKP1	-2.01245	2.16E-08
L1CAM	-2.01032	1.86E-03
ILF3	-2.00983	9.56E-06

OSBP	-2.00549	5.75E-07
STK32B	-2.00303	3.48E-07
RNF5P1	-2.00144	7.48E-08
SIGMAR1	-1.99852	2.56E-07
TMEM150A	-1.99794	1.19E-05
LPCAT1	-1.99516	6.86E-07
TMEM14C	-1.9919	5.02E-07
PLEC	-1.98866	1.09E-04
NHLRC3	-1.98444	2.94E-08
GAD1	-1.98415	5.04E-08
UBE2J1	-1.98034	1.46E-06
WBP2	-1.97874	3.64E-06
GPAA1	-1.97835	2.11E-07
COL1A2	-1.97392	1.30E-05
TYRO3	-1.97152	8.04E-07
SLC39A3	-1.95474	1.09E-06
BCKDK	-1.95005	2.40E-07
NDFIP2	-1.94969	1.09E-06
SPNS1	-1.94906	4.18E-06
SPIRE1	-1.9485	1.45E-07
PIP4K2C	-1.94656	1.83E-08
DHCR7	-1.94189	1.66E-07
PLXNA1	-1.93956	1.75E-06
TRIM25	-1.93817	3.03E-07
CYB5R2	-1.93669	2.31E-06
SCAP	-1.93494	3.37E-06
CNO	-1.93445	1.45E-07
STMN3	-1.92818	7.02E-06
SNCA	-1.92713	5.90E-07
TSPAN10	-1.92395	5.53E-06
SLC25A23	-1.92228	1.51E-05
ENO2	-1.91874	9.02E-07
ADO	-1.91801	2.70E-08
TMEM14C	-1.91443	1.85E-07
PPP1R11	-1.91201	8.08E-08
ARHGAP1	-1.9061	5.37E-07
SLC35A5	-1.90128	3.09E-06
TBCD	-1.90055	3.62E-05
LOC653506	-1.90009	3.11E-08
EIF2B3	-1.89909	2.02E-07
ARF4	-1.89706	7.33E-07
RIPK4	-1.89689	5.53E-06
C10orf90	-1.89426	8.55E-06
ITPKB	-1.88975	6.20E-05

ORAI1	-1.88899	5.04E-08
TBC1D2B	-1.88793	2.38E-06
FRMD8	-1.8848	7.56E-09
LANCL1	-1.8818	1.37E-05
CISD2	-1.8814	2.53E-07
GNA12	-1.87671	2.43E-08
TM9SF1	-1.87497	2.45E-07
PCLO	-1.87427	2.03E-05
MT1X	-1.874	3.93E-06
C10orf57	-1.87099	1.04E-07
APLP2	-1.86994	1.13E-06
CMTM7	-1.86466	3.49E-06
NCEH1	-1.86427	1.18E-07
GALE	-1.86327	3.97E-07
PSRC1	-1.86285	3.55E-08
UBXN2B	-1.85738	3.23E-07
CHEK1	-1.8558	1.89E-07
SLC43A3	-1.85504	1.36E-04
ATF5	-1.85439	1.13E-05
ATP9A	-1.85407	3.53E-06
MMACHC	-1.85256	1.33E-04
PRKRIR	-1.8483	8.47E-05
NOL6	-1.84551	7.41E-06
SDHC	-1.84467	1.42E-06
IGSF3	-1.84177	8.03E-06
TCF12	-1.84146	3.73E-06
TGM2	-1.84016	1.88E-05
MPDU1	-1.83706	2.23E-05
TMEM93	-1.83536	4.41E-06
PDXK	-1.83458	1.10E-06
TOMM40L	-1.82312	2.25E-07
PEX19	-1.82255	2.12E-06
SEPT2	-1.82233	2.44E-05
SLC43A3	-1.81694	6.41E-06
EMX2	-1.81644	1.33E-06
PIK3CB	-1.81484	1.54E-05
ACSL4	-1.81207	1.13E-04
SH3BP4	-1.81188	1.58E-07
AMMECR1	-1.81055	7.82E-07
PHC2	-1.80913	1.09E-05
TMEM134	-1.80816	1.85E-07
UBE2Z	-1.80613	2.65E-06
PURB	-1.80278	1.17E-07
PRIC285	-1.80263	2.37E-05

LANCL1	-1.80227	1.27E-06
MLLT6	-1.80046	9.99E-07
RPS19BP1	-1.79869	6.16E-05
VDAC1	-1.79826	1.19E-05
STX5	-1.79804	9.42E-06
MPDU1	-1.79795	1.55E-06
C12orf49	-1.79658	1.83E-07
C1orf21	-1.79355	9.71E-07
CBL	-1.79148	5.70E-07
NPAS1	-1.79079	2.12E-07
SIRPA	-1.78832	4.46E-06
TRIB1	-1.78824	6.92E-05
UBE2N	-1.78736	3.52E-07
MRPL10	-1.78685	8.68E-06
CMTM7	-1.78622	3.10E-07
SEL1L3	-1.78292	2.28E-06
EXTL3	-1.78276	2.61E-06
BCL9	-1.78237	3.24E-07
ZBTB4	-1.78049	6.12E-05
HYAL2	-1.77792	2.12E-05
EFHD1	-1.77762	9.14E-08
TUSC2	-1.77725	2.91E-08
ST6GAL1	-1.77626	7.38E-06
SRSF4	-1.77609	4.05E-06
C15orf57	-1.77486	7.00E-08
NOTCH2	-1.7724	1.60E-05
SLC35C2	-1.77234	1.32E-06
SNCA	-1.7723	2.01E-08
PRR7	-1.76951	1.54E-05
ARSB	-1.76909	6.46E-08
BMP8B	-1.76458	1.90E-05
CNRIP1	-1.76436	8.03E-08
IGSF3	-1.76104	1.53E-07
SLC25A19	-1.7579	2.78E-06
NIF3L1	-1.75389	9.92E-08
C20orf24	-1.74956	1.26E-05
SLC27A1	-1.74487	1.16E-03
GPR56	-1.74422	8.95E-05
ANKS1A	-1.74329	1.16E-04
TCF12	-1.74313	1.93E-05
TOMM6	-1.74253	6.01E-05
TMEM134	-1.74136	3.14E-06
GPR56	-1.74135	1.16E-05
CMTM8	-1.74079	4.84E-05

ACSS1	-1.73947	9.15E-08
SNORA61	-1.73695	5.14E-05
PDE7B	-1.73154	1.28E-05
CD99L2	-1.72939	5.85E-07
GTF2E2	-1.72782	3.88E-06
EGR3	-1.7267	1.20E-07
SRM	-1.72659	1.71E-07
PRICKLE4	-1.72482	4.46E-05
TMEM184B	-1.72266	2.00E-04
WASF2	-1.72239	6.54E-04
FLOT2	-1.72218	4.06E-07
TMED10P1	-1.71981	9.69E-07
RGS4	-1.7183	3.06E-07
HECTD3	-1.71695	5.81E-06
SETD8	-1.71599	4.50E-06
SEPT2	-1.71594	3.94E-06
CAV1	-1.7158	5.25E-06
KCTD12	-1.71436	1.55E-05
SLC44A1	-1.7142	1.63E-05
CRTC3	-1.71174	1.81E-06
C9orf78	-1.71115	3.51E-05
DOLK	-1.71022	5.01E-05
ITPRIPL2	-1.70979	8.25E-06
SSU72	-1.70875	3.64E-06
PTK2	-1.70658	1.95E-06
C1orf85	-1.70489	2.24E-06
TROVE2	-1.70464	8.61E-07
TMEM93	-1.70359	9.15E-06
GREB1	-1.70339	8.15E-07
C3orf39	-1.7003	1.24E-04
ETV4	-1.70022	2.93E-05
TSGA14	-1.69661	5.25E-07
ADAM19	-1.69537	3.91E-05
DHX33	-1.69484	8.25E-05
FBXO2	-1.69133	2.24E-04
TSKU	-1.68646	5.56E-06
MAZ	-1.68517	2.05E-03
PSEN2	-1.68466	1.16E-06
DHCR7	-1.68155	1.49E-06
BRWD1	-1.67904	1.78E-04
ADCY9	-1.67843	3.05E-05
SERF2	-1.67485	7.08E-08
SLC25A15	-1.67321	2.16E-06
DENND4B	-1.67187	3.18E-05

CALU	-1.67016	1.61E-05
MRAP2	-1.66881	1.43E-06
FYCO1	-1.66779	6.18E-07
ZNF275	-1.6667	5.56E-06
ATP2C1	-1.66573	2.71E-06
C11orf24	-1.66361	1.95E-05
CASP2	-1.66308	2.41E-07
RNF40	-1.6627	8.20E-05
FAM203B	-1.66252	4.69E-07
SRGAP2	-1.6616	4.79E-05
EXOSC2	-1.66022	3.05E-06
DCT	-1.65898	9.87E-06
C20orf177	-1.6575	9.62E-07
FGF13	-1.65606	2.15E-06
PIGS	-1.65576	4.44E-07
VPS26A	-1.65175	3.06E-06
HRK	-1.65054	1.59E-04
TAPBP	-1.64762	1.69E-04
WDR4	-1.64667	1.20E-05
STOM	-1.64594	4.86E-06
GNPTAB	-1.64205	1.29E-05
COG2	-1.64129	2.68E-05
USP39	-1.63965	4.40E-07
MICALL1	-1.63616	4.34E-06
RHBDF2	-1.63603	2.52E-07
CBLB	-1.63545	1.34E-06
NPRL3	-1.6354	7.11E-05
SRGAP2	-1.63296	2.23E-05
CANT1	-1.63268	9.28E-04
C20orf24	-1.6325	1.26E-06
VPS4A	-1.63174	1.65E-05
PAK1	-1.63141	1.74E-06
SH2B3	-1.63138	2.55E-04
TTYH3	-1.631	2.73E-05
TMEM214	-1.63007	7.31E-04
NEU1	-1.63002	1.08E-05
CAPZA1	-1.62994	5.68E-06
FBXL18	-1.62905	6.61E-06
SLC35C2	-1.62756	1.98E-07
SPRY1	-1.62729	1.28E-05
PGAP2	-1.62713	7.92E-07
SLC6A15	-1.62695	4.47E-07
GOLGA7B	-1.62596	6.78E-07
ACTR3	-1.62359	7.43E-05

AGPAT1	-1.62349	1.25E-03
CAMK2D	-1.62272	1.01E-06
JOSD1	-1.62184	1.65E-06
CNOT8	-1.62172	8.64E-07
TBC1D13	-1.62122	3.60E-06
AVPI1	-1.62119	5.36E-08
PLK4	-1.61961	1.21E-04
DPYSL2	-1.6196	1.55E-06
HSPBP1	-1.61828	1.49E-05
MAFG	-1.61711	7.47E-05
TMEM219	-1.61655	2.52E-06
RAB11FIP5	-1.6159	5.67E-07
NPTX1	-1.61583	8.36E-04
ATG7	-1.6151	3.68E-05
ST8SIA5	-1.61494	6.72E-05
OXSR1	-1.61463	1.16E-05
KIAA0114	-1.61283	2.65E-04
AIF1L	-1.6128	2.86E-07
RYK	-1.61194	1.87E-06
C1QTNF6	-1.61183	2.91E-07
MAU2	-1.61052	1.64E-06
SLC35F1	-1.61015	3.71E-06
TMC6	-1.61013	3.11E-05
CBL	-1.60729	2.89E-05
ANKFY1	-1.60533	2.56E-05
ERGIC1	-1.60513	4.38E-04
KIAA0247	-1.60403	1.36E-07
METTL7B	-1.60368	2.37E-06
MKNK2	-1.60326	1.45E-04
GAB2	-1.60192	1.32E-04
CAPN5	-1.60167	5.76E-05
REPIN1	-1.60147	1.01E-03
DSCR3	-1.60092	3.29E-07
HN1L	-1.6002	6.39E-07
RRP1B	-1.59878	1.80E-04
SLC20A2	-1.59859	3.90E-05
SLC45A3	-1.59816	1.04E-07
ZC3H3	-1.59792	2.10E-06
GYG2	-1.59748	5.09E-05
ARMC10	-1.59549	9.95E-05
PHACTR4	-1.59474	4.01E-06
SORT1	-1.59298	1.43E-05
SERPINH1	-1.59168	1.05E-04
MGAT2	-1.59141	8.14E-06

SPTY2D1	-1.59129	7.11E-06
PNKD	-1.59058	1.84E-04
SERP1	-1.58941	1.47E-06
TRAM2	-1.58934	7.92E-06
RELA	-1.58849	3.91E-06
CXXC5	-1.58818	6.07E-07
DBNL	-1.58767	8.16E-06
MCRS1	-1.58745	1.65E-06
MT2A	-1.58719	1.31E-05
TRAK2	-1.5868	1.26E-04
UBIAD1	-1.58647	1.10E-05
REXO1	-1.58496	3.14E-06
MT1A	-1.58492	4.38E-06
KDM5B	-1.58489	1.60E-05
C9orf86	-1.58453	9.79E-07
MX1	-1.58441	1.88E-05
TMEM106B	-1.58358	1.78E-05
C19orf28	-1.57808	2.52E-05
LYPLA2	-1.57661	2.56E-06
RYK	-1.57635	2.50E-05
DSG2	-1.57567	1.01E-06
ITFG2	-1.57517	3.68E-05
GYG2	-1.57342	3.99E-05
FXYD5	-1.57254	5.90E-05
EXTL1	-1.57247	5.22E-05
MITF	-1.56986	1.28E-05
PPRC1	-1.56975	1.36E-06
NPTX1	-1.56951	1.39E-03
CAMK2D	-1.56762	3.45E-05
FAF2	-1.56677	6.39E-05
GIT1	-1.56667	1.19E-04
SLC6A15	-1.56622	9.16E-08
TXLNA	-1.56619	3.90E-04
SP1	-1.56604	3.30E-04
TMBIM6	-1.56534	2.74E-07
CKS2	-1.56515	1.06E-05
KIAA1147	-1.56489	6.00E-05
ECE2	-1.56244	2.01E-06
ICK	-1.56239	7.76E-07
SARS2	-1.56137	5.03E-06
ADAT1	-1.56119	8.81E-05
AP1M1	-1.56077	2.73E-04
CADPS2	-1.56011	8.07E-08
FHDC1	-1.55982	7.99E-06

CDK5R1	-1.55972	6.21E-07
RHOQ	-1.55972	5.77E-05
MAFG	-1.55899	1.76E-06
PRRG1	-1.55888	1.24E-06
BIRC7	-1.55721	1.48E-05
METTL21B	-1.55616	8.00E-06
DBNDD1	-1.55546	3.14E-05
C9orf23	-1.55447	4.86E-06
FAM83D	-1.55419	4.57E-06
LINGO1	-1.55406	6.58E-08
TMUB2	-1.55324	1.91E-06
CPNE8	-1.55292	2.41E-05
CLIC4	-1.55265	1.57E-04
TGFA	-1.55265	2.42E-05
USP46	-1.55211	1.83E-07
CORO1C	-1.55206	1.01E-06
MFSD3	-1.55162	4.54E-04
RNF20	-1.55134	1.56E-05
KIAA1737	-1.55031	6.85E-05
C15orf57	-1.54993	4.63E-05
CDK2	-1.5497	4.99E-04
C19orf28	-1.54896	3.88E-05
AGAP3	-1.5488	1.01E-06
HSPBP1	-1.5487	1.15E-05
CADM1	-1.54842	9.63E-06
NCLN	-1.54799	1.96E-03
ZMAT5	-1.54658	3.12E-04
MAP6D1	-1.54473	2.06E-05
NRGN	-1.5423	7.44E-04
EFHD2	-1.54168	6.51E-06
HCP5	-1.54104	1.92E-05
RFC5	-1.54009	1.97E-05
ADCK2	-1.53879	5.94E-05
CMTM4	-1.53874	3.20E-05
RYK	-1.53801	5.05E-04
KLHL21	-1.5373	1.73E-04
GRWD1	-1.53636	8.36E-05
SLC6A8	-1.53605	1.38E-04
PPARGC1A	-1.53507	1.64E-06
ZDHHC16	-1.53468	4.38E-04
MIF4GD	-1.53431	3.51E-04
SLC35B2	-1.5339	4.07E-04
PACS1	-1.53268	2.73E-04
SMO	-1.53264	1.57E-06

CAMKK2	-1.53197	2.84E-06
ISY1	-1.53136	3.14E-05
SETDB1	-1.53015	1.52E-04
URB2	-1.52954	1.20E-05
EI24	-1.52823	2.40E-04
WIPF1	-1.52753	7.57E-06
DPYSL3	-1.52749	1.54E-05
ABR	-1.52705	5.99E-05
PPP2R4	-1.52579	2.26E-05
RSBN1	-1.52334	9.28E-06
LRRC8A	-1.5219	1.45E-05
ST3GAL5	-1.52138	4.29E-04
SPOCD1	-1.52091	2.87E-06
RAB38	-1.52065	1.92E-04
AGFG1	-1.5202	1.23E-06
RFFL	-1.51979	4.43E-05
VOPP1	-1.51958	5.31E-04
SLC16A6	-1.51877	3.24E-05
COBRA1	-1.51862	2.55E-03
C17orf63	-1.51771	1.24E-04
OSBPL3	-1.51767	1.94E-06
ECE2	-1.51746	1.92E-06
UBR7	-1.51616	1.79E-05
PPP1R3C	-1.51612	8.83E-05
ALKBH2	-1.51569	2.62E-04
XYLT1	-1.51491	2.17E-06
NMI	-1.51483	2.25E-03
WFS1	-1.51376	1.14E-04
LAD1	-1.51372	1.86E-06
CASP2	-1.51296	2.86E-04
TM9SF1	-1.51259	2.19E-05
TMEM214	-1.51228	2.37E-04
WDR91	-1.51187	8.41E-04
PKMYT1	-1.5115	9.87E-04
TMEM180	-1.50994	3.76E-05
SKP2	-1.50949	3.76E-06
IRS2	-1.50889	2.69E-05
EML3	-1.50824	5.57E-06
CKS2	-1.50742	6.11E-05
OST4	-1.50668	1.59E-05
SGK223	-1.50609	4.30E-05
FOXO3	-1.50466	4.91E-07
ARF4	-1.50402	1.18E-05
DCTD	-1.50388	5.40E-06

EXD2	-1.50366	3.63E-06
UBE2E2	-1.50118	2.89E-05
CPNE8	-1.50117	5.48E-05
PLEKHA2	-1.50087	7.09E-05
SLC7A1	-1.5008	1.33E-05
B4GALT7	-1.50062	1.25E-05
