# Supplementary material for: microRNA-7-5p inhibits melanoma cell proliferation and metastasis by suppressing RelA/NF-κB
Source: Oncotarget. 2016 May 17;7(22):31663–80. doi: 10.18632/oncotarget.9421 (PMC5077967; doi:10.18632/oncotarget.9421)
Supplement: Supplementary file 4 [file oncotarget-07-31663-s004.pdf]

Supplementary Table S3: Genes downregulated in the microarray that contain a miR-7-5p seed sequence.

Gene Symbol	Fold Change	Confidence of miR-7 seed region	Pathway
ACSL4	-1.812	High (predicted)	Fatty Acid Activation Fatty Acid β-oxidation I LPS/IL-1 Mediated Inhibition of RXR Function Mitochondrial L-carnitine Shuttle Pathway Stearate Biosynthesis I (Animals) Type II Diabetes Mellitus Signaling γ-linolenate Biosynthesis II (Animals)
ADCY9	-1.678	High (predicted)	Breast Cancer Regulation by Stathmin1 CDK5 Signaling CREB Signaling in Neurons CXCR4 Signaling Cardiac Hypertrophy Signaling Cardiac β-adrenergic Signaling Cellular Effects of Sildenafil (Viagra) Colorectal Cancer Metastasis Signaling Corticotropin Releasing Hormone Signaling Dopamine Receptor Signaling Dopamine-DARPP32 Feedback in cAMP Signaling Endothelin-1 Signaling G-Protein Coupled Receptor Signaling GNRH Signaling Gap Junction Signaling Gαi Signaling Gαs Signaling Hepatic Cholestasis IL-1 Signaling Leptin Signaling in Obesity Melanocyte Development and Pigmentation Signaling Molecular Mechanisms of Cancer P2Y Purigenic Receptor Signaling Pathway PPARα/RXRα Activation Phospholipase C Signaling Protein Kinase A Signaling RAR Activation Relaxin Signaling Renin-Angiotensin Signaling Role of NFAT in Cardiac Hypertrophy Sphingosine-1-phosphate Signaling Thrombin Signaling cAMP-mediated signaling eNOS Signaling α-Adrenergic Signaling
ANKFY1	-1.605	High (predicted)	Macropinocytosis Signaling
ARF4	-1.897	Moderate (predicted)	Integrin Signaling
ARMC10	-2.902	Moderate (predicted)	
ATG7	-1.615	High (predicted)	
BCL2L12	-3.544	Moderate (predicted)	
CNO	-1.934	High (predicted)	
C10orf57	-1.871	High (predicted)	

C12orf49	-1.797	Moderate (predicted)	
C1orf21	-1.794	High (predicted)	
C20orf24	-2.267	High (predicted)	
CALM3	-2.803	Moderate (predicted)	Androgen Signaling B Cell Receptor Signaling Breast Cancer Regulation by Stathmin1 CCR3 Signaling in Eosinophils CCR5 Signaling in Macrophages CD28 Signaling in T Helper Cells CREB Signaling in Neurons Calcium Signaling Calcium-induced T Lymphocyte Apoptosis Cardiac Hypertrophy Signaling Cellular Effects of Sildenafil (Viagra) Chemokine Signaling Corticotropin Releasing Hormone Signaling Dopamine-DARPP32 Feedback in cAMP Signaling Glioma Signaling Glutamate Receptor Signaling Gαq Signaling Melatonin Signaling Nitric Oxide Signaling in the Cardiovascular System Nur77 Signaling in T Lymphocytes PI3K Signaling in B Lymphocytes Phospholipase C Signaling Protein Kinase A Signaling RANK Signaling in Osteoclasts Regulation of IL-2 Expression in Activated and Anergic T Lymphocytes Role of Macrophages, Fibroblasts and Endothelial Cells in Rheumatoid Arthritis Role of NFAT in Cardiac Hypertrophy Role of NFAT in Regulation of the Immune Response Role of Osteoblasts, Osteoclasts and Chondrocytes in Rheumatoid Arthritis Synaptic Long Term Potentiation T Cell Receptor Signaling cAMP-mediated signaling eNOS Signaling fMLP Signaling in Neutrophils iCOS-iCOSL Signaling in T Helper Cells iNOS Signaling nNOS Signaling in Neurons nNOS Signaling in Skeletal Muscle Cells α-Adrenergic Signaling
CALU	-1.670	High (predicted)	
CAMK2D	-1.623	High (predicted)	B Cell Receptor Signaling Breast Cancer Regulation by Stathmin1 CREB Signaling in Neurons Calcium Signaling Chemokine Signaling Crosstalk between Dendritic Cells and Natural Killer Cells G-Protein Coupled Receptor Signaling GM-CSF Signaling GNRH Signaling Glioma Signaling Melatonin Signaling Molecular Mechanisms of Cancer Neuropathic Pain Signaling In Dorsal Horn Neurons PI3K Signaling in B Lymphocytes PKCθ Signaling in T Lymphocytes Protein Kinase A Signaling Role of Macrophages, Fibroblasts and Endothelial Cells in Rheumatoid Arthritis Role of NFAT in Cardiac Hypertrophy Synaptic Long Term Potentiation Thrombin Signaling Xenobiotic Metabolism Signaling cAMP-mediated signaling iCOS-iCOSL Signaling in T Helper Cells
CAMKK2	-1.532	Moderate (predicted)	Calcium Signaling Dopamine-DARPP32 Feedback in cAMP Signaling

CAPZA1	-1.630	Moderate (predicted)	
CAV1	-2.063	Moderate (predicted)	Caveolar-mediated Endocytosis Signaling G Beta Gamma Signaling Gap Junction Signaling Gαi Signaling Integrin Signaling Nitric Oxide Signaling in the Cardiovascular System PDGF Signaling Virus Entry via Endocytic Pathways eNOS Signaling
CISD2	-1.881	High (predicted)	
CKAP4	-4.226	High (predicted)	
CLIC4	-1.553	Moderate (predicted)	
CNN3	-2.865	High (predicted)	
CNOT8	-1.622	High (predicted)	
COL1A2	-1.974	High (predicted)	Atherosclerosis Signaling Dendritic Cell Maturation Hepatic Fibrosis / Hepatic Stellate Cell Activation Intrinsic Prothrombin Activation Pathway
COMMD7	-2.270	Moderate (predicted)	
CRTAP	-6.729	Moderate (predicted)	
CTDSP2	-2.457	High (predicted)	
CTDSPL	-2.328	High (predicted)	
CTSK	-6.274	High (predicted)	Role of Osteoblasts, Osteoclasts and Chondrocytes in Rheumatoid Arthritis
DAZAP2	-3.338	High (predicted)	
DBNL	-1.588	Moderate (predicted)	
DNAJC15	-2.049	High (predicted)	Aldosterone Signaling in Epithelial Cells NRF2-mediated Oxidative Stress Response Protein Ubiquitination Pathway
DPYSL2	-1.620	High (predicted)	Axonal Guidance Signaling Semaphorin Signaling in Neurons Thymine Degradation Uracil Degradation II (Reductive)
EGR3	-1.727	High (predicted)	

EHD1	-2.799	High (predicted)	
EIF2AK1	-2.366	High (predicted)	EIF2 Signaling
ENO2	-1.919	Moderate (predicted)	Gluconeogenesis I Glycolysis I
EXOSC2	-1.660	High (predicted)	
EXTL3	-1.783	High (predicted)	Heparan Sulfate Biosynthesis Heparan Sulfate Biosynthesis (Late Stages)
FAM46C	-2.812	High (predicted)	
FGF13	-1.656	Moderate (predicted)	Actin Cytoskeleton Signaling Bladder Cancer Signaling Clathrin-mediated Endocytosis Signaling FGF Signaling
GLO1	-3.508	Moderate (predicted)	Methylglyoxal Degradation I
GLS	-2.411	Moderate (predicted)	Citrulline Biosynthesis Glutamate Receptor Signaling Glutamine Degradation I Huntington's Disease Signaling Superpathway of Citrulline Metabolism
HECTD3	-1.717	High (predicted)	
HIATL1	-2.611	Moderate (predicted)	
HMGN4	-2.098	High (predicted)	
IDE	-2.033	High (predicted)	Neuroprotective Role of THOP1 in Alzheimer's Disease
IGSF3	-1.842	Moderate (predicted)	
IRS2	-1.509	Experimentally Observed,High (predicted)	AMPK Signaling GDNF Family Ligand-Receptor Interactions IGF-1 Signaling IL-9 Signaling ILK Signaling Insulin Receptor Signaling PI3K Signaling in B Lymphocytes Role of JAK1 and JAK3 in γc Cytokine Signaling Role of JAK2 in Hormone-like Cytokine Signaling Thrombopoietin Signaling Type II Diabetes Mellitus Signaling
ISY1	-1.531	Moderate (predicted)	
ITFG2	-1.575	Moderate (predicted)	

KDM5B	-1.585	Moderate (predicted)	Role of Oct4 in Mammalian Embryonic Stem Cell Pluripotency
KIAA0247	-1.604	High (predicted)	
KPNA6	-2.407	High (predicted)	RAN Signaling
MAFG	-1.617	High (predicted)	NRF2-mediated Oxidative Stress Response
MAU2	-1.611	High (predicted)	
MAZ	-1.685	High (predicted)	
MMACHC	-1.853	Moderate (predicted)	
MRAP2	-1.669	Moderate (predicted)	
NDFIP2	-1.950	High (predicted)	
NDUFA4	-2.690	High (predicted)	Mitochondrial Dysfunction
NR1H2	-2.648	High (predicted)	LPS/IL-1 Mediated Inhibition of RXR Function LXR/RXR Activation
NUDCD3	-2.373	High (predicted)	
OSBPL3	-1.518	High (predicted)	
OST4	-1.507	High (predicted)	
PAK1	-1.631	Experimentally Observed,Moderate (predicted)	Actin Cytoskeleton Signaling Agrin Interactions at Neuromuscular Junction Amyotrophic Lateral Sclerosis Signaling Angiopoietin Signaling Axonal Guidance Signaling Breast Cancer Regulation by Stathmin1 CCR3 Signaling in Eosinophils CD28 Signaling in T Helper Cells CXCR4 Signaling Cdc42 Signaling ERK/MAPK Signaling Ephrin A Signaling Ephrin B Signaling Ephrin Receptor Signaling ErbB Signaling FAK Signaling Fcγ Receptor-mediated Phagocytosis in Macrophages and Monocytes G Beta Gamma Signaling GNRH Signaling Germ Cell-Sertoli Cell Junction Signaling HGF Signaling IL-3 Signaling Integrin Signaling LPS-stimulated MAPK Signaling Macropinocytosis Signaling Molecular Mechanisms of Cancer Natural Killer Cell Signaling PAK Signaling Paxillin Signaling Pyridoxal 5'-phosphate Salvage Pathway Rac Signaling Regulation of Actin-based Motility by Rho Renal Cell Carcinoma Signaling Renin-Angiotensin Signaling RhoGDI Signaling Role of Tissue Factor in Cancer Salvage Pathways of Pyrimidine

			Ribonucleotides Semaphorin Signaling in Neurons Signaling by Rho Family GTPases TNFR1 Signaling
PAK2	-2.036	Moderate (predicted)	Actin Cytoskeleton Signaling Agrin Interactions at Neuromuscular Junction Angiopoietin Signaling Axonal Guidance Signaling CCR3 Signaling in Eosinophils CXCR4 Signaling Cdc42 Signaling ERK/MAPK Signaling Ephrin Receptor Signaling ErbB Signaling FAK Signaling GNRH Signaling Germ Cell-Sertoli Cell Junction Signaling IL-8 Signaling Integrin Signaling Molecular Mechanisms of Cancer Natural Killer Cell Signaling PAK Signaling Paxillin Signaling Pyridoxal 5'-phosphate Salvage Pathway Rac Signaling Regulation of Actin-based Motility by Rho Renal Cell Carcinoma Signaling Renin-Angiotensin Signaling RhoGDI Signaling Salvage Pathways of Pyrimidine Ribonucleotides Semaphorin Signaling in Neurons Signaling by Rho Family GTPases TNFR1 Signaling
PATL1	-2.337	High (predicted)	
PDXDC1	-2.168	High (predicted)	
PFN2	-2.079	High (predicted)	Actin Cytoskeleton Signaling Axonal Guidance Signaling Regulation of Actin-based Motility by Rho RhoA Signaling
PHACTR4	-1.595	High (predicted)	
PHF16	-2.089	Moderate (predicted)	
PIGH	-2.844	High (predicted)	
PLEC	-1.989	High (predicted)	
PLXNA1	-1.940	High (predicted)	Axonal Guidance Signaling RhoA Signaling Semaphorin Signaling in Neurons
POLE4	-6.861	High (predicted)	
PRMT2	-2.310	High (predicted)	RAR Activation
PSME3	-5.862	High (predicted)	Polyamine Regulation in Colon Cancer
PURB	-1.803	High (predicted)	
RAB11FIP5	-1.616	High (predicted)	
RAF1	-2.594	Experimentally Observed,High	14-3-3-mediated Signaling Actin Cytoskeleton Signaling Acute Myeloid Leukemia Signaling Acute Phase Response Signaling Aldosterone Signaling in Epithelial Cells

		(predicted)	Apoptosis Signaling Axonal Guidance Signaling B Cell Receptor Signaling BMP signaling pathway Bladder Cancer Signaling Breast Cancer Regulation by Stathmin1 CCR3 Signaling in Eosinophils CDK5 Signaling CNTF Signaling CREB Signaling in Neurons CXCR4 Signaling Cardiac Hypertrophy Signaling Cdc42 Signaling Ceramide Signaling Chemokine Signaling Cholecystokinin/Gastrin-mediated Signaling Chronic Myeloid Leukemia Signaling Corticotropin Releasing Hormone Signaling Cyclins and Cell Cycle Regulation EGF Signaling EIF2 Signaling ERK/MAPK Signaling Endometrial Cancer Signaling Endothelin-1 Signaling Ephrin Receptor Signaling ErbB Signaling ErbB2-ErbB3 Signaling ErbB4 Signaling Erythropoietin Signaling Estrogen Receptor Signaling FAK Signaling FGF Signaling FLT3 Signaling in Hematopoietic Progenitor Cells Fc Epsilon RI Signaling G Beta Gamma Signaling G-Protein Coupled Receptor Signaling GDNF Family Ligand-Receptor Interactions GM-CSF Signaling GNRH Signaling Gap Junction Signaling Glioblastoma Multiforme Signaling Glioma Signaling Glucocorticoid Receptor Signaling Gα12/13 Signaling Gαi Signaling Gαq Signaling HGF Signaling IGF-1 Signaling IL-15 Signaling IL-2 Signaling IL-3 Signaling IL-6 Signaling IL-8 Signaling Insulin Receptor Signaling Integrin Signaling JAK/Stat Signaling LPS-stimulated MAPK Signaling Melanocyte Development and Pigmentation Signaling Melanoma Signaling Melatonin Signaling Molecular Mechanisms of Cancer Mouse Embryonic Stem Cell Pluripotency NF-κB Activation by Viruses NGF Signaling NRF2-mediated Oxidative Stress Response Natural Killer Cell Signaling Neuregulin Signaling Neurotrophin/TRK Signaling Non-Small Cell Lung Cancer Signaling Oncostatin M Signaling Ovarian Cancer Signaling P2Y Purigenic Receptor Signaling Pathway PAK Signaling PDGF Signaling PEDF Signaling PI3K Signaling in B Lymphocytes PI3K/AKT Signaling PPAR Signaling PPARα/RXRα Activation PTEN Signaling Pancreatic Adenocarcinoma Signaling Phospholipase C Signaling Prolactin Signaling Prostate Cancer Signaling Protein Kinase A Signaling RANK Signaling in Osteoclasts Rac Signaling Regulation of IL-2 Expression in Activated and Anergic T Lymphocytes Regulation of eIF4 and p70S6K Signaling Renal Cell Carcinoma Signaling Renin-Angiotensin Signaling Role of IL-17F in Allergic Inflammatory Airway Diseases Role of JAK1, JAK2 and TYK2 in Interferon Signaling Role of MAPK Signaling in the Pathogenesis of Influenza Role of Macrophages, Fibroblasts and Endothelial Cells in Rheumatoid Arthritis Role of NANOG in Mammalian Embryonic Stem Cell Pluripotency Role of NFAT in Cardiac Hypertrophy Role of NFAT in Regulation of the Immune Response Sertoli Cell-Sertoli Cell Junction Signaling Signaling by Rho Family GTPases Synaptic Long Term Depression Synaptic Long Term Potentiation T Cell Receptor

			Signaling TGF-β Signaling Telomerase Signaling Thrombin Signaling Thrombopoietin Signaling VEGF Family Ligand-Receptor Interactions VEGF Signaling Xenobiotic Metabolism Signaling cAMP-mediated signaling fMLP Signaling in Neutrophils p70S6K Signaling α-Adrenergic Signaling
RCC2	-3.057	High (predicted)	
RELA	-1.588	High (predicted)	4-1BB Signaling in T Lymphocytes Activation of IRF by Cytosolic Pattern Recognition Receptors Acute Myeloid Leukemia Signaling Acute Phase Response Signaling Altered T Cell and B Cell Signaling in Rheumatoid Arthritis Androgen Signaling Angiopoietin Signaling Antioxidant Action of Vitamin C Apoptosis Signaling April Mediated Signaling Aryl Hydrocarbon Receptor Signaling Atherosclerosis Signaling B Cell Activating Factor Signaling B Cell Receptor Signaling BMP signaling pathway CD27 Signaling in Lymphocytes CD28 Signaling in T Helper Cells CD40 Signaling Ceramide Signaling Chronic Myeloid Leukemia Signaling Colorectal Cancer Metastasis Signaling Crosstalk between Dendritic Cells and Natural Killer Cells Death Receptor Signaling Dendritic Cell Maturation Erythropoietin Signaling Estrogen-Dependent Breast Cancer Signaling G-Protein Coupled Receptor Signaling GNRH Signaling Glucocorticoid Receptor Signaling Gα12/13 Signaling Gαq Signaling HMGB1 Signaling Hepatic Cholestasis Hepatic Fibrosis / Hepatic Stellate Cell Activation IL-1 Signaling IL-10 Signaling IL-12 Signaling and Production in Macrophages IL-15 Production IL-15 Signaling IL-17 Signaling IL-17A Signaling in Airway Cells IL-17A Signaling in Fibroblasts IL-17A Signaling in Gastric Cells IL-6 Signaling IL-8 Signaling IL-9 Signaling ILK Signaling Induction of Apoptosis by HIV1 Interferon Signaling LPS-stimulated MAPK Signaling LXR/RXR Activation Lymphotoxin β Receptor Signaling MIF Regulation of Innate Immunity MIF-mediated Glucocorticoid Regulation Molecular Mechanisms of Cancer NF-κB Activation by Viruses NF-κB Signaling NGF Signaling OX40 Signaling Pathway P2Y Purigenic Receptor Signaling Pathway PEDF Signaling PI3K Signaling in B Lymphocytes PI3K/AKT Signaling PKCθ Signaling in T Lymphocytes PPAR Signaling PPARα/RXRα Activation PTEN Signaling PXR/RXR Activation Pancreatic Adenocarcinoma Signaling Phospholipase C Signaling Production of Nitric Oxide and Reactive Oxygen Species in Macrophages Prostate Cancer Signaling Protein Kinase A Signaling RANK Signaling in Osteoclasts RAR Activation Rac Signaling Regulation of IL-2 Expression in Activated and Anergic T Lymphocytes Relaxin Signaling Renin-Angiotensin Signaling Role of IL-17A in Arthritis Role of IL-17F in Allergic Inflammatory Airway Diseases Role of JAK1, JAK2 and TYK2 in Interferon Signaling Role of Macrophages, Fibroblasts and

			Endothelial Cells in Rheumatoid Arthritis Role of NFAT in Regulation of the Immune Response Role of Osteoblasts, Osteoclasts and Chondrocytes in Rheumatoid Arthritis Role of PI3K/AKT Signaling in the Pathogenesis of Influenza Role of PKR in Interferon Induction and Antiviral Response Role of Pattern Recognition Receptors in Recognition of Bacteria and Viruses Role of RIG1-like Receptors in Antiviral Innate Immunity Signaling by Rho Family GTPases Small Cell Lung Cancer Signaling T Cell Receptor Signaling TNFR1 Signaling TNFR2 Signaling TREM1 Signaling TWEAK Signaling Thrombin Signaling Tight Junction Signaling Toll-like Receptor Signaling Type I Diabetes Mellitus Signaling Type II Diabetes Mellitus Signaling Xenobiotic Metabolism Signaling fMLP Signaling in Neutrophils iCOS-iCOSL Signaling in T Helper Cells iNOS Signaling
RFFL	-1.520	Moderate (predicted)	
RNF144A	-2.581	High (predicted)	
RNF20	-1.551	High (predicted)	
RPL15	-2.029	Moderate (predicted)	EIF2 Signaling
RSBN1	-1.523	High (predicted)	
RUSC1	-3.261	Moderate (predicted)	
RYK	-1.612	High (predicted)	Role of Macrophages, Fibroblasts and Endothelial Cells in Rheumatoid Arthritis
SCARB2	-3.528	Moderate (predicted)	
SDHC	-1.845	Moderate (predicted)	Mitochondrial Dysfunction TCA Cycle II (Eukaryotic)
SEPT2	-1.822	Moderate (predicted)	RhoA Signaling Signaling by Rho Family GTPases
SEPW1	-2.584	Moderate (predicted)	
SERP1	-1.589	High (predicted)	

SETD8	-1.716	High (predicted)	
SIGMAR1	-2.718	Moderate (predicted)	
SKP1	-2.755	High (predicted)	Antiproliferative Role of TOB in T Cell Signaling Cell Cycle: G1/S Checkpoint Regulation Cell Cycle: G2/M DNA Damage Checkpoint Regulation Cyclins and Cell Cycle Regulation Protein Ubiquitination Pathway
SLC25A15	-2.652	High (predicted)	
SLC2A10	-2.033	Moderate (predicted)	
SLC35A5	-1.901	Moderate (predicted)	
SMARCD1	-3.597	High (predicted)	
SMO	-1.533	Moderate (predicted)	Axonal Guidance Signaling Basal Cell Carcinoma Signaling Colorectal Cancer Metastasis Signaling Corticotropin Releasing Hormone Signaling Factors Promoting Cardiogenesis in Vertebrates Glioblastoma Multiforme Signaling Human Embryonic Stem Cell Pluripotency Molecular Mechanisms of Cancer Mouse Embryonic Stem Cell Pluripotency Ovarian Cancer Signaling Protein Kinase A Signaling Role of Macrophages, Fibroblasts and Endothelial Cells in Rheumatoid Arthritis Role of NANOG in Mammalian Embryonic Stem Cell Pluripotency Role of Osteoblasts, Osteoclasts and Chondrocytes in Rheumatoid Arthritis Role of Wnt/GSK-3β Signaling in the Pathogenesis of Influenza Sonic Hedgehog Signaling Wnt/β-catenin Signaling
SNAP29	-2.284	Moderate (predicted)	
SNCA	-1.927	Experimentally Observed,High (predicted)	14-3-3-mediated Signaling Huntington's Disease Signaling Mitochondrial Dysfunction Parkinson's Signaling
SORT1	-1.593	High (predicted)	
SP1	-1.566	High (predicted)	Aryl Hydrocarbon Receptor Signaling ErbB2-ErbB3 Signaling Estrogen-Dependent Breast Cancer Signaling Gap Junction Signaling HMGB1 Signaling Huntington's Disease Signaling IL-10 Signaling Prolactin Signaling Telomerase Signaling VDR/RXR Activation

SPTY2D1	-1.591	High (predicted)	
SRGAP2	-1.662	High (predicted)	Axonal Guidance Signaling
ST8SIA5	-1.615	Moderate (predicted)	
STMN3	-1.928	Moderate (predicted)	
TBC1D2B	-1.888	Moderate (predicted)	
TCF12	-1.841	High (predicted)	PEDF Signaling
TGFA	-1.553	Moderate (predicted)	ErbB Signaling Hepatic Fibrosis / Hepatic Stellate Cell Activation NF-κB Signaling Neuregulin Signaling Non-Small Cell Lung Cancer Signaling Pancreatic Adenocarcinoma Signaling Renal Cell Carcinoma Signaling
TMED9	-2.911	High (predicted)	
TMEM43	-3.682	Moderate (predicted)	
TMEM69	-2.069	Moderate (predicted)	
TMEM97	-3.435	Moderate (predicted)	
C18orf10	-3.952	Moderate (predicted)	
UBE2D4	-2.132	Moderate (predicted)	Hypoxia Signaling in the Cardiovascular System Protein Ubiquitination Pathway
UBE2J1	-1.980	Moderate (predicted)	Hypoxia Signaling in the Cardiovascular System Protein Ubiquitination Pathway
UBQLN4	-3.029	High (predicted)	
UBXN2B	-1.857	High (predicted)	
UHRF1	-2.029	High (predicted)	

VDAC1	-1.798	High (predicted)	
VPS26A	-3.110	High (predicted)	
ZDHHC9	-2.995	High (predicted)	
ZMIZ1	-2.428	High (predicted)	
ZNF275	-1.667	Moderate (predicted)	
