# Supplementary material for: microRNA-7-5p inhibits melanoma cell proliferation and metastasis by suppressing RelA/NF-κB
Source: Oncotarget. 2016 May 17;7(22):31663–80. doi: 10.18632/oncotarget.9421 (PMC5077967; doi:10.18632/oncotarget.9421)
Supplement: Supplementary file 5 [file oncotarget-07-31663-s005.pdf]

Supplementary Table S4. Functional pathways enriched for genes downregulated with miR-7-5p in WM266-4 cells.

Category	Function	Function Annotation	p-value	Molecules	#
Neurological Disease	disorder of basal ganglia	disorder of basal ganglia	8.98E-05	CAMKK2, CASP2, CDK5R1, DBNL, DHCR7, DPYSL3, EMX2, ENO2, ETV4, FGF13, GIT1, HSPA5, ITPKB, MICALL1, MT1X, MT2A, NOTCH2, NRGN, PAK1, PFN2, PNKD, PPARGC1A, PPRC1, RELA, RGS4, RNF114, RNF5, RPL15, SCARB2, SDC4, SETDB1, SH3BP4, SLC20A2, SNCA, SORT1, SRM, STOM, TGM2, TMED10, TRAK2, UBE2D4	41
Neurological Disease	movement disorder	Movement Disorders	6.32E-04	CAMKK2, CASP2, CDK5R1, DBNL, DHCR7, DPYSL3, EMX2, ENO2, ETV4, FGF13, GIT1, HSPA5, ITPKB, LINGO1, MICALL1, MT1X, MT2A, NOTCH2, NRGN, PAK1, PFN2, PPARGC1A, PPRC1, RELA, RGS4, RNF114, RNF5, RPL15, SCARB2, SDC4, SETDB1, SH3BP4, SIGMAR1, SNCA, SORT1, SRM, STOM, TGM2, TMED10, TRAK2, UBE2D4	41
Neurological Disease	Wolfram Syndrome	Wolfram Syndrome	7.65E-04	CISD2, WFS1	2
Neurological Disease	Huntington's disease	Huntington's Disease	1.31E-03	CAMKK2, CASP2, CDK5R1, DHCR7, DPYSL3, EMX2, ETV4, FGF13, GIT1, HSPA5, ITPKB, MICALL1, MT1X, MT2A, NOTCH2, NRGN, PFN2, PPARGC1A, PPRC1, RGS4, SCARB2, SDC4, SETDB1, SH3BP4, SORT1, SRM, STOM, TGM2, TRAK2, UBE2D4	3 0
Neurological Disease	neuromuscular disease	neuromuscular disease	2.14E-03	CAMKK2, CASP2, CDK5R1, DBNL, DHCR7, DPYSL3, EMX2, ENO2, ETV4, FGF13, GIT1, HSPA5, ITPKB, MICALL1, MT1X, MT2A, MX1, NOTCH2, NRGN, PAK1, PFN2, PPARGC1A, PPRC1, RELA, RGS4, RNF114, RNF5, RPL15, SCARB2, SDC4, SETDB1, SH3BP4, SNCA, SORT1, SRM, STOM, TGM2, TMED10, TRAK2, UBE2D4	40
Neurological Disease	Krabbe's disease	Krabbe's disease	4.42E-03	CAV1, FLOT2	2
Neurological	hydrocephalus	hydrocephalus	1.46E-02	ENO2, L1CAM	2

Disease					
Neurological Disease	encephalopathy	autosomal dominant encephalopathy	1.92E-02	PSEN2, SNCA	2
Neurological Disease	Alzheimer's disease type 4	Alzheimer's disease type 4	2.77E-02	PSEN2	1
Neurological Disease	Amish lethal microcephaly	Amish lethal microcephaly	2.77E-02	SLC25A19	1
Neurological Disease	CRASH syndrome	CRASH syndrome	2.77E-02	L1CAM	1
Neurological Disease	Charcot-Marie-Tooth disease axonal type 1c	Charcot-Marie-Tooth disease axonal type 1c	2.77E-02	LITAF	1
Neurological Disease	Parkinson disease 1	Parkinson disease 1	2.77E-02	SNCA	1
Neurological Disease	X-linked hydrocephalus with congenital idiopathic intestinal pseudoobstruction	X-linked hydrocephalus with congenital idiopathic intestinal pseudoobstruction	2.77E-02	L1CAM	1
Neurological Disease	amish infantile epilepsy syndrome	amish infantile epilepsy syndrome	2.77E-02	ST3GAL5	1
Neurological Disease	autosomal dominant Parkinson disease 4	autosomal dominant Parkinson disease 4	2.77E-02	SNCA	1
Neurological Disease	bilateral frontoparietal polymicrogyria	bilateral frontoparietal polymicrogyria	2.77E-02	GPR56	1
Neurological Disease	cerebral dysgenesis, neuropathy, ichthyosis, and palmoplantar keratoderma syndrome, cednik syndrome	cerebral dysgenesis, neuropathy, ichthyosis, and palmoplantar keratoderma syndrome, cednik syndrome	2.77E-02	SNAP29	1
Neurological Disease	creatine deficiency syndrome	creatine deficiency syndrome	2.77E-02	SLC6A8	1
Neurological Disease	idiopathic basal ganglia calcification type 3	idiopathic basal ganglia calcification type 3	2.77E-02	SLC20A2	1
Neurological Disease	juvenile amyotrophic lateral sclerosis type 16	juvenile amyotrophic lateral sclerosis type 16	2.77E-02	SIGMAR1	1

Neurological Disease	mucopolysaccharidosis type VI	mucopolysaccharidosis type VI	2.77E-02	ARSB	1
Neurological Disease	nonsyndromic low frequency sensorineural hearing loss	nonsyndromic low frequency sensorineural hearing loss	2.77E-02	WFS1	1
Neurological Disease	paroxysmal choreoathetosis	paroxysmal choreoathetosis	2.77E-02	PNKD	1
Neurological Disease	partial agenesis of corpus callosum	partial agenesis of corpus callosum	2.77E-02	L1CAM	1
Cellular Movement	migration	migration of adenocarcinoma cell lines	7.65E-04	TGFA, TGM2	2
Cellular Movement	migration	migration of lung cancer cell lines	4.00E-03	CAV1, ETV4, PTK2, STMN3, TGFA, TGM2	6
Cellular Movement	migration	migration of tumor cell lines	9.62E-03	ACSL4, AGK, ANKS1A, CAV1, CMTM8, DPYSL2, ELK1, ETV4, FOXO3, GAB2, GIT1, L1CAM, MITF, NOTCH2, PAK1, PAK2, PLXNA1, PTK2, RNF144A, SIRPA, ST6GAL1, STMN3, TCF12, TGFA, TGM2, WBP2	26
Cellular Movement	migration	migration of breast cancer cell lines	1.04E-02	ACSL4, ANKS1A, FOXO3, GAB2, PAK1, PAK2, PLXNA1, PTK2, RNF144A, TGFA, WBP2	11
Cellular Movement	migration	migration of hepatoma cell lines	3.06E-02	CAV1, CMTM8, ELK1, PAK1	4
Cellular Movement	migration	migration of carcinoma cells	3.57E-02	GNA12, PAK1	2
Cellular Movement	migration	migration of vascular smooth muscle cells	4.93E-02	CLIC4, GIT1, SDC4	3
Cellular Movement	cell movement	cell movement of tumor cells	1.42E-03	CAV1, CBL, GNA12, L1CAM, PAK1, PTK2, SIRPA	7
Cellular Movement	cell movement	cell movement of carcinoma cells	4.90E-03	GNA12, L1CAM, PAK1	3
Cellular Movement	cell movement	cell movement of cancer cells	1.42E-02	CAV1, GNA12, L1CAM, PAK1, SIRPA	5
Cellular Movement	cell movement	cell movement of breast cancer cell lines	1.52E-02	ACSL4, ANKS1A, FOXO3, GAB2, L1CAM, PAK1, PAK2, PLXNA1, PTK2, RNF144A, TGFA, WBP2	12
Cellular Movement	cell movement	cell movement of vascular smooth muscle cells	1.54E-02	CLIC4, GIT1, SDC4, TRIB1	4
Cellular	cell movement	cell movement of colon carcinoma cells	2.77E-02	L1CAM	1

Movement					
Cellular Movement	cell movement	cell movement of melanoma cell lines	4.21E-02	FOXO3, L1CAM, MITF, MX1, PAK1	5
Cellular Movement	cell movement	cell movement of tumor cell lines	4.34E-02	ACSL4, AGK, ANKS1A, CAV1, CMTM8, DPYSL2, ELK1, ETV4, FOXO3, GAB2, GIT1, L1CAM, MITF, MX1, NOTCH2, PAK1, PAK2, PLXNA1, PTK2, RNF144A, SIRPA, ST3GAL5, ST6GAL1, STMN3, TCF12, TGFA, TGM2, WBP2	28
Cellular Movement	invasion	invasion of tumor cell lines	2.94E-03	ACSL4, CAV1, CBL, CDK5R1, CMTM8, CTSK, ELK1, ETV4, FXYD5, GAB2, GNA12, IRS2, L1CAM, MITF, MX1, PAK1, PIK3R2, PTK2, RELA, RNF144A, SDC4, SKP2, SP1, TCF12, TGFA, TIMP3	26
Cellular Movement	invasion	invasion of breast cancer cell lines	9.18E-03	ACSL4, CAV1, CBL, CTSK, FXYD5, GAB2, IRS2, PAK1, PTK2, RELA, RNF144A	11
Cellular Movement	invasion	invasion of cells	9.51E-03	ACSL4, CAV1, CBL, CDK5R1, CMTM8, CTSK, ELK1, ETV4, FXYD5, GAB2, GNA12, IRS2, L1CAM, MITF, MX1, PAK1, PIK3R2, PTK2, RELA, RGS4, RNF144A, SDC4, SKP2, SP1, TCF12, TGFA, TIMP3	27
Cellular Movement	invasion	invasion of nasopharyngeal carcinoma cells	2.77E-02	GNA12	1
Cellular Movement	chemotaxis	chemotaxis of mammary tumor cells	2.77E-02	CBL	1
Cellular Movement	contact repulsion	contact repulsion of axons	2.77E-02	L1CAM	1
Hereditary Disorder	Wolfram Syndrome	Wolfram Syndrome	7.65E-04	CISD2, WFS1	2
Hereditary Disorder	inborn error of carbohydrate metabolism	inborn error of carbohydrate metabolism	9.83E-04	ALG3, ARSB, DOLK, GALE, GNPTAB, MGAT2, MPDU1, NEU1	8
Hereditary Disorder	Huntington's disease	Huntington's Disease	1.31E-03	CAMKK2, CASP2, CDK5R1, DHCR7, DPYSL3, EMX2, ETV4, FGF13, GIT1, HSPA5, ITPKB, MICALL1, MT1X, MT2A, NOTCH2, NRGN, PFN2, PPARGC1A, PPRC1, RGS4, SCARB2, SDC4, SETDB1, SH3BP4, SORT1, SRM, STOM, TGM2, TRAK2, UBE2D4	30
Hereditary Disorder	osteogenesis imperfecta type II	osteogenesis imperfecta type II	2.25E-03	COL1A2, CRTAP	2
Hereditary	Krabbe's disease	Krabbe's disease	4.42E-03	CAV1, FLOT2	2

Disorder					
Hereditary Disorder	Ehlers-Danlos syndrome	Ehlers-Danlos syndrome	4.90E-03	B4GALT7, COL1A2, PLOD1	3
Hereditary Disorder	congenital disorders of glycosylation	congenital disorders of glycosylation	8.98E-03	ALG3, DOLK, MGAT2, MPDU1	4
Hereditary Disorder	mucopolysaccharidoses	mucopolysaccharidoses	9.02E-03	ARSB, GNPTAB, NEU1	3
Hereditary Disorder	Alagille syndrome 2	Alagille syndrome 2	2.77E-02	NOTCH2	1
Hereditary Disorder	Alzheimer's disease type 4	Alzheimer's disease type 4	2.77E-02	PSEN2	1
Hereditary Disorder	CRASH syndrome	CRASH syndrome	2.77E-02	L1CAM	1
Hereditary Disorder	Charcot-Marie-Tooth disease axonal type 1c	Charcot-Marie-Tooth disease axonal type 1c	2.77E-02	LITAF	1
Hereditary Disorder	Hailey-Hailey disease	Hailey-Hailey disease	2.77E-02	ATP2C1	1
Hereditary Disorder	Methylmalonic aciduria and homocystinuria cblC type	Methylmalonic aciduria and homocystinuria cblC type	2.77E-02	MMACHC	1
Hereditary Disorder	Parkinson disease 1	Parkinson disease 1	2.77E-02	SNCA	1
Hereditary Disorder	Schnyder crystalline corneal dystrophy	Schnyder crystalline corneal dystrophy	2.77E-02	UBIAD1	1
Hereditary Disorder	Smith-Lemli-Opitz syndrome	Smith-Lemli-Opitz syndrome	2.77E-02	DHCR7	1
Hereditary Disorder	Smith-McCort dysplasia	Smith-McCort dysplasia	2.77E-02	DYM	1
Hereditary Disorder	Wiskott-Aldrich syndrome type 2	Wiskott-Aldrich syndrome type 2	2.77E-02	WIPF1	1
Hereditary Disorder	X-linked hydrocephalus with congenital idiopathic intestinal pseudoobstruction	X-linked hydrocephalus with congenital idiopathic intestinal pseudoobstruction	2.77E-02	L1CAM	1
Hereditary Disorder	agammaglobulinemia type 5	agammaglobulinemia type 5	2.77E-02	LRRC8A	1
Hereditary	albinism-deafness	albinism-deafness syndrome of tietz	2.77E-02	MITF	1

Disorder	syndrome of tietz				
Hereditary Disorder	amish infantile epilepsy syndrome	amish infantile epilepsy syndrome	2.77E-02	ST3GAL5	1
Hereditary Disorder	arterial tortuosity syndrome	arterial tortuosity syndrome	2.77E-02	SLC2A10	1
Hereditary Disorder	autosomal dominant Parkinson disease 4	autosomal dominant Parkinson disease 4	2.77E-02	SNCA	1
Hereditary Disorder	bilateral frontoparietal polymicrogyria	bilateral frontoparietal polymicrogyria	2.77E-02	GPR56	1
Hereditary Disorder	cerebral dysgenesis, neuropathy, ichthyosis, and palmoplantar keratoderma syndrome, cednik syndrome	cerebral dysgenesis, neuropathy, ichthyosis, and palmoplantar keratoderma syndrome, cednik syndrome	2.77E-02	SNAP29	1
Hereditary Disorder	congenital disorder of glycosylation type 1d	congenital disorder of glycosylation type 1d	2.77E-02	ALG3	1
Hereditary Disorder	congenital disorder of glycosylation type 1f	congenital disorder of glycosylation type 1f	2.77E-02	MPDU1	1
Hereditary Disorder	congenital disorder of glycosylation type 1m	congenital disorder of glycosylation type 1m	2.77E-02	DOLK	1
Hereditary Disorder	congenital disorders of glycosylation type 2a	congenital disorders of glycosylation type 2a	2.77E-02	MGAT2	1
Hereditary Disorder	congenital generalized lipodystrophy type 3	congenital generalized lipodystrophy type 3	2.77E-02	CAV1	1
Hereditary Disorder	creatine deficiency syndrome	creatine deficiency syndrome	2.77E-02	SLC6A8	1
Hereditary Disorder	desmosterolosis	desmosterolosis	2.77E-02	DHCR24	1
Hereditary Disorder	dyggve-melchior-clausen syndrome	dyggve-melchior-clausen syndrome	2.77E-02	DYM	1
Hereditary Disorder	epidermolysis bullosa simplex with muscular dystrophy	epidermolysis bullosa simplex with muscular dystrophy	2.77E-02	PLEC	1
Hereditary Disorder	epidermolysis bullosa simplex, ogna type	epidermolysis bullosa simplex, ogna type	2.77E-02	PLEC	1
Hereditary Disorder	idiopathic basal ganglia calcification	idiopathic basal ganglia calcification type 3	2.77E-02	SLC20A2	1

	type 3				
Hereditary Disorder	mucopolysaccharidosis type VI	mucopolysaccharidosis type VI	2.77E-02	ARSB	1
Hereditary Disorder	nonsyndromic low frequency sensorineural hearing loss	nonsyndromic low frequency sensorineural hearing loss	2.77E-02	WFS1	1
Hereditary Disorder	osteogenesis imperfecta type IIB	osteogenesis imperfecta type IIB	2.77E-02	CRTAP	1
Hereditary Disorder	overhydrated hereditary stomatocytosis	overhydrated hereditary stomatocytosis	2.77E-02	STOM	1
Hereditary Disorder	progeroid form Ehlers-Danlos syndrome	progeroid form Ehlers-Danlos syndrome	2.77E-02	B4GALT7	1
Hereditary Disorder	pseudo-hurler polydystrophy	pseudo-hurler polydystrophy	2.77E-02	GNPTAB	1
Hereditary Disorder	pyknodysostosis	pyknodysostosis	2.77E-02	CTSK	1
Hereditary Disorder	udpglucose-4-epimerase deficiency	udpglucose-4-epimerase deficiency	2.77E-02	GALE	1
Hereditary Disorder	lysosomal storage disease	lysosomal storage disease	3.01E-02	ARSB, CAV1, FLOT2, GNPTAB, NEU1	5
Metabolic Disease	Wolfram Syndrome	Wolfram Syndrome	7.65E-04	CISD2, WFS1	2
Metabolic Disease	inborn error of carbohydrate metabolism	inborn error of carbohydrate metabolism	9.83E-04	ALG3, ARSB, DOLK, GALE, GNPTAB, MGAT2, MPDU1, NEU1	8
Metabolic Disease	osteogenesis imperfecta type II	osteogenesis imperfecta type II	2.25E-03	COL1A2, CRTAP	2
Metabolic Disease	Krabbe's disease	Krabbe's disease	4.42E-03	CAV1, FLOT2	2
Metabolic Disease	Ehlers-Danlos syndrome	Ehlers-Danlos syndrome	4.90E-03	B4GALT7, COL1A2, PLOD1	3
Metabolic Disease	congenital disorders of glycosylation	congenital disorders of glycosylation	8.98E-03	ALG3, DOLK, MGAT2, MPDU1	4
Metabolic Disease	mucopolysaccharidoses	mucopolysaccharidoses	9.02E-03	ARSB, GNPTAB, NEU1	3
Metabolic	metabolic bone	metabolic bone disease	2.41E-02	B4GALT7, COL1A2, CRTAP, PLOD1	4

Disease	disease				
Metabolic Disease	Methylmalonic aciduria and homocystinuria cblC type	Methylmalonic aciduria and homocystinuria cblC type	2.77E-02	MMACHC	1
Metabolic Disease	Smith-Lemli-Opitz syndrome	Smith-Lemli-Opitz syndrome	2.77E-02	DHCR7	1
Metabolic Disease	albinism-deafness syndrome of tietz	albinism-deafness syndrome of tietz	2.77E-02	MITF	1
Metabolic Disease	alkalinization	alkalinization of fibroblasts	2.77E-02	SLC9A1	1
Metabolic Disease	congenital disorder of glycosylation type 1d	congenital disorder of glycosylation type 1d	2.77E-02	ALG3	1
Metabolic Disease	congenital disorder of glycosylation type 1f	congenital disorder of glycosylation type 1f	2.77E-02	MPDU1	1
Metabolic Disease	congenital disorder of glycosylation type 1m	congenital disorder of glycosylation type 1m	2.77E-02	DOLK	1
Metabolic Disease	congenital disorders of glycosylation type 2a	congenital disorders of glycosylation type 2a	2.77E-02	MGAT2	1
Metabolic Disease	creatine deficiency syndrome	creatine deficiency syndrome	2.77E-02	SLC6A8	1
Metabolic Disease	desmosterolosis	desmosterolosis	2.77E-02	DHCR24	1
Metabolic Disease	mucopolysaccharidosis type VI	mucopolysaccharidosis type VI	2.77E-02	ARSB	1
Metabolic Disease	osteogenesis imperfecta type IIB	osteogenesis imperfecta type IIB	2.77E-02	CRTAP	1
Metabolic Disease	progeroid form Ehlers-Danlos syndrome	progeroid form Ehlers-Danlos syndrome	2.77E-02	B4GALT7	1
Metabolic Disease	pseudo-hurler polydystrophy	pseudo-hurler polydystrophy	2.77E-02	GNPTAB	1
Metabolic Disease	udpglucose-4-epimerase deficiency	udpglucose-4-epimerase deficiency	2.77E-02	GALE	1
Metabolic Disease	lysosomal storage disease	lysosomal storage disease	3.01E-02	ARSB, CAV1, FLOT2, GNPTAB, NEU1	5
Metabolic Disease	insulin-dependent diabetes mellitus	insulin-dependent diabetes mellitus	4.52E-02	AGPAT1, COL1A2, DYM, HCP5, HMGN4, PPP1R11, RAB5B, RNF5, RSBN1, SH2B3, WFS1	11
Ophthalmic Disease	Wolfram Syndrome	Wolfram Syndrome	7.65E-04	CISD2, WFS1	2

Ophthalmic Disease	Schnyder crystalline corneal dystrophy	Schnyder crystalline corneal dystrophy	2.77E-02	UBIAD1	1
Developmental Disorder	inborn error of carbohydrate metabolism	inborn error of carbohydrate metabolism	9.83E-04	ALG3, ARSB, DOLK, GALE, GNPTAB, MGAT2, MPDU1, NEU1	8
Developmental Disorder	osteogenesis imperfecta type II	osteogenesis imperfecta type II	2.25E-03	COL1A2, CRTAP	2
Developmental Disorder	Krabbe's disease	Krabbe's disease	4.42E-03	CAV1, FLOT2	2
Developmental Disorder	Ehlers-Danlos syndrome	Ehlers-Danlos syndrome	4.90E-03	B4GALT7, COL1A2, PLOD1	3
Developmental Disorder	congenital disorders of glycosylation	congenital disorders of glycosylation	8.98E-03	ALG3, DOLK, MGAT2, MPDU1	4
Developmental Disorder	mucopolysaccharidose s	mucopolysaccharidoses	9.02E-03	ARSB, GNPTAB, NEU1	3
Developmental Disorder	arrhythmogenic right ventricular dysplasia	arrhythmogenic right ventricular dysplasia	2.42E-02	DSG2, TMEM43	2
Developmental Disorder	Alagille syndrome 2	Alagille syndrome 2	2.77E-02	NOTCH2	1
Developmental Disorder	Hajdu-Cheney syndrome	Hajdu-Cheney syndrome	2.77E-02	NOTCH2	1
Developmental Disorder	Methylmalonic aciduria and homocystinuria cblC type	Methylmalonic aciduria and homocystinuria cblC type	2.77E-02	MMACHC	1
Developmental Disorder	Smith-Lemli-Opitz syndrome	Smith-Lemli-Opitz syndrome	2.77E-02	DHCR7	1
Developmental Disorder	Smith-McCort dysplasia	Smith-McCort dysplasia	2.77E-02	DYM	1
Developmental Disorder	X-linked hydrocephalus with congenital idiopathic intestinal pseudoobstruction	X-linked hydrocephalus with congenital idiopathic intestinal pseudoobstruction	2.77E-02	L1CAM	1
Developmental Disorder	albinism-deafness syndrome of tietz	albinism-deafness syndrome of tietz	2.77E-02	MITF	1
Developmental Disorder	arrhythmogenic right ventricular dysplasia familial 10	arrhythmogenic right ventricular dysplasia familial 10	2.77E-02	DSG2	1

Developmental Disorder	bilateral frontoparietal polymicrogyria	bilateral frontoparietal polymicrogyria	2.77E-02	GPR56	1
Developmental Disorder	congenital disorder of glycosylation type 1d	congenital disorder of glycosylation type 1d	2.77E-02	ALG3	1
Developmental Disorder	congenital disorder of glycosylation type 1f	congenital disorder of glycosylation type 1f	2.77E-02	MPDU1	1
Developmental Disorder	congenital disorder of glycosylation type 1m	congenital disorder of glycosylation type 1m	2.77E-02	DOLK	1
Developmental Disorder	congenital disorders of glycosylation type 2a	congenital disorders of glycosylation type 2a	2.77E-02	MGAT2	1
Developmental Disorder	congenital generalized lipodystrophy type 3	congenital generalized lipodystrophy type 3	2.77E-02	CAV1	1
Developmental Disorder	creatine deficiency syndrome	creatine deficiency syndrome	2.77E-02	SLC6A8	1
Developmental Disorder	dyggve-melchior-clausen syndrome	dyggve-melchior-clausen syndrome	2.77E-02	DYM	1
Developmental Disorder	epidermolysis bullosa simplex with muscular dystrophy	epidermolysis bullosa simplex with muscular dystrophy	2.77E-02	PLEC	1
Developmental Disorder	epidermolysis bullosa simplex, ogna type	epidermolysis bullosa simplex, ogna type	2.77E-02	PLEC	1
Developmental Disorder	mucopolysaccharidosis type VI	mucopolysaccharidosis type VI	2.77E-02	ARSB	1
Developmental Disorder	osteogenesis imperfecta type IIB	osteogenesis imperfecta type IIB	2.77E-02	CRTAP	1
Developmental Disorder	progeroid form Ehlers-Danlos syndrome	progeroid form Ehlers-Danlos syndrome	2.77E-02	B4GALT7	1
Developmental Disorder	pseudo-hurler polydystrophy	pseudo-hurler polydystrophy	2.77E-02	GNPTAB	1
Developmental Disorder	pyknodysostosis	pyknodysostosis	2.77E-02	CTSK	1
Developmental Disorder	udpglucose-4-epimerase deficiency	udpglucose-4-epimerase deficiency	2.77E-02	GALE	1
Developmental Disorder	dysplasia	dysplasia	2.78E-02	B4GALT7, CANT1, COL1A2, DYM, GNA12, NOTCH2, PLOD1	7
Developmental Disorder	lysosomal storage disease	lysosomal storage disease	3.01E-02	ARSB, CAV1, FLOT2, GNPTAB, NEU1	5
Developmental	skeletal dysplasia	skeletal dysplasia	3.87E-02	B4GALT7, CANT1, COL1A2, DYM, NOTCH2,	6

Disorder				PLOD1	
Skeletal and Muscular Disorders	Huntington's disease	Huntington's Disease	1.31E-03	CAMKK2, CASP2, CDK5R1, DHCR7, DPYSL3, EMX2, ETV4, FGF13, GIT1, HSPA5, ITPKB, MICALL1, MT1X, MT2A, NOTCH2, NRGN, PFN2, PPARGC1A, PPRC1, RGS4, SCARB2, SDC4, SETDB1, SH3BP4, SORT1, SRM, STOM, TGM2, TRAK2, UBE2D4	30
Skeletal and Muscular Disorders	neuromuscular disease	neuromuscular disease	2.14E-03	CAMKK2, CASP2, CDK5R1, DBNL, DHCR7, DPYSL3, EMX2, ENO2, ETV4, FGF13, GIT1, HSPA5, ITPKB, MICALL1, MT1X, MT2A, MX1, NOTCH2, NRGN, PAK1, PFN2, PPARGC1A, PPRC1, RELA, RGS4, RNF114, RNF5, RPL15, SCARB2, SDC4, SETDB1, SH3BP4, SNCA, SORT1, SRM, STOM, TGM2, TMED10, TRAK2, UBE2D4	40
Skeletal and Muscular Disorders	osteogenesis imperfecta type II	osteogenesis imperfecta type II	2.25E-03	COL1A2, CRTAP	2
Skeletal and Muscular Disorders	Ehlers-Danlos syndrome	Ehlers-Danlos syndrome	4.90E-03	B4GALT7, COL1A2, PLOD1	3
Skeletal and Muscular Disorders	metabolic bone disease	metabolic bone disease	2.41E-02	B4GALT7, COL1A2, CRTAP, PLOD1	4
Skeletal and Muscular Disorders	Charcot-Marie-Tooth disease axonal type 1c	Charcot-Marie-Tooth disease axonal type 1c	2.77E-02	LITAF	1
Skeletal and Muscular Disorders	Hajdu-Cheney syndrome	Hajdu-Cheney syndrome	2.77E-02	NOTCH2	1
Skeletal and Muscular Disorders	Parkinson disease 1	Parkinson disease 1	2.77E-02	SNCA	1
Skeletal and Muscular Disorders	Smith-McCort dysplasia	Smith-McCort dysplasia	2.77E-02	DYM	1
Skeletal and Muscular Disorders	autosomal dominant Parkinson disease 4	autosomal dominant Parkinson disease 4	2.77E-02	SNCA	1

Skeletal and Muscular Disorders	cerebral dysgenesis, neuropathy, ichthyosis, and palmoplantar keratoderma syndrome, cednik syndrome	cerebral dysgenesis, neuropathy, ichthyosis, and palmoplantar keratoderma syndrome, cednik syndrome	2.77E-02	SNAP29	1
Skeletal and Muscular Disorders	dyggve-melchior-clausen syndrome	dyggve-melchior-clausen syndrome	2.77E-02	DYM	1
Skeletal and Muscular Disorders	epidermolysis bullosa simplex with muscular dystrophy	epidermolysis bullosa simplex with muscular dystrophy	2.77E-02	PLEC	1
Skeletal and Muscular Disorders	osteogenesis imperfecta type IIB	osteogenesis imperfecta type IIB	2.77E-02	CRTAP	1
Skeletal and Muscular Disorders	progeroid form Ehlers-Danlos syndrome	progeroid form Ehlers-Danlos syndrome	2.77E-02	B4GALT7	1
Skeletal and Muscular Disorders	pyknodysostosis	pyknodysostosis	2.77E-02	CTSK	1
Skeletal and Muscular Disorders	skeletal dysplasia	skeletal dysplasia	3.87E-02	B4GALT7, CANT1, COL1A2, DYM, NOTCH2, PLOD1	6
Cell-To-Cell Signaling and Interaction	adhesion	adhesion of hOB cells	2.25E-03	SDC4, TGM2	2
Cell-To-Cell Signaling and Interaction	quantity	quantity of focal adhesions	2.20E-02	CAV1, GIT1, PTK2	3
Cell-To-Cell Signaling and Interaction	quantity	quantity of monoamines	4.20E-02	RAF1, SNCA	2
Cell-To-Cell Signaling and Interaction	accumulation	accumulation of 2-arachidonoylglycerol	2.77E-02	MGLL	1
Cell-To-Cell Signaling and	adherence	adherence of carcinoma cell lines	2.77E-02	TIMP3	1

Interaction					
Cell-To-Cell Signaling and Interaction	adherence	adherence of thyroid tumor cell lines	2.77E-02	TIMP3	1
Cell-To-Cell Signaling and Interaction	attraction	attraction of axons	2.77E-02	L1CAM	1
Cell-To-Cell Signaling and Interaction	cell-cell contact	cell-cell contact of colon cancer cell lines	2.77E-02	TCF12	1
Cell-To-Cell Signaling and Interaction	contact repulsion	contact repulsion of axons	2.77E-02	L1CAM	1
Cell-To-Cell Signaling and Interaction	detachment	detachment of melanoma cell lines	2.77E-02	PTK2	1
Cell-To-Cell Signaling and Interaction	excitation	excitation of hippocampal neurons	2.77E-02	SNCA	1
Cell-To-Cell Signaling and Interaction	inflammatory response	inflammatory response of monocyte-derived dendritic cells	2.77E-02	RELA	1
Cell-To-Cell Signaling and Interaction	localization	localization of focal adhesions	2.77E-02	PTK2	1
Cell-To-Cell Signaling and Interaction	morphology	morphology of synapse	2.77E-02	SNCA	1
Cell-To-Cell Signaling and Interaction	recognition	recognition of fibroblast cell lines	2.77E-02	RELA	1
Cell-To-Cell Signaling and Interaction	recognition	recognition of lung cell lines	2.77E-02	RELA	1
Cell-To-Cell Signaling and Interaction	recovery	recovery of synapse	2.77E-02	SNAP29	1
Cell-To-Cell	stimulation	stimulation of chronic lymphocytic	2.77E-02	TXLNA	1

Signaling and Interaction		leukemia cells			
Cell-To-Cell Signaling and Interaction	binding	binding of kidney cell lines	2.83E-02	CAV1, SDC4, SORT1, ST6GAL1	4
Cell-To-Cell Signaling and Interaction	binding	binding of embryonic cell lines	3.09E-02	CAV1, SDC4, SORT1	3
Cell-To-Cell Signaling and Interaction	binding	binding of epithelial cell lines	3.77E-02	CAV1, SDC4, SORT1	3
Connective Tissue Development and Function	adhesion	adhesion of hOB cells	2.25E-03	SDC4, TGM2	2
Connective Tissue Development and Function	proliferation	proliferation of fibroblast cell lines	1.65E-02	CBL, FOXO3, PTK2, RAF1, TGM2	5
Connective Tissue Development and Function	S phase	initiation of S phase of fibroblasts	2.77E-02	SKP2	1
Connective Tissue Disorders	osteogenesis imperfecta type II	osteogenesis imperfecta type II	2.25E-03	COL1A2, CRTAP	2
Connective Tissue Disorders	Ehlers-Danlos syndrome	Ehlers-Danlos syndrome	4.90E-03	B4GALT7, COL1A2, PLOD1	3
Connective Tissue Disorders	metabolic bone disease	metabolic bone disease	2.41E-02	B4GALT7, COL1A2, CRTAP, PLOD1	4
Connective Tissue Disorders	Hajdu-Cheney syndrome	Hajdu-Cheney syndrome	2.77E-02	NOTCH2	1
Connective Tissue Disorders	Smith-McCort dysplasia	Smith-McCort dysplasia	2.77E-02	DYM	1

Connective Tissue Disorders	alkalinization	alkalinization of fibroblasts	2.77E-02	SLC9A1	1
Connective Tissue Disorders	arterial tortuosity syndrome	arterial tortuosity syndrome	2.77E-02	SLC2A10	1
Connective Tissue Disorders	dyggve-melchior-clausen syndrome	dyggve-melchior-clausen syndrome	2.77E-02	DYM	1
Connective Tissue Disorders	epidermolysis bullosa simplex with muscular dystrophy	epidermolysis bullosa simplex with muscular dystrophy	2.77E-02	PLEC	1
Connective Tissue Disorders	epidermolysis bullosa simplex, ogna type	epidermolysis bullosa simplex, ogna type	2.77E-02	PLEC	1
Connective Tissue Disorders	osteogenesis imperfecta type IIB	osteogenesis imperfecta type IIB	2.77E-02	CRTAP	1
Connective Tissue Disorders	progeroid form Ehlers-Danlos syndrome	progeroid form Ehlers-Danlos syndrome	2.77E-02	B4GALT7	1
Connective Tissue Disorders	pyknodysostosis	pyknodysostosis	2.77E-02	CTSK	1
Connective Tissue Disorders	skeletal dysplasia	skeletal dysplasia	3.87E-02	B4GALT7, CANT1, COL1A2, DYM, NOTCH2, PLOD1	6
Skeletal and Muscular System Development and Function	adhesion	adhesion of hOB cells	2.25E-03	SDC4, TGM2	2
Skeletal and Muscular System Development and Function	cell movement	cell movement of vascular smooth muscle cells	1.54E-02	CLIC4, GIT1, SDC4, TRIB1	4
Skeletal and	cell spreading	cell spreading of vascular smooth muscle	2.77E-02	GIT1	1

Muscular System Development and Function		cells			
Skeletal and Muscular System Development and Function	migration	migration of vascular smooth muscle cells	4.93E-02	CLIC4, GIT1, SDC4	3
Tissue Development	adhesion	adhesion of hOB cells	2.25E-03	SDC4, TGM2	2
Tissue Development	formation	formation of fibrils	1.07E-02	IDE, SERPINH1, SNCA	3
Tissue Development	formation	formation of collagen fibrils	2.77E-02	SERPINH1	1
Tissue Development	formation	formation of nerve fascicle	2.77E-02	L1CAM	1
Tissue Development	formation	formation of protofibrils	2.77E-02	SNCA	1
Tissue Development	formation	formation of microtubules	3.09E-02	DPYSL2, PSRC1, TBCD	3
Tissue Development	formation	formation of filaments	3.78E-02	CAV1, DPYSL2, IDE, PAK1, PSRC1, PTK2, SERPINH1, SIRPA, SNCA, TBCD	10
Tissue Development	tubulation	tubulation of epithelial tissue	1.91E-02	CAV1, FOXO3, RAF1, RGS4, TIMP3	5
Tissue Development	tubulation	delay in tubulation of epithelial cells	2.77E-02	RGS4	1
Tissue Development	tubulation	tubulation of HAEC cells	2.77E-02	RAF1	1
Tissue Development	quantity	quantity of focal adhesions	2.20E-02	CAV1, GIT1, PTK2	3
Tissue Development	adherence	adherence of carcinoma cell lines	2.77E-02	TIMP3	1
Tissue Development	adherence	adherence of thyroid tumor cell lines	2.77E-02	TIMP3	1
Tissue Development	cell-cell contact	cell-cell contact of colon cancer cell lines	2.77E-02	TCF12	1
Tissue	detachment	detachment of melanoma cell lines	2.77E-02	PTK2	1

Development					
Tissue Development	development	development of pyramidal tract	2.77E-02	L1CAM	1
Tissue Development	localization	localization of focal adhesions	2.77E-02	PTK2	1
Tissue Development	polymerization	polymerization of actin stress fibers	2.77E-02	PAK1	1
Cell Signaling	protein kinase cascade	protein kinase cascade	2.26E-03	ATP2C1, CAMKK2, CANT1, CXXC5, FGF13, ICK, LITAF, MKNK2, NDFIP2, NMI, OXSR1, RYK, SHISA5, SLC35B2, TGFA, TRIB1, UBE2N	17
Cell Signaling	entrance	entrance of Ca2+	3.96E-03	CAV1, MX1, ORAI1, PSEN2	4
Cell Signaling	I-kappaB kinase/NF-kappaB cascade	I-kappaB kinase/NF-kappaB cascade	8.23E-03	ATP2C1, CANT1, CXXC5, LITAF, NDFIP2, SHISA5, SLC35B2, UBE2N	8
Cell Signaling	diffusion	diffusion of Ca2+	2.77E-02	VDAC1	1
Cell Signaling	leakage	leakage of Ca2+	2.77E-02	TMBIM6	1
Infectious Disease	replication	replication of virus	2.72E-03	AGFG1, AP1M1, ATG7, ATP2C1, CANT1, CASP2, CLIC4, ILF3, LINGO1, MX1, MX2, NUDCD3, OXSR1, PAK2, PLK4, PPARGC1A, RAF1, RELA, RFFL, SIGMAR1, STX5, TRIM25, TRPV2, UBE2E2, UBE3C, UBQLN4	26
Infectious Disease	replication	replication of RNA virus	9.09E-03	AGFG1, AP1M1, ATG7, ATP2C1, CANT1, CASP2, CLIC4, ILF3, LINGO1, MX1, NUDCD3, OXSR1, PAK2, PLK4, RAF1, RELA, RFFL, SIGMAR1, STX5, TRIM25, TRPV2, UBE2E2, UBQLN4	23
Infectious Disease	replication	replication of Influenza A virus	1.21E-02	ATP2C1, CANT1, CLIC4, ILF3, LINGO1, MX1, NUDCD3, OXSR1, PAK2, PLK4, RELA, RFFL, SIGMAR1, STX5, TRPV2, UBQLN4	16
Infectious Disease	replication	replication of Thogoto virus	2.77E-02	MX1	1
Infectious Disease	infection	Viral Infection	3.79E-03	AGFG1, AP1M1, AP3M1, ATG7, ATP2C1, BCL9, C20orf24, CAMKK2, CANT1, CASP2, CAV1, CBL, CBLB, CLIC4, COG2, CRTC3, DAZAP2, DHX33, DSG2, FLOT2, HCP5, HSPA5, ILF3, IRS2, LINGO1, MGLL, MICALL1, MRPL10, MT1X, MT2A, MX1, MX2, NUDCD3, OSBPL3, OXSR1, PAK1, PAK2, PDE7B, PDXK, PIGH, PIK3CB, PKMYT1, PLK4, PPARGC1A, PSEN2, RAF1, RELA, REPIN1, RFFL, RNF144A, SH2B3, SIGMAR1, SSU72, ST3GAL5,	69

				ST6GAL1, STX5, TMC6, TRIM25, TRIM8, TROVE2, TRPV2, TYRO3, UBE2E2, UBE2Z, UBE3C, UBQLN4, USP39, VPS4A, WASF2	
Infectious Disease	infection	infection by Zaire ebolavirus	9.02E-03	HSPA5, PAK1, TYRO3	3
Infectious Disease	infection	infection of respiratory tract	1.59E-02	C20orf24, FLOT2, IRS2, PDXK, PIK3CB, SH2B3, SIGMAR1, ST6GAL1, TROVE2	9
Infectious Disease	infection	infection of leukemia cell lines	4.88E-02	PAK1, TYRO3	2
Infectious Disease	Ebola hemorrhagic fever	Ebola hemorrhagic fever	3.96E-03	HSPA5, MGLL, PAK1, TYRO3	4
Infectious Disease	localization	localization of virus	4.42E-03	CBL, VPS4A	2
Infectious Disease	severe acute respiratory syndrome	severe acute respiratory syndrome	1.84E-02	C20orf24, FLOT2, IRS2, PDXK, PIK3CB, SH2B3, ST6GAL1, TROVE2	8
Infectious Disease	quantity	quantity of flavivirus	2.77E-02	HSPA5	1
Infectious Disease	uptake	uptake of Human adenovirus 35	2.77E-02	PAK1	1
Infectious Disease	budding	budding of lentivirus	2.97E-02	AP1M1, VPS4A	2
Cell Death and Survival	cell death	cell death of cortical neurons	3.38E-03	RELA, SNCA, SP1, TGFA	4
Cell Death and Survival	cell death	cell death of epithelial cells	6.80E-03	ATG7, CASP2, CDC37, HSPA5, MITF, NRGN, PAK2, PRMT2, PTK2, RGS4, SDHC, SNCA, TCF12, TIMP3, VDAC1, VOPP1	16
Cell Death and Survival	cell death	cell death of skin cancer cell lines	1.07E-02	ATG7, CHEK1, RELA	3
Cell Death and Survival	cell death	cell death of tumor cell lines	1.43E-02	ARMC10, ATG7, BIRC7, CADM1, CASP2, CAV1, CDK2, CDK5R1, CHEK1, DCT, DCTD, DHCR24, DNAJC15, DSG2, EGR3, ELK1, FOXO3, GLO1, GRINA, HSPA5, MCRS1, MIF4GD, MITF, MT1X, MT2A, NOTCH2, NUDCD3, ORAI1, PAK1, PAK2, PIK3CB, PKMYT1, PPARGC1A, PPP1R11, PRR7, PSEN2, PTK2, RABL6, RAF1, RELA, RNF5, SDHC, SIRPA, SKP2, SNCA, SP1, ST6GAL1, TGFA, TGM2, TIMP3, TMBIM6, TMED10, VDAC1, VOPP1, XPR1	55
Cell Death and	cell death	cell death of breast cancer cell lines	1.59E-02	ATG7, CASP2, CAV1, CHEK1, DNAJC15, ELK1,	16

Survival				FOXO3, HSPA5, PIK3CB, PTK2, RABL6, RAF1, RELA, RNF5, SDHC, SP1	
Cell Death and Survival	cell death	cell death of prostate cancer cell lines	1.94E-02	CAV1, CDK5R1, CHEK1, FOXO3, ORAI1, PAK1, RAF1, RELA, TGFA, TMBIM6, TMED10	11
Cell Death and Survival	cell death	cell death of cervical cancer cell lines	2.46E-02	ATG7, BIRC7, CASP2, CHEK1, GRINA, MCRS1, MIF4GD, NUDCD3, PAK1, PAK2, PSEN2, RAF1, SNCA, TIMP3, VDAC1, VOPP1	16
Cell Death and Survival	cell death	cell death of embryonic cell lines	3.17E-02	ATG7, CASP2, HSPA5, NRGN, PAK2, PRMT2, RGS4, SDHC, SNCA, TCF12, VDAC1, VOPP1	12
Cell Death and Survival	cell death	cell death of epithelial cell lines	4.04E-02	ATG7, CASP2, HSPA5, NRGN, PAK2, PRMT2, RGS4, SDHC, SNCA, TCF12, VDAC1, VOPP1	12
Cell Death and Survival	cell death	cell death of lymphoblastoid cell lines	4.82E-02	CADM1, CASP2, CAV1, CDK2, CHEK1, DCTD	6
Cell Death and Survival	condensation	condensation of chromatin	3.96E-03	CDK2, CHEK1, RAF1, SETD8	4
Cell Death and Survival	apoptosis	apoptosis of melanoma cell lines	5.45E-03	BIRC7, CHEK1, DCT, FOXO3, HSPA5, MITF, PKMYT1, RNF5	8
Cell Death and Survival	apoptosis	apoptosis of embryonic cell lines	7.45E-03	CASP2, HSPA5, NRGN, PAK2, PRMT2, RGS4, SDHC, SNCA, TCF12, VDAC1, VOPP1	11
Cell Death and Survival	apoptosis	apoptosis of epithelial cell lines	1.12E-02	CASP2, HSPA5, NRGN, PAK2, PRMT2, RGS4, SDHC, SNCA, TCF12, VDAC1, VOPP1	11
Cell Death and Survival	apoptosis	apoptosis of tumor cell lines	1.70E-02	ARMC10, ATG7, BIRC7, CASP2, CAV1, CDK2, CDK5R1, CHEK1, DCT, DNAJC15, DSG2, EGR3, FOXO3, GLO1, HSPA5, MCRS1, MITF, MT2A, ORAI1, PAK1, PAK2, PIK3CB, PKMYT1, PPARGC1A, PPP1R11, PRR7, PSEN2, PTK2, RABL6, RAF1, RELA, RNF5, SDHC, SIRPA, SKP2, SNCA, SP1, ST6GAL1, TGFA, TGM2, TIMP3, TMBIM6, TMED10, VDAC1, VOPP1, XPR1	46
Cell Death and Survival	apoptosis	apoptosis of prostate cancer cell lines	1.93E-02	CAV1, CDK5R1, FOXO3, ORAI1, PAK1, RAF1, RELA, TGFA, TMBIM6, TMED10	10
Cell Death and Survival	apoptosis	apoptosis of kidney cell lines	2.36E-02	CASP2, HSPA5, NRGN, PAK2, PRMT2, RGS4, SDHC, SNCA, TCF12, VDAC1, VOPP1	11
Cell Death and Survival	apoptosis	apoptosis of leukemia cell lines	2.57E-02	CASP2, CAV1, EGR3, FOXO3, GLO1, ORAI1, PAK1, PAK2, PPP1R11, PRR7, PTK2, RAF1, RELA, TGFA	14
Cell Death and Survival	apoptosis	apoptosis of microvascular endothelial cells	2.77E-02	FOXO3, HSPA5, VDAC1	3

Cell Death and Survival	apoptosis	apoptosis of breast cancer cell lines	4.30E-02	ATG7, CASP2, CAV1, CHEK1, FOXO3, HSPA5, PIK3CB, PTK2, RABL6, RAF1, RELA, RNF5, SDHC	13
Cell Death and Survival	atrophy	atrophy of myofiber	2.77E-02	FOXO3	1
Cell Death and Survival	degeneration	degeneration of striatonigral neurons	2.77E-02	SNCA	1
Cell Death and Survival	maturation	maturation of apoptotic bodies	2.77E-02	CASP2	1
Cell Death and Survival	cell viability	cell viability of stomach cancer cell lines	4.88E-02	CAV1, PTK2	2
DNA Replication, Recombination, and Repair	condensation	condensation of chromatin	3.96E-03	CDK2, CHEK1, RAF1, SETD8	4
DNA Replication, Recombination, and Repair	S phase checkpoint control	S phase checkpoint control of tumor cell lines	2.42E-02	CHEK1, CKS2	2
DNA Replication, Recombination, and Repair	S phase checkpoint control	S phase checkpoint control of breast cancer cell lines	2.77E-02	CKS2	1
DNA Replication, Recombination, and Repair	S phase checkpoint control	S phase checkpoint control of lymphoblastoid cell lines	2.77E-02	CHEK1	1
DNA Replication, Recombination, and Repair	DNA replication checkpoint	DNA replication checkpoint of cervical cancer cell lines	2.77E-02	CHEK1	1
DNA Replication, Recombination, and Repair	DNA replication checkpoint	DNA replication checkpoint of colon cancer cell lines	2.77E-02	CHEK1	1
DNA Replication, Recombination, and Repair	deamination	deamination of adenosine	2.77E-02	ADAT1	1

DNA Replication, Recombination, and Repair	homologous recombination	homologous recombination of gene	2.77E-02	CDK2	1
DNA Replication, Recombination, and Repair	quantity	quantity of mitotic spindle	4.20E-02	NUDCD3, PAK1	2
Hematological Disease	Ebola hemorrhagic fever	Ebola hemorrhagic fever	3.96E-03	HSPA5, MGLL, PAK1, TYRO3	4
Hematological Disease	infection	infection by Zaire ebolavirus	9.02E-03	HSPA5, PAK1, TYRO3	3
Hematological Disease	infection	infection of leukemia cell lines	4.88E-02	PAK1, TYRO3	2
Hematological Disease	agammaglobulinemia type 5	agammaglobulinemia type 5	2.77E-02	LRRC8A	1
Hematological Disease	centroblastic lymphoma	centroblastic lymphoma	2.77E-02	CASP2	1
Hematological Disease	overhydrated hereditary stomatocytosis	overhydrated hereditary stomatocytosis	2.77E-02	STOM	1
Molecular Transport	entrance	entrance of Ca2+	3.96E-03	CAV1, MX1, ORAI1, PSEN2	4
Molecular Transport	accumulation	accumulation of 2-arachidonoylglycerol	2.77E-02	MGLL	1
Molecular Transport	clearance	clearance of synthetic promoter	2.77E-02	SP1	1
Molecular Transport	secretion	secretion of lysophosphatidic acid	2.77E-02	AGK	1
Molecular Transport	transport	transport of dADP	2.77E-02	SLC25A19	1
Molecular Transport	transport	transport of dATP	2.77E-02	SLC25A19	1
Molecular Transport	transport	transport of dCDP	2.77E-02	SLC25A19	1
Molecular Transport	transport	transport of dGDP	2.77E-02	SLC25A19	1
Molecular	transport	transport of dGTP	2.77E-02	SLC25A19	1

Transport					
Molecular Transport	transport	transport of dTDP	2.77E-02	SLC25A19	1
Molecular Transport	transport	transport of dTTP	2.77E-02	SLC25A19	1
Molecular Transport	transport	transport of deoxycytidine triphosphate	2.77E-02	SLC25A19	1
Molecular Transport	transport	transport of phosphoadenosine phosphosulfate	2.77E-02	SLC35B2	1
Molecular Transport	uptake	uptake of creatine	2.77E-02	SLC6A8	1
Molecular Transport	import	import of D-glucose	2.77E-02	IRS2, RHOQ, SORT1	3
Molecular Transport	quantity	quantity of monoamines	4.20E-02	RAF1, SNCA	2
Vitamin and Mineral Metabolism	entrance	entrance of Ca2+	3.96E-03	CAV1, MX1, ORAI1, PSEN2	4
Vitamin and Mineral Metabolism	synthesis	synthesis of cholesterol	6.11E-03	CAV1, DHCR24, DHCR7	3
Vitamin and Mineral Metabolism	biosynthesis	biosynthesis of dolichol monophosphate	2.77E-02	DOLK	1
Vitamin and Mineral Metabolism	biosynthesis	biosynthesis of pyridoxal phosphate	2.77E-02	PDXK	1
Vitamin and Mineral Metabolism	diffusion	diffusion of Ca2+	2.77E-02	VDAC1	1
Vitamin and Mineral Metabolism	leakage	leakage of Ca2+	2.77E-02	TMBIM6	1
Cellular Assembly and Organization	biogenesis	biogenesis of mitochondria	4.42E-03	CRTC3, PPARGC1A	2
Cellular Assembly and	quantity	quantity of granules	4.42E-03	RAF1, SNCA	2

Organization					
Cellular Assembly and Organization	quantity	quantity of focal adhesions	2.20E-02	CAV1, GIT1, PTK2	3
Cellular Assembly and Organization	quantity	quantity of nerve ending	2.77E-02	SNCA	1
Cellular Assembly and Organization	quantity	quantity of perikaryon	2.77E-02	SNCA	1
Cellular Assembly and Organization	quantity	quantity of secretory granules	2.77E-02	RAF1	1
Cellular Assembly and Organization	quantity	quantity of tubulovesicular structures	2.77E-02	CAV1	1
Cellular Assembly and Organization	quantity	quantity of mitotic spindle	4.20E-02	NUDCD3, PAK1	2
Cellular Assembly and Organization	organization	organization of vesicles	7.24E-03	CAV1, SORT1	2
Cellular Assembly and Organization	organization	organization of organelle	1.85E-02	ARSB, B4GALT7, CAV1, COG2, COL1A2, PEX19, PSRC1, RHOQ, SERP1, SORT1, SRF, TMED9	12
Cellular Assembly and Organization	retraction	retraction of cellular tail	7.24E-03	ADCY9, PAK1	2
Cellular Assembly and Organization	formation	formation of fibrils	1.07E-02	IDE, SERPINH1, SNCA	3
Cellular Assembly and Organization	formation	formation of autolysosomes	2.77E-02	VPS4A	1
Cellular Assembly and Organization	formation	formation of collagen fibrils	2.77E-02	SERPINH1	1
Cellular	formation	formation of macropinosomes	2.77E-02	WASF2	1

Assembly and Organization					
Cellular Assembly and Organization	formation	formation of protofibrils	2.77E-02	SNCA	1
Cellular Assembly and Organization	formation	formation of microtubules	3.09E-02	DPYSL2, PSRC1, TBCD	3
Cellular Assembly and Organization	formation	formation of filaments	3.78E-02	CAV1, DPYSL2, IDE, PAK1, PSRC1, PTK2, SERPINH1, SIRPA, SNCA, TBCD	10
Cellular Assembly and Organization	targeting	targeting of vesicles	1.46E-02	SNAP29, STX5	2
Cellular Assembly and Organization	association	association of actin cytoskeleton	2.77E-02	FXYD5	1
Cellular Assembly and Organization	binding	binding of Golgi membrane	2.77E-02	TMED10	1
Cellular Assembly and Organization	binding	binding of nucleoprotein filaments	2.77E-02	UBE2N	1
Cellular Assembly and Organization	development	development of fibrillar inclusions	2.77E-02	SNCA	1
Cellular Assembly and Organization	development	development of cytoplasm	2.86E-02	CAV1, CRTC3, DPYSL2, PAK1, PEX19, PPARGC1A, PSRC1, PTK2, SIRPA, TBCD, VPS4A	11
Cellular Assembly and Organization	disruption	disruption of cortical actin filaments	2.77E-02	RHOQ	1
Cellular Assembly and Organization	disruption	disruption of fibrils	2.77E-02	SNCA	1
Cellular Assembly and Organization	disruption	disruption of vesicle aggregates	2.77E-02	SNCA	1

Cellular Assembly and Organization	localization	localization of focal adhesions	2.77E-02	PTK2	1
Cellular Assembly and Organization	maturation	maturation of apoptotic bodies	2.77E-02	CASP2	1
Cellular Assembly and Organization	polymerization	polymerization of actin stress fibers	2.77E-02	PAK1	1
Cellular Assembly and Organization	production	production of cytoplasmic aggregates	2.77E-02	SNCA	1
Cellular Assembly and Organization	fusion	fusion of endosomes	4.88E-02	C20orf24, CBL	2
Cell Morphology	tubulation	tubulation of cells	4.66E-03	CAV1, CLIC4, FOXO3, ORAI1, RAF1, RGS4, TIMP3	7
Cell Morphology	tubulation	delay in tubulation of epithelial cells	2.77E-02	RGS4	1
Cell Morphology	tubulation	tubulation of HAEC cells	2.77E-02	RAF1	1
Cell Morphology	repair	repair of colon cancer cell lines	7.24E-03	CHEK1, UBE2N	2
Cell Morphology	autophagy	autophagy of bone cancer cell lines	1.46E-02	ATG7, RELA	2
Cell Morphology	morphology	morphology of carcinoma cell lines	1.47E-02	GNA12, RAF1, STMN3	3
Cell Morphology	morphology	morphology of sarcoma cell lines	2.77E-02	TIMP3	1
Cell Morphology	morphology	morphology of synapse	2.77E-02	SNCA	1
Cell Morphology	polarization	polarization of tumor cell lines	2.47E-02	DPYSL2, GNA12, PAK1	3
Cell Morphology	polarization	polarization of leukemia cell lines	4.20E-02	DPYSL2, GNA12	2
Cell Morphology	cell spreading	cell spreading of vascular smooth muscle cells	2.77E-02	GIT1	1
Cell	cell spreading	cell spreading of breast cancer cell lines	2.97E-02	CBL, PAK1	2

Morphology					
Cell Morphology	enlargement	enlargement of fibroblast cell lines	2.77E-02	MCRS1	1
Cell Morphology	enlargement	enlargement of fibrosarcoma cell lines	2.77E-02	MCRS1	1
Cell Morphology	enlargement	enlargement of lung cell lines	2.77E-02	MCRS1	1
Cell Morphology	mineralization	mineralization of bone cancer cell lines	2.77E-02	SNCA	1
Cell Morphology	pH	pH of neurons	2.77E-02	L1CAM	1
Cell Morphology	pH	pH of cells	4.88E-02	L1CAM, SLC9A1	2
Cell Morphology	swelling	swelling of lysosome	2.77E-02	VPS26A	1
Cell Morphology	budding	budding of lentivirus	2.97E-02	AP1M1, VPS4A	2
Dermatological Diseases and Conditions	Ehlers-Danlos syndrome	Ehlers-Danlos syndrome	4.90E-03	B4GALT7, COL1A2, PLOD1	3
Dermatological Diseases and Conditions	skin squamous cell carcinoma	skin squamous cell carcinoma	2.47E-02	CAV1, HSPA5, TGFA	3
Dermatological Diseases and Conditions	Hailey-Hailey disease	Hailey-Hailey disease	2.77E-02	ATP2C1	1
Dermatological Diseases and Conditions	cerebral dysgenesis, neuropathy, ichthyosis, and palmoplantar keratoderma syndrome, cednik syndrome	cerebral dysgenesis, neuropathy, ichthyosis, and palmoplantar keratoderma syndrome, cednik syndrome	2.77E-02	SNAP29	1
Dermatological Diseases and Conditions	epidermolysis bullosa simplex with muscular dystrophy	epidermolysis bullosa simplex with muscular dystrophy	2.77E-02	PLEC	1
Dermatological Diseases and Conditions	epidermolysis bullosa simplex, ogna type	epidermolysis bullosa simplex, ogna type	2.77E-02	PLEC	1

Dermatological Diseases and Conditions	nodular basal cell carcinoma	nodular basal cell carcinoma	2.77E-02	SMO	1
Dermatological Diseases and Conditions	progeroid form Ehlers-Danlos syndrome	progeroid form Ehlers-Danlos syndrome	2.77E-02	B4GALT7	1
Organismal Injury and Abnormalities	Ehlers-Danlos syndrome	Ehlers-Danlos syndrome	4.90E-03	B4GALT7, COL1A2, PLOD1	3
Organismal Injury and Abnormalities	atrophy	atrophy of myofiber	2.77E-02	FOXO3	1
Organismal Injury and Abnormalities	atrophy	atrophy of myotube	2.77E-02	FOXO3	1
Organismal Injury and Abnormalities	bilateral frontoparietal polymicrogyria	bilateral frontoparietal polymicrogyria	2.77E-02	GPR56	1
Organismal Injury and Abnormalities	epidermolysis bullosa simplex with muscular dystrophy	epidermolysis bullosa simplex with muscular dystrophy	2.77E-02	PLEC	1
Organismal Injury and Abnormalities	epidermolysis bullosa simplex, ogna type	epidermolysis bullosa simplex, ogna type	2.77E-02	PLEC	1
Organismal Injury and Abnormalities	idiopathic basal ganglia calcification type 3	idiopathic basal ganglia calcification type 3	2.77E-02	SLC20A2	1
Organismal Injury and Abnormalities	progeroid form Ehlers-Danlos syndrome	progeroid form Ehlers-Danlos syndrome	2.77E-02	B4GALT7	1
Cellular Compromise	endoplasmic reticulum stress response	endoplasmic reticulum stress response	5.21E-03	CDC37, HSPA5, MGAT2, SERPINH1, TMBIM6, WFS1	6
Cellular Compromise	endoplasmic reticulum stress response	endoplasmic reticulum stress response of cells	7.95E-03	CDC37, HSPA5, MGAT2, TMBIM6	4
Cellular Compromise	endoplasmic reticulum stress response	endoplasmic reticulum stress response of embryonic cell lines	1.46E-02	CDC37, MGAT2	2
Cellular Compromise	endoplasmic reticulum stress response	endoplasmic reticulum stress response of kidney cell lines	1.46E-02	CDC37, MGAT2	2

Cellular Compromise	endoplasmic reticulum stress response	endoplasmic reticulum stress response of epithelial cell lines	2.42E-02	CDC37, MGAT2	2
Cellular Compromise	endoplasmic reticulum stress response	endoplasmic reticulum stress response of cancer cells	2.77E-02	TMBIM6	1
Cellular Compromise	degradation	degradation of filaments	7.24E-03	CTSK, PAK1	2
Cellular Compromise	degradation	degradation of amyloid fibrils	2.77E-02	CTSK	1
Cellular Compromise	retraction	retraction of cellular tail	7.24E-03	ADCY9, PAK1	2
Cellular Compromise	retraction	retraction of cells	2.42E-02	CDK2, PAK1	2
Cellular Compromise	atrophy	atrophy of myofiber	2.77E-02	FOXO3	1
Cellular Compromise	atrophy	atrophy of myotube	2.77E-02	FOXO3	1
Cellular Compromise	degeneration	degeneration of striatonigral neurons	2.77E-02	SNCA	1
Cellular Compromise	development	development of fibrillar inclusions	2.77E-02	SNCA	1
Cellular Compromise	disruption	disruption of cortical actin filaments	2.77E-02	RHOQ	1
Cellular Compromise	disruption	disruption of fibrils	2.77E-02	SNCA	1
Cellular Compromise	disruption	disruption of vesicle aggregates	2.77E-02	SNCA	1
Cellular Compromise	dysfunction	dysfunction of neurons	2.77E-02	SNCA	1
Cellular Compromise	loss	loss of fibrils	2.77E-02	CAV1	1
Cellular Compromise	swelling	swelling of lysosome	2.77E-02	VPS26A	1
Cellular Function and Maintenance	endoplasmic reticulum stress response	endoplasmic reticulum stress response	5.21E-03	CDC37, HSPA5, MGAT2, SERPINH1, TMBIM6, WFS1	6
Cellular Function and Maintenance	endoplasmic reticulum stress response	endoplasmic reticulum stress response of cells	7.95E-03	CDC37, HSPA5, MGAT2, TMBIM6	4

Cellular Function and Maintenance	endoplasmic reticulum stress response	endoplasmic reticulum stress response of embryonic cell lines	1.46E-02	CDC37, MGAT2	2
Cellular Function and Maintenance	endoplasmic reticulum stress response	endoplasmic reticulum stress response of kidney cell lines	1.46E-02	CDC37, MGAT2	2
Cellular Function and Maintenance	endoplasmic reticulum stress response	endoplasmic reticulum stress response of epithelial cell lines	2.42E-02	CDC37, MGAT2	2
Cellular Function and Maintenance	endoplasmic reticulum stress response	endoplasmic reticulum stress response of cancer cells	2.77E-02	TMBIM6	1
Cellular Function and Maintenance	repair	repair of colon cancer cell lines	7.24E-03	CHEK1, UBE2N	2
Cellular Function and Maintenance	autophagy	autophagy of bone cancer cell lines	1.46E-02	ATG7, RELA	2
Cellular Function and Maintenance	formation	formation of macropinosomes	2.77E-02	WASF2	1
Cellular Function and Maintenance	formation	formation of microtubules	3.09E-02	DPYSL2, PSRC1, TBCD	3
Cellular Function and Maintenance	localization	localization of focal adhesions	2.77E-02	PTK2	1
Cellular Function and Maintenance	mineralization	mineralization of bone cancer cell lines	2.77E-02	SNCA	1
Cellular Function and Maintenance	pH	pH of neurons	2.77E-02	L1CAM	1
Cellular Function and Maintenance	pH	pH of cells	4.88E-02	L1CAM, SLC9A1	2
Cellular Function and	polymerization	polymerization of actin stress fibers	2.77E-02	PAK1	1

Maintenance					
Cellular Function and Maintenance	import	import of D-glucose	2.77E-02	IRS2, RHOQ, SORT1	3
Lipid Metabolism	synthesis	synthesis of cholesterol	6.11E-03	CAV1, DHCR24, DHCR7	3
Lipid Metabolism	synthesis	synthesis of sphingomyelin	1.07E-02	OSBP, SLC27A1	2
Lipid Metabolism	synthesis	synthesis of glycolipid	1.42E-02	MPDU1, OSBP, SLC27A1, ST3GAL5, ST8SIA5	5
Lipid Metabolism	synthesis	synthesis of phosphatidylserine	2.77E-02	SLC27A1	1
Lipid Metabolism	synthesis	synthesis of glycosphingolipid	3.79E-02	OSBP, SLC27A1, ST3GAL5, ST8SIA5	4
Lipid Metabolism	phosphorylation	phosphorylation of phosphatidic acid	2.42E-02	AGK, PIK3CB	2
Lipid Metabolism	phosphorylation	phosphorylation of diacylglycerol	2.77E-02	AGK	1
Lipid Metabolism	phosphorylation	phosphorylation of lysophosphatidic acid	2.77E-02	AGK	1
Lipid Metabolism	accumulation	accumulation of 2-arachidonoylglycerol	2.77E-02	MGLL	1
Lipid Metabolism	biosynthesis	biosynthesis of dolichol monophosphate	2.77E-02	DOLK	1
Lipid Metabolism	biosynthesis	biosynthesis of phosphatidylglycerol	2.77E-02	SLC27A1	1
Lipid Metabolism	incorporation	incorporation of lignoceric acid	2.77E-02	SLC27A1	1
Lipid Metabolism	secretion	secretion of lysophosphatidic acid	2.77E-02	AGK	1
Lipid Metabolism	trafficking	trafficking of cholesterol	2.77E-02	CAV1	1
Lipid Metabolism	tubulation	tubulation of lipid	2.77E-02	MX1	1
Small Molecule Biochemistry	synthesis	synthesis of cholesterol	6.11E-03	CAV1, DHCR24, DHCR7	3
Small Molecule Biochemistry	synthesis	synthesis of sphingomyelin	1.07E-02	OSBP, SLC27A1	2

Small Molecule Biochemistry	synthesis	synthesis of glycolipid	1.42E-02	MPDU1, OSBP, SLC27A1, ST3GAL5, ST8SIA5	5
Small Molecule Biochemistry	synthesis	synthesis of phosphatidylserine	2.77E-02	SLC27A1	1
Small Molecule Biochemistry	synthesis	synthesis of spermidine	2.77E-02	SRM	1
Small Molecule Biochemistry	synthesis	synthesis of glycosphingolipid	3.79E-02	OSBP, SLC27A1, ST3GAL5, ST8SIA5	4
Small Molecule Biochemistry	binding	binding of ATP	1.92E-02	HSPBP1, NR1H2	2
Small Molecule Biochemistry	binding	binding of haloperidol	2.77E-02	SIGMAR1	1
Small Molecule Biochemistry	phosphorylation	phosphorylation of phosphatidic acid	2.42E-02	AGK, PIK3CB	2
Small Molecule Biochemistry	phosphorylation	phosphorylation of diacylglycerol	2.77E-02	AGK	1
Small Molecule Biochemistry	phosphorylation	phosphorylation of lysophosphatidic acid	2.77E-02	AGK	1
Small Molecule Biochemistry	phosphorylation	phosphorylation of L-threonine	4.88E-02	CDK5R1, CHEK1	2
Small Molecule Biochemistry	accumulation	accumulation of 2-arachidonoylglycerol	2.77E-02	MGLL	1
Small Molecule Biochemistry	accumulation	accumulation of UDP-D-glucose	2.77E-02	GALE	1
Small Molecule Biochemistry	accumulation	accumulation of UDP-N-acetylglucosamine	2.77E-02	GALE	1
Small Molecule Biochemistry	accumulation	accumulation of UDP-galactose	2.77E-02	GALE	1
Small Molecule Biochemistry	biosynthesis	biosynthesis of dolichol monophosphate	2.77E-02	DOLK	1
Small Molecule Biochemistry	biosynthesis	biosynthesis of hydroxylysine	2.77E-02	PLOD1	1
Small Molecule Biochemistry	biosynthesis	biosynthesis of phosphatidylglycerol	2.77E-02	SLC27A1	1
Small Molecule Biochemistry	branching	branching of N-glycan	2.77E-02	CAV1	1
Small Molecule Biochemistry	catabolism	catabolism of galactose	2.77E-02	GALE	1

Small Molecule Biochemistry	conjugation	conjugation of histamine	2.77E-02	TGM2	1
Small Molecule Biochemistry	deamination	deamination of adenosine	2.77E-02	ADAT1	1
Small Molecule Biochemistry	deamination	deamination of dCMP	2.77E-02	DCTD	1
Small Molecule Biochemistry	decarboxylation	decarboxylation of L-glutamic acid	2.77E-02	GAD1	1
Small Molecule Biochemistry	incorporation	incorporation of lignoceric acid	2.77E-02	SLC27A1	1
Small Molecule Biochemistry	secretion	secretion of lysophosphatidic acid	2.77E-02	AGK	1
Small Molecule Biochemistry	trafficking	trafficking of cholesterol	2.77E-02	CAV1	1
Small Molecule Biochemistry	transamidation	transamidation of histamine	2.77E-02	TGM2	1
Small Molecule Biochemistry	transport	transport of dADP	2.77E-02	SLC25A19	1
Small Molecule Biochemistry	transport	transport of dATP	2.77E-02	SLC25A19	1
Small Molecule Biochemistry	transport	transport of dCDP	2.77E-02	SLC25A19	1
Small Molecule Biochemistry	transport	transport of dGDP	2.77E-02	SLC25A19	1
Small Molecule Biochemistry	transport	transport of dGTP	2.77E-02	SLC25A19	1
Small Molecule Biochemistry	transport	transport of dTTP	2.77E-02	SLC25A19	1
Small Molecule Biochemistry	transport	transport of deoxycytidine triphosphate	2.77E-02	SLC25A19	1
Small Molecule Biochemistry	transport	transport of phosphoadenosine phosphosulfate	2.77E-02	SLC35B2	1
Small Molecule Biochemistry	tubulation	tubulation of lipid	2.77E-02	MX1	1
Small Molecule Biochemistry	uptake	uptake of creatine	2.77E-02	SLC6A8	1
Small Molecule Biochemistry	import	import of D-glucose	2.77E-02	IRS2, RHOQ, SORT1	3

Small Molecule Biochemistry	quantity	quantity of monoamines	4.20E-02	RAF1, SNCA	2
Cell Cycle	G1 phase	arrest in G1 phase of lung cancer cell lines	7.00E-03	NOTCH2, RAF1, RFFL, TUSC2	4
Cell Cycle	G1 phase	arrest in G1 phase	4.13E-02	CAMKK2, CDK2, NOTCH2, PSEN2, RAF1, RFFL, SKP2, SP1, TCF12, TUSC2	10
Cell Cycle	interphase	arrest in interphase of lung cancer cell lines	8.56E-03	CHEK1, NOTCH2, RAF1, RFFL, TUSC2	5
Cell Cycle	interphase	arrest in interphase of epithelial cells	1.46E-02	CHEK1, SP1	2
Cell Cycle	interphase	arrest in interphase	3.74E-02	AGK, ATF5, CAMKK2, CDK2, CHEK1, FOXO3, KDM5B, NOTCH2, PSEN2, RAF1, RFFL, SKP2, SP1, TCF12, TUSC2	15
Cell Cycle	interphase	interphase of tumor cell lines	4.13E-02	AGK, ATF5, CAMKK2, CDK2, CHEK1, FOXO3, MT1A, NOTCH2, PKMYT1, PSEN2, RAF1, RFFL, SKP2, SP1, TGFA, TUSC2	16
Cell Cycle	interphase	interphase of prostate cancer cell lines	4.52E-02	AGK, SKP2, SP1	3
Cell Cycle	senescence	senescence of lung cell lines	1.01E-02	CAV1, MCRS1, RAF1, RELA	4
Cell Cycle	S phase	re-entry into S phase	1.69E-02	CDK2, SKP2, TGFA	3
Cell Cycle	S phase	S phase of tumor cell lines	2.10E-02	AGK, CDK2, CHEK1, FOXO3, RAF1, SKP2, TGFA	7
Cell Cycle	S phase	initiation of S phase of fibroblasts	2.77E-02	SKP2	1
Cell Cycle	S phase	S phase of prostate cancer cell lines	2.97E-02	AGK, SKP2	2
Cell Cycle	S phase	re-entry into S phase of tumor cell lines	4.88E-02	CDK2, TGFA	2
Cell Cycle	S phase checkpoint control	S phase checkpoint control of tumor cell lines	2.42E-02	CHEK1, CKS2	2
Cell Cycle	S phase checkpoint control	S phase checkpoint control of breast cancer cell lines	2.77E-02	CKS2	1
Cell Cycle	S phase checkpoint control	S phase checkpoint control of lymphoblastoid cell lines	2.77E-02	CHEK1	1
Cell Cycle	DNA replication checkpoint	DNA replication checkpoint of cervical cancer cell lines	2.77E-02	CHEK1	1
Cell Cycle	DNA replication checkpoint	DNA replication checkpoint of colon cancer cell lines	2.77E-02	CHEK1	1
Cell Cycle	G0/G1 phase transition	arrest in G0/G1 phase transition of myeloma cell lines	2.77E-02	CHEK1	1
Cell Cycle	G2/M phase	entry into G2/M phase of bone cancer cell lines	2.77E-02	PKMYT1	1
Cell Cycle	G2/M phase transition	arrest in G2/M phase transition of hepatoma cell lines	2.77E-02	ATF5	1

Cell Cycle	aneuploidization	aneuploidization of tumor cell lines	2.77E-02	MCRS1	1
Cell Cycle	cleavage	arrest in cleavage of blastomeres	2.77E-02	RAF1	1
Cell Cycle	homologous recombination	homologous recombination of gene	2.77E-02	CDK2	1
Cell Cycle	late G1 phase	late G1 phase of colon cancer cell lines	2.77E-02	MT1A	1
Cell Cycle	mitosis	arrest in mitosis of prostate cancer cell lines	2.77E-02	CHEK1	1
Cell Cycle	mitosis	entry into mitosis of bone cancer cell lines	2.77E-02	PKMYT1	1
Cell Cycle	mitosis	entry into mitosis of pancreatic cancer cell lines	2.77E-02	CHEK1	1
Cell Cycle	ploidy	ploidy of bladder cancer cell lines	2.77E-02	UBIAD1	1
Cell Cycle	premature senescence	premature senescence of fibrosarcoma cell lines	2.77E-02	MCRS1	1
Cell Cycle	cell cycle progression	cell cycle progression of lymphoblastoid cell lines	3.57E-02	CASP2, PSEN2	2
Cellular Development	epithelial-mesenchymal transition	epithelial-mesenchymal transition of pancreatic cancer cell lines	7.24E-03	CAV1, SP1	2
Cellular Development	senescence	senescence of lung cell lines	1.01E-02	CAV1, MCRS1, RAF1, RELA	4
Cellular Development	proliferation	proliferation of lung cancer cell lines	1.50E-02	CASP2, CAV1, CDK2, CHEK1, EPB41L3, NOTCH2, PIK3CB, RAF1, RELA, SKP2, STMN3, TUSC2, UHRF1	13
Cellular Development	proliferation	proliferation of fibroblast cell lines	1.65E-02	CBL, FOXO3, PTK2, RAF1, TGM2	5
Cellular Development	proliferation	proliferation of lung cancer cells	3.57E-02	CAV1, SKP2	2
Cellular Development	proliferation	proliferation of hepatocytes	4.20E-02	SKP2, TGFA	2
Cellular Development	branching	branching of N-glycan	2.77E-02	CAV1	1
Cellular Development	clonal expansion	clonal expansion of myeloma cell lines	2.77E-02	SMO	1
Cellular Development	colony formation	colony formation of non-small-cell lung cancer cells	2.77E-02	CAV1	1
Cellular Development	colony formation	colony formation of small cell lung cancer cells	2.77E-02	CAV1	1

Cellular Development	quantity	quantity of carcinoma cells	2.77E-02	FOXO3	1
Cellular Development	tubulation	delay in tubulation of epithelial cells	2.77E-02	RGS4	1
Cellular Development	tubulation	tubulation of HAEC cells	2.77E-02	RAF1	1
Embryonic Development	apoptosis	apoptosis of embryonic cell lines	7.45E-03	CASP2, HSPA5, NRGN, PAK2, PRMT2, RGS4, SDHC, SNCA, TCF12, VDAC1, VOPP1	11
Embryonic Development	binding	binding of embryonic cell lines	3.09E-02	CAV1, SDC4, SORT1	3
Embryonic Development	cell death	cell death of embryonic cell lines	3.17E-02	ATG7, CASP2, HSPA5, NRGN, PAK2, PRMT2, RGS4, SDHC, SNCA, TCF12, VDAC1, VOPP1	12
Post-Translational Modification	ubiquitination	ubiquitination	8.45E-03	BIRC7, CBL, HSPBP1, KLHL21, NDFIP2, RNF20, RNF5, RUSC1, SKP1/SKP1P2, UBE2N, UBE3C	11
Post-Translational Modification	ubiquitination	ubiquitination of protein	1.93E-02	BIRC7, CBL, HSPBP1, KLHL21, NDFIP2, RNF20, RUSC1, SKP1/SKP1P2, UBE2N, UBE3C	10
Post-Translational Modification	methylation	methylation of protein	2.42E-02	EPB41L3, PRMT2	2
Post-Translational Modification	decarboxylation	decarboxylation of L-glutamic acid	2.77E-02	GAD1	1
Post-Translational Modification	hydroxylation	hydroxylation of protein fragment	2.77E-02	PLOD1	1
Post-Translational Modification	polyubiquitination	polyubiquitination	4.33E-02	RNF20, RUSC1, UBE2N, UBE3C	4
Post-Translational Modification	phosphorylation	phosphorylation of L-threonine	4.88E-02	CDK5R1, CHEK1	2
Respiratory System Development and Function	senescence	senescence of lung cell lines	1.01E-02	CAV1, MCRS1, RAF1, RELA	4
Cancer	extrahepatic bile duct	extrahepatic bile duct cancer	1.07E-02	POLE3, POLE4	2

	cancer				
Cancer	metastasis	metastasis	1.29E-02	CAV1, DPYSL2, DSG2, EMX2, EPB41L3, GNA12, GREB1, GYG2, MITF, POLE3, POLE4, PTK2, RAF1, RGS4, SEL1L3, SMO, TCF12	17
Cancer	early-onset breast cancer	early-onset breast cancer	1.46E-02	CHEK1, EI24	2
Cancer	transformation	cell transformation	2.29E-02	CBL, HYAL2, L1CAM, PIK3CB, RAF1, RELA, SIRPA, SKP2	8
Cancer	skin squamous cell carcinoma	skin squamous cell carcinoma	2.47E-02	CAV1, HSPA5, TGFA	3
Cancer	metastatic colorectal cancer	metastatic colorectal cancer	2.48E-02	CAV1, DPYSL2, DSG2, EMX2, EPB41L3, GREB1, GYG2, RGS4, SEL1L3, TCF12	10
Cancer	cancer	liver cancer	2.75E-02	ATF5, ATP1B3, CASP2, CKS2, ETV4, FAM83D, GPAA1, HSPA5, MCRS1, MT2A, PAK1, PAQR4, PLK4, POLE3, POLE4, PPP1R3C, RAF1, RELA, SERF2, SMO, TRIM8	21
Cancer	cancer	Cancer	3.34E-02	ACTR3, ADAM19, ADCK2, AKR7A2, ARF4, ARHGAP1, ATF5, ATP1B3, BIRC7, BRWD1, CALM1 (includes others), CANT1, CAPN5, CASP2, CAV1, CBL, CD99L2, CDK2, CDK5R1, CHEK1, CKS2, CMTM4, CMTM8, CNRIP1, COL1A2, COMMD7, CTDSPL, CTSK, CXXC5, DBNL, DHCR24, DNAJC15, DPYSL2, DSG2, ECE2, EI24, EIF2AK1, EIF2S3, ELK1, EMX2, ENO2, EPB41L3, ETV4, EXTL3, FAM46C, FAM83D, FOXO3, FYCO1, GAB2, GAD1, GALE, GIT1, GLO1, GNA12, GPAA1, GREB1, GYG2, HRK, HSPA5, IGSF3, ILF3, L1CAM, LAD1, LYPLA2, MAU2, MAZ, MCRS1, MFSD5, MGLL, MICALL1, MITF, MKNK2, MT1X, MT2A, NEU1, NOTCH2, NPRL3, NPTX1, OLFML2A, PAK1, PAK2, PAQR4, PDE7B, PDXDC1, PDXK, PEX19, PHF16, PI15, PLAGL2, PLEC, PLK4, POLE3, POLE4, PPARGC1A, PPP1R3C, PRKRIR, PSEN2, PSME3, PTK2, RABL6, RAF1, RELA, RGS4, RNF144A, RNF181, RNF5, RPL15, SCARB2, SDHC, SEL1L3, SERF2, SGK223, SH2B3, SIGMAR1, SKP1/SKP1P2, SKP2, SLC27A1, SLC35A4, SLC44A1, SLC6A8, SLC7A1, SMO, SORT1, SRM,	144

				STMN3, STOM, TAPBP, TBC1D2B, TCF12, TGFA, TGM2, TIMP3, TMC6, TMEM158, TMEM97, TRIB1, TRIM8, TXLNA, UBXN2B, UHRF1, URB2, WFS1, XYLT1, ZBTB4	
Cancer	activation	activation of B-cell non-Hodgkin lymphoma	2.77E-02	TXLNA	1
Cancer	aneuploidization	aneuploidization of tumor cell lines	2.77E-02	MCRS1	1
Cancer	centroblastic lymphoma	centroblastic lymphoma	2.77E-02	CASP2	1
Cancer	chemosensitivity	chemosensitivity of melanoma cell lines	2.77E-02	MITF	1
Cancer	endoplasmic reticulum stress response	endoplasmic reticulum stress response of cancer cells	2.77E-02	TMBIM6	1
Cancer	invasion	invasion of nasopharyngeal carcinoma cells	2.77E-02	GNA12	1
Cancer	mammary adenocarcinoma	mammary adenocarcinoma	2.77E-02	RABL6	1
Cancer	nodular basal cell carcinoma	nodular basal cell carcinoma	2.77E-02	SMO	1
Cancer	sphere formation	sphere formation of ovarian cancer cell lines	2.77E-02	TGM2	1
Cancer	stimulation	stimulation of chronic lymphocytic leukemia cells	2.77E-02	TXLNA	1
Cancer	infection	infection of leukemia cell lines	4.88E-02	PAK1, TYRO3	2
Gastrointestinal Disease	extrahepatic bile duct cancer	extrahepatic bile duct cancer	1.07E-02	POLE3, POLE4	2
Gastrointestinal Disease	metastatic colorectal cancer	metastatic colorectal cancer	2.48E-02	CAV1, DPYSL2, DSG2, EMX2, EPB41L3, GREB1, GYG2, RGS4, SEL1L3, TCF12	10
Gastrointestinal Disease	cancer	liver cancer	2.75E-02	ATF5, ATP1B3, CASP2, CKS2, ETV4, FAM83D, GPAA1, HSPA5, MCRS1, MT2A, PAK1, PAQR4, PLK4, POLE3, POLE4, PPP1R3C, RAF1, RELA, SERF2, SMO, TRIM8	21
Gastrointestinal Disease	Alagille syndrome 2	Alagille syndrome 2	2.77E-02	NOTCH2	1
Gastrointestinal Disease	X-linked hydrocephalus with congenital idiopathic intestinal pseudoobstruction	X-linked hydrocephalus with congenital idiopathic intestinal pseudoobstruction	2.77E-02	L1CAM	1

Gastrointestinal Disease	insulin-dependent diabetes mellitus	insulin-dependent diabetes mellitus	4.52E-02	AGPAT1, COL1A2, DYM, HCP5, HMGN4, PPP1R11, RAB5B, RNF5, RSBN1, SH2B3, WFS1	11
Hepatic System Disease	extrahepatic bile duct cancer	extrahepatic bile duct cancer	1.07E-02	POLE3, POLE4	2
Hepatic System Disease	cancer	liver cancer	2.75E-02	ATF5, ATP1B3, CASP2, CKS2, ETV4, FAM83D, GPAA1, HSPA5, MCRS1, MT2A, PAK1, PAQR4, PLK4, POLE3, POLE4, PPP1R3C, RAF1, RELA, SERF2, SMO, TRIM8	21
Hepatic System Disease	Alagille syndrome 2	Alagille syndrome 2	2.77E-02	NOTCH2	1
Carbohydrate Metabolism	accumulation	accumulation of glycosides	1.46E-02	DNAJC15, GALE	2
Carbohydrate Metabolism	accumulation	accumulation of UDP-D-glucose	2.77E-02	GALE	1
Carbohydrate Metabolism	accumulation	accumulation of UDP-N-acetylglucosamine	2.77E-02	GALE	1
Carbohydrate Metabolism	accumulation	accumulation of UDP-galactose	2.77E-02	GALE	1
Carbohydrate Metabolism	metabolism	metabolism of carbohydrate	1.72E-02	AGK, AGPAT1, AKR7A2, B4GALT7, COG2, GALE, HYAL2, IRS2, MGAT2, MPDU1, RGS4, SLC27A1, SNCA, ST3GAL5, ST8SIA5, XYLT1	16
Carbohydrate Metabolism	phosphorylation	phosphorylation of phosphatidic acid	2.42E-02	AGK, PIK3CB	2
Carbohydrate Metabolism	phosphorylation	phosphorylation of lysophosphatidic acid	2.77E-02	AGK	1
Carbohydrate Metabolism	biosynthesis	biosynthesis of phosphatidylglycerol	2.77E-02	SLC27A1	1
Carbohydrate Metabolism	branching	branching of N-glycan	2.77E-02	CAV1	1
Carbohydrate Metabolism	catabolism	catabolism of galactose	2.77E-02	GALE	1
Carbohydrate Metabolism	secretion	secretion of lysophosphatidic acid	2.77E-02	AGK	1
Carbohydrate Metabolism	synthesis	synthesis of phosphatidylserine	2.77E-02	SLC27A1	1
Carbohydrate Metabolism	synthesis	synthesis of carbohydrate	4.13E-02	AGK, AGPAT1, B4GALT7, COG2, IRS2, MPDU1, RGS4, SLC27A1, SNCA, XYLT1	10
Carbohydrate	synthesis	synthesis of oligosaccharide	4.20E-02	COG2, MPDU1	2

Metabolism					
Carbohydrate Metabolism	import	import of D-glucose	2.77E-02	IRS2, RHOQ, SORT1	3
Endocrine System Disorders	glucose intolerance	glucose intolerance	1.46E-02	PSEN2, WFS1	2
Endocrine System Disorders	insulin-dependent diabetes mellitus	insulin-dependent diabetes mellitus	4.52E-02	AGPAT1, COL1A2, DYM, HCP5, HMGN4, PPP1R11, RAB5B, RNF5, RSBN1, SH2B3, WFS1	11
Cellular Growth and Proliferation	proliferation	proliferation of lung cancer cell lines	1.50E-02	CASP2, CAV1, CDK2, CHEK1, EPB41L3, NOTCH2, PIK3CB, RAF1, RELA, SKP2, STMN3, TUSC2, UHRF1	13
Cellular Growth and Proliferation	proliferation	proliferation of fibroblast cell lines	1.65E-02	CBL, FOXO3, PTK2, RAF1, TGM2	5
Cellular Growth and Proliferation	proliferation	proliferation of lung cancer cells	3.57E-02	CAV1, SKP2	2
Cellular Growth and Proliferation	proliferation	proliferation of hepatocytes	4.20E-02	SKP2, TGFA	2
Cellular Growth and Proliferation	colony formation	colony formation of lung cancer cell lines	1.54E-02	CADM1, PSRC1, RFFL, TUSC2	4
Cellular Growth and Proliferation	colony formation	colony formation of tumor cell lines	2.22E-02	ATG7, CADM1, CAV1, CBL, FOXO3, MITF, MT1X, PAK1, PSRC1, RELA, RFFL, SP1, TUSC2	13
Cellular Growth and Proliferation	colony formation	colony formation	2.30E-02	ATG7, CADM1, CAV1, CBL, CHEK1, EI24, FOXO3, GIT1, MITF, MT1X, PAK1, PSRC1, RAF1, RELA, RFFL, SP1, TUSC2	17
Cellular Growth and Proliferation	colony formation	colony formation of non-small-cell lung cancer cells	2.77E-02	CAV1	1
Cellular Growth and Proliferation	colony formation	colony formation of small cell lung cancer cells	2.77E-02	CAV1	1
Cellular Growth and	colony formation	colony formation of cells	3.71E-02	ATG7, CADM1, CAV1, CBL, CHEK1, EI24, FOXO3, MITF, MT1X, PAK1, PSRC1, RAF1, RELA, RFFL,	16

Proliferation				SP1, TUSC2	
Cellular Growth and Proliferation	clonal expansion	clonal expansion of myeloma cell lines	2.77E-02	SMO	1
Cellular Growth and Proliferation	excitation	excitation of hippocampal neurons	2.77E-02	SNCA	1
Cellular Growth and Proliferation	quantity	quantity of carcinoma cells	2.77E-02	FOXO3	1
Cellular Growth and Proliferation	stimulation	stimulation of chronic lymphocytic leukemia cells	2.77E-02	TXLNA	1
Respiratory Disease	infection	infection of respiratory tract	1.59E-02	C20orf24, FLOT2, IRS2, PDXK, PIK3CB, SH2B3, SIGMAR1, ST6GAL1, TROVE2	9
Respiratory Disease	severe acute respiratory syndrome	severe acute respiratory syndrome	1.84E-02	C20orf24, FLOT2, IRS2, PDXK, PIK3CB, SH2B3, ST6GAL1, TROVE2	8
Energy Production	binding	binding of ATP	1.92E-02	HSPBP1, NR1H2	2
Nucleic Acid Metabolism	binding	binding of ATP	1.92E-02	HSPBP1, NR1H2	2
Nucleic Acid Metabolism	accumulation	accumulation of UDP-D-glucose	2.77E-02	GALE	1
Nucleic Acid Metabolism	accumulation	accumulation of UDP-N-acetylglucosamine	2.77E-02	GALE	1
Nucleic Acid Metabolism	accumulation	accumulation of UDP-galactose	2.77E-02	GALE	1
Nucleic Acid Metabolism	deamination	deamination of adenosine	2.77E-02	ADAT1	1
Nucleic Acid Metabolism	deamination	deamination of dCMP	2.77E-02	DCTD	1
Nucleic Acid Metabolism	transport	transport of dADP	2.77E-02	SLC25A19	1
Nucleic Acid Metabolism	transport	transport of dATP	2.77E-02	SLC25A19	1
Nucleic Acid Metabolism	transport	transport of dCDP	2.77E-02	SLC25A19	1
Nucleic Acid	transport	transport of dGDP	2.77E-02	SLC25A19	1

Metabolism					
Nucleic Acid Metabolism	transport	transport of dGTP	2.77E-02	SLC25A19	1
Nucleic Acid Metabolism	transport	transport of dTTP	2.77E-02	SLC25A19	1
Nucleic Acid Metabolism	transport	transport of deoxycytidine triphosphate	2.77E-02	SLC25A19	1
Nucleic Acid Metabolism	transport	transport of phosphoadenosine phosphosulfate	2.77E-02	SLC35B2	1
Cardiovascular Disease	cardiomyopathy	cardiomyopathy	2.29E-02	DSG2, PDE7B, PSEN2, SCARB2, SLC6A8, TMEM43, TRIB1, XYLT1	8
Cardiovascular Disease	arrhythmogenic right ventricular dysplasia	arrhythmogenic right ventricular dysplasia	2.42E-02	DSG2, TMEM43	2
Cardiovascular Disease	arrhythmogenic right ventricular dysplasia familial 10	arrhythmogenic right ventricular dysplasia familial 10	2.77E-02	DSG2	1
Cardiovascular Disease	idiopathic basal ganglia calcification type 3	idiopathic basal ganglia calcification type 3	2.77E-02	SLC20A2	1
Amino Acid Metabolism	biosynthesis	biosynthesis of hydroxylysine	2.77E-02	PLOD1	1
Amino Acid Metabolism	decarboxylation	decarboxylation of L-glutamic acid	2.77E-02	GAD1	1
Amino Acid Metabolism	uptake	uptake of creatine	2.77E-02	SLC6A8	1
Amino Acid Metabolism	phosphorylation	phosphorylation of L-threonine	4.88E-02	CDK5R1, CHEK1	2
Antimicrobial Response	inhibition	inhibition of Influenza virus	2.77E-02	MX1	1
Antimicrobial Response	inhibition	inhibition of Thogoto virus	2.77E-02	MX1	1
Antimicrobial Response	inhibition	inhibition of vesicular stomatitis virus	2.77E-02	MX1	1
Auditory Disease	nonsyndromic low frequency sensorineural hearing loss	nonsyndromic low frequency sensorineural hearing loss	2.77E-02	WFS1	1
Behavior	punishment learning	punishment learning	2.77E-02	SNCA	1

Behavior	reward learning	reward learning	2.77E-02	SNCA	1
Cardiovascular System Development and Function	tubulation	tubulation of HAEC cells	2.77E-02	RAF1	1
Digestive System Development and Function	morphology	morphology of pancreas	2.77E-02	PSEN2	1
Drug Metabolism	binding	binding of haloperidol	2.77E-02	SIGMAR1	1
Endocrine System Development and Function	morphology	morphology of pancreas	2.77E-02	PSEN2	1
Free Radical Scavenging	scavenging	scavenging of hydrogen peroxide	2.77E-02	DHCR24	1
Gene Expression	binding	binding of NF-IL6 response element	2.77E-02	TGFA	1
Gene Expression	binding	binding of Tgf alpha response element	2.77E-02	TGFA	1
Immunological Disease	Wiskott-Aldrich syndrome type 2	Wiskott-Aldrich syndrome type 2	2.77E-02	WIPF1	1
Immunological Disease	agammaglobulinemia type 5	agammaglobulinemia type 5	2.77E-02	LRRC8A	1
Immunological Disease	insulin-dependent diabetes mellitus	insulin-dependent diabetes mellitus	4.52E-02	AGPAT1, COL1A2, DYM, HCP5, HMGN4, PPP1R11, RAB5B, RNF5, RSBN1, SH2B3, WFS1	11
Inflammatory Response	inflammatory response	inflammatory response of monocyte-derived dendritic cells	2.77E-02	RELA	1
Nervous System Development and Function	attraction	attraction of axons	2.77E-02	L1CAM	1
Nervous System Development and Function	contact repulsion	contact repulsion of axons	2.77E-02	L1CAM	1
Nervous	excitation	excitation of hippocampal neurons	2.77E-02	SNCA	1

System Development and Function					
Nervous System Development and Function	formation	formation of nerve fascicle	2.77E-02	L1CAM	1
Nervous System Development and Function	morphology	morphology of synapse	2.77E-02	SNCA	1
Nervous System Development and Function	pH	pH of neurons	2.77E-02	L1CAM	1
Nervous System Development and Function	quantity	quantity of nerve ending	2.77E-02	SNCA	1
Nervous System Development and Function	quantity	quantity of perikaryon	2.77E-02	SNCA	1
Organ Morphology	morphology	morphology of pancreas	2.77E-02	PSEN2	1
Organismal Development	growth	growth of Influenza B virus	2.77E-02	ST6GAL1	1
Organismal Development	tubulation	tubulation of HAEC cells	2.77E-02	RAF1	1
Psychological Disorders	Alzheimer's disease type 4	Alzheimer's disease type 4	2.77E-02	PSEN2	1
Psychological Disorders	creatine deficiency syndrome	creatine deficiency syndrome	2.77E-02	SLC6A8	1
Renal and Urological Disease	Methylmalonic aciduria and homocystinuria cblC type	Methylmalonic aciduria and homocystinuria cblC type	2.77E-02	MMACHC	1
Tissue Morphology	contraction	contraction of extracellular matrix	2.77E-02	CAV1	1

Tissue Morphology	degradation	degradation of bone	2.77E-02	CTSK	1
Tissue Morphology	morphology	morphology of synapse	2.77E-02	SNCA	1
Tissue Morphology	quantity	quantity of carcinoma cells	2.77E-02	FOXO3	1
Tissue Morphology	quantity	quantity of progenitor cells	2.77E-02	SH2B3	1
Tumor Morphology	activation	activation of B-cell non-Hodgkin lymphoma	2.77E-02	TXLNA	1
Tumor Morphology	colony formation	colony formation of non-small-cell lung cancer cells	2.77E-02	CAV1	1
Tumor Morphology	colony formation	colony formation of small cell lung cancer cells	2.77E-02	CAV1	1
Tumor Morphology	invasion	invasion of nasopharyngeal carcinoma cells	2.77E-02	GNA12	1
Tumor Morphology	quantity	quantity of carcinoma cells	2.77E-02	FOXO3	1
Tumor Morphology	stimulation	stimulation of chronic lymphocytic leukemia cells	2.77E-02	TXLNA	1
Tumor Morphology	proliferation	proliferation of lung cancer cells	3.57E-02	CAV1, SKP2	2
Renal and Urological System Development and Function	binding	binding of kidney cell lines	2.83E-02	CAV1, SDC4, SORT1, ST6GAL1	4
Hair and Skin Development and Function	binding	binding of epithelial cell lines	3.77E-02	CAV1, SDC4, SORT1	3
Hepatic System Development and Function	proliferation	proliferation of hepatocytes	4.20E-02	SKP2, TGFA	2
