# Supplementary material for: microRNA-7-5p inhibits melanoma cell proliferation and metastasis by suppressing RelA/NF-κB
Source: Oncotarget. 2016 May 17;7(22):31663–80. doi: 10.18632/oncotarget.9421 (PMC5077967; doi:10.18632/oncotarget.9421)
Supplement: Supplementary file 6 [file oncotarget-07-31663-s006.pdf]

Supplementary Table S5: Literature review for putative miR-7-5p target genes.

Final List	Fold Change	Comments	References
ACSL4	-1.81	ACSL4 is increased in breast cancer, colon and hepatocellular carcinoma and high expression is associated with an aggressive phenotype. ACSL4, LOX and COX-2 induction and action promote proliferation, migration and invasion of cancer cells.	P.M. Maloberti, A.B. Duarte, U.D. Orlando, M.E. Pasqualini, Á.R. Solano, C. López-Otín, E.J. Podestá, Functional Interaction between Acyl-CoA Synthetase 4, Lipooxygenases and Cyclooxygenase-2 in the Aggressive Phenotype of Breast Cancer Cells, PLoS ONE 5 (2010) e15540.
CAV1	-2.063	CAV1 expression promotes proliferation, whereas it suppresses migration and invasion of B16F10 cells in vitro. CAV1 expression does not affect primary orthotopic tumour growth, but does suppress the ability of B16F10 cells to form lung metastases in C57Bl/6 syngeneic mice. Additionally, CAV1 protein and mRNA levels are found to be significantly reduced in human metastatic melanoma cell lines and human tissue from metastatic lesions.	Trimmer C, Whitaker-Menezes D, Bonuccelli G, Milliman JN, Daumer KM, Aplin AE, et al. CAV1 Inhibits Metastatic Potential in Melanomas through Suppression of the Integrin/Src/FAK Signaling Pathway. Cancer Research. 2010;70:7489-99.
CLIC4	-1.553	CLIC4 expression is generally reduced in malignant tissue compared to normal and overexpression in malignant cells inhibits tumour growth in vivo.	K.S. Suh, M. Malik, A. Shukla, S.H. Yuspa, CLIC4, skin homeostasis and cutaneous cancer: Surprising connections, Molecular Carcinogenesis 46 (2007) 599-604.
CTSK	-6.274	CTSK is expressed in primary melanomas and strongly in metastatic lesions. Inhibition of CTSK reduced invasion of melanoma cells. May play important role in melanoma invasion and metastasis by mediating intracellular degradation of matrix proteins after phagocytosis.	M.J. Quintanilla-Dieck, K. Codriansky, M. Keady, J. Bhawan, T.M. Runger, Cathepsin K in Melanoma Invasion, J Invest Dermatol 128 (2008) 2281-2288.
DPYSL2	-1.62	DPYSL2 (CRMP2) positive rate is significantly increased in earlier stage CRC tumours and lymph node metastasis compared to normal samples of tissue and plasma levels.	C.-C. Wu, H.-C. Chen, S.-J. Chen, H.-P. Liu, Y.-Y. Hsieh, C.-J. Yu, R. Tang, L.-L. Hsieh, J.-S. Yu, Y.-S. Chang, Identification of collapsin response mediator protein-2 as a potential marker of colorectal carcinoma by comparative analysis of cancer cell secretomes, PROTEOMICS 8 (2008) 316-332.
IRS2	-1.509	IRS-2 deficiency (RNAi) inhibits melanoma cell migration and less profoundly invasion.	K.M. Giles, R.A.M. Brown, M.R. Epis, F.C. Kalinowski, P.J. Leedman, miRNA-7-5p inhibits melanoma cell migration and invasion, Biochemical and Biophysical Research Communications 430 (2013) 706-710.
PAK1	-1.631	Increased expression of PAK1 is associated with increased invasive potential of uveal melanoma cells. RNAi of PAK1 reduces invasion of uveal melanoma cells in vitro.	S. Pavey, W. Zuidervaart, F. van Nieuwpoort, L. Packer, M. Jager, N. Gruis, N. Hayward, Increased p21-activated kinase-1 expression is associated with invasive potential in uveal melanoma, Melanoma Res 16 (2006) 285-296.

PAK2	-2.036	Knockdown of Pak1 and Pak2 (RNAi) in ovarian cancer cell lines reduced cell migration and invasion but did not affect cell proliferation and apoptosis.	M.K.Y. Siu, E.S.Y. Wong, H.Y. Chan, D.S.H. Kong, N.W.S. Woo, K.F. Tam, H.Y.S. Ngan, Q.K.Y. Chan, D.C.W. Chan, K.Y.K. Chan, A.N.Y. Cheung, Differential expression and phosphorylation of Pak1 and Pak2 in ovarian cancer: effects on prognosis and cell invasion, International Journal of Cancer 127 (2010) 21-31.
PLXNA1	-1.94	PLXNA1 and its ligand Sema6D are expressed and active in mesothelioma cells and inhibition of PLXNA1 perturbs survival and anchorage-independent growth of mesothelioma cells in a VEGF-R2–dependent manner	A. Catalano, R. Lazzarini, S. Di Nuzzo, S. Orciari, A. Procopio, The Plexin-A1 Receptor Activates Vascular Endothelial Growth Factor-Receptor 2 and Nuclear Factor-κB to Mediate Survival and Anchorage-Independent Growth of Malignant Mesothelioma Cells, Cancer Research 69 (2009) 1485-1493.
POLE4	-6.861	POLE4 is highly expressed in melanoma from patients who relapse compared to those who do not relapse.	A. Kauffmann, F. Rosselli, V. Lazar, V. Winnepenninckx, A. Mansuet-Lupo, P. Dessen, J.J. van den Oord, A. Spatz, A. Sarasin, High expression of DNA repair pathways is associated with metastasis in melanoma patients, Oncogene 27 (2007) 565-573.
RAF1	-2.594	Inhibition of mutant BRAF can cause activation of RAF1 which results reactivates the ERK signaling pathway, promoting drug resistance and melanoma progression.	C. Montagut, S. Sharma, T. Shioda, U. McDermott, M. Ulman, L. Ulkus, D. Dias-Santagata, H. Stubbs, D. Lee, A. Singh, L. Drew, D. Haber, J. Settleman, Elevated CRAF as a potential mechanism of acquired resistance to BRAF inhibition in melanoma, Cancer Res 68 (2008) 4853-4861.
RELA	-1.588	RelA expression is higher in metastatic melanoma cells compared to normal melanocytes and NFkB/RelA dimer has been shown to be constitutively active in metastatic melanoma and inhibition via stable expression of dominant-negative inhibitors of NF-κB activation reduced the formation of lung metastases in mice.	S.E. McNulty, N.B. Tohidian, F.L. Meyskens, RelA, p50 and Inhibitor of kappa B alpha are Elevated in Human Metastatic Melanoma Cells and Respond Aberrantly to Ultraviolet Light B, Pigment Cell Research 14 (2001) 456-465.; S. Huang, A. DeGuzman, C.D. Bucana, I.J. Fidler, Nuclear Factor-κB Activity Correlates with Growth, Angiogenesis, and Metastasis of Human Melanoma Cells in Nude Mice, Clinical Cancer Research 6 (2000) 2573-2581.
RNF144A	-2.581	siRNA-mediated knockdown of endogenous RNF144A accelerates the motility and invasion of breast cancer cells.	H. Marzook, D.-Q. Li, V.S. Nair, P. Mudvari, S.D.N. Reddy, S.B. Pakala, T.R. Santhoshkumar, M.R. Pillai, R. Kumar, Metastasis-associated Protein 1 Drives Tumor Cell Migration and Invasion through Transcriptional Repression of RING Finger Protein 144A, Journal of Biological Chemistry 287 (2012) 5615-5626.
SMO	-1.533	SMO is a membrane receptor integral to the hedgehog signaling pathway (important in melanoma). When activated is acts as an oncoprotein promoting migration, invasion and metastasis of cancer cells.	M.F. Bijlsma, K.S. Borensztajn, H. Roelink, M.P. Peppelenbosch, C.A. Spek, Sonic hedgehog induces transcription-independent cytoskeletal rearrangement and migration regulated by arachidonate metabolites, Cellular Signalling 19 (2007) 2596-2604.

SP1	-1.566	SP1 is transciption factor that binds to the promoter region of MCAM leading to leading to constitutive production of MCAM RNA, which is highly correlated with melanoma cell metastatic potential.	S. Karlen, L.R. Braathen, Regulation of the Melanoma Cell Adhesion Molecule Gene in Melanoma: Modulation of mRNA Synthesis by Cyclic Adenosine Monophosphate, Phorbol Ester, and Stem Cell Factor//c-Kit Signaling, 113 (1999) 711-719.
STMN3	-1.928	STMN3 is expressed in 30% of melanomas and has been found to highly expressed in ovarian metastatic tissue compared to nonmetastatic ovarian tissue.	J. Walter-Yohrling, X. Cao, M. Callahan, W. Weber, S. Morgenbesser, S.L. Madden, C. Wang, B.A. Teicher, Identification of Genes Expressed in Malignant Cells That Promote Invasion, Cancer Research 63 (2003) 8939-8947.
TCF12	-1.841	TCF12 protein functions as transcriptional repressor of E-cadherin, and its overexpression is correlated with metastasis of colorectal cancer.	C.-C. Lee, W.-S. Chen, C.-C. Chen, L.-L. Chen, Y.-S. Lin, C.-S. Fan, T.-S. Huang, TCF12 Protein Functions as Transcriptional Repressor of E-cadherin, and Its Overexpression Is Correlated with Metastasis of Colorectal Cancer, Journal of Biological Chemistry 287 (2012) 2798-2809.
TGFA	-1.553	TGFA is detected in melanoma cells but not melanocytes. Its expression is induced in melanocytes following exposure to UVR and it is associated with metastatic progression in colon cancer models.	Ellem KA, Cullunan M, Baumann KC, Dunstan A. UVR induction of TGF alpha: a possible autocrine mechanism for the epidermal melanocytic response and for promotion of epidermal carcinogenesis. Carcinogenesis. 1988;9:797-801.
