# Supplementary material for: microRNA-7-5p inhibits melanoma cell proliferation and metastasis by suppressing RelA/NF-κB
Source: Oncotarget. 2016 May 17;7(22):31663–80. doi: 10.18632/oncotarget.9421 (PMC5077967; doi:10.18632/oncotarget.9421)
Supplement: Supplementary file 7 [file oncotarget-07-31663-s007.pdf]

Supplementary Table S6: Correlations of miR-7 isoforms with expression of the putative miR-7-5p target genes in the TCGA melanoma cohort.

Genes	Correlation miR-7-1	p-value miR-7-1	Correlation miR-7-2	p-value miR-7-2	Correlation miR-7-3	p-value miR-7-3
RELA	-0.12	0.013	-0.08	0.091	-0.09	0.068
IRS2	-0.22	$4x10^{-6}$	0.02	0.720	-0.10	0.039
POLE4	0.15	0.001	-0.03	0.591	0.09	0.059
PAK1	0.15	0.002	0.01	0.863	0.05	0.258
STMN3	-0.09	0.054	0.01	0.776	-0.02	0.672
SP1	0.04	0.453	-0.08	0.084	-0.09	0.061
TGFA	-0.03	0.559	0.02	0.696	0.02	0.628
RAF1	0.03	0.492	0.01	0.846	0.03	0.488
CTSK	-0.08	0.084	-0.04	0.431	-0.02	0.618
SMO	-0.13	0.006	0.11	0.017	0.04	0.408

Correlates were calculated using Pearson's correlation coefficient. n=452.
