# Supplementary material for: microRNA-7-5p inhibits melanoma cell proliferation and metastasis by suppressing RelA/NF-κB
Source: Oncotarget. 2016 May 17;7(22):31663–80. doi: 10.18632/oncotarget.9421 (PMC5077967; doi:10.18632/oncotarget.9421)
Supplement: Supplementary file 8 [file oncotarget-07-31663-s008.pdf]

Supplementary Table S7: Genes up or downregulated in response to miR-7-5p overexpression in PCR profiler array.

	PCR Array	WM266-4 RT-qPCR			1205Lu RT-qPCR		
**Gene**	**FC**	**FC**	**95% CI**	**p-value**	**FC**	**95% CI**	**p-value**
TNFSF10	-56.2801						
**BCL2A1**	-7.4361	-1.515	-1.616 to -1.413	<0.0005	1.169	0.903 to 1.436	0.2275
TLR3	-6.1669						
**ELK1**	-5.4436	-4.680	-4.996 to -4.364	<0.0005	-2.328	-2.837 to -1.819	<0.0005
PSIP1	-3.9849						
**IL1R1**	-3.7181	-1.971	-2.205 to -1.737	<0.0005	-1.568	-2.009 to -1.127	0.03
**CASP1**	-3.2819	-4.244	-5.302 to -3.186	<0.0005	-2.916	-4.373 to -1.459	<0.0005
**CXCL2**	-3.0410	-4.660	-6.583 to -2.736	<0.0005	-1.363	-1.595 to -1.131	0.009
**BIRC3**	-3.0200	-2.437	-3.487 to -1.386	<0.0005	-1.591	-2.093 to -1.089	0.009
**CSF1**	-2.8571	-5.262	-7.108 to -3.417	<0.0005	-3.165	-4.318 to -2.011	<0.0005
**RELA**	-2.6474	-2.739	-3.516 to -1.963	<0.0005	-2.814	-3.557 to -2.070	<0.0005
**IL1B**	-2.6291	-2.128	-2.853 to -1.404	<0.0005	-2.659	-4.418 to -0.901	0.001
MALT1	-2.4192						
**RELB**	-2.3859	-2.352	-2.730 to -1.974	<0.0005	-2.381	-2.910 to -1.852	<0.0005
**REL**	-2.3368	-1.426	-1.588 to -1.265	<0.0005	-1.457	-1.806 to -1.107	0.012
**IL1A**	-2.0485	-3.091	-3.706 to -2.475	<0.0005	-1.547	-1.895 to -1.198	<0.0005
**IL-6**	-	-3.861	-6.107 to -1.615	<0.0005	-2.300	-3.299 to -1.301	0.0015
**IL-8**	1.3807	-2.039	-2.488 to -1.591	<0.0005	-2.907	-4.721 to -1.092	<0.0005

Bold genes further validated by RT-qPCR.
