# Supplementary material for: microRNA-7-5p inhibits melanoma cell proliferation and metastasis by suppressing RelA/NF-κB
Source: Oncotarget. 2016 May 17;7(22):31663–80. doi: 10.18632/oncotarget.9421 (PMC5077967; doi:10.18632/oncotarget.9421)
Supplement: Supplementary file 9 [file oncotarget-07-31663-s009.pdf]

Supplementary Table S8: Sequences of primers used for RT-qPCR

Gene	Sequence	PrimerBank ID
RELA	Forward: 5'- CCC ACG AGC TTG TAG GAA AGG -3'<br>Reverse: 5'- GGA TTC CCA GGT TCT GGA AAC -3'	223468680c2
CTSK	Forward: 5’- ACT CAA AGT ACC CCT GTC TCA T -3’<br>Reverse: 5’- CCA CAG AGC TAA AAG CCC AAC -3’	315075295c2
POLE4	Forward: 5'- AGA GGG GTG AAC TGA AGG CT -3'<br>Reverse: 5'- TGG ATG AAT TTG CTT TTC TGG -3'	N/A
RAF1	Forward: 5'- CCC AGC ACT ACC TTC TTT GAC -3'<br>Reverse: 5'- AGG GAA CCT TCA GAT GAG GGA -3'	N/A
SMO	Forward: 5'- TCG AAT CGC TAC CCT GCT G -3'<br>Reverse: 5'- CAA GCC TCA TGG TGC CAT CT -3'	300116182c2
STMN3	Forward: 5'- CCA GCA CCA TTT CCG CCT A -3'<br>Reverse: 5'- TGC GGC TGT GTG TAG AAG C -3'	14670374c1
TGFA	Forward: 5'- GGC CCT GGC TGT CCT TAT C -3'<br>Reverse: 5'- AGC AAG CGG TTC TTC CCT TC -3'	N/A
SP1	Forward: 5'- AGT TCC AGA CCG TTG ATG GG -3'<br>Reverse: 5'- GTT TGC ACC TGG TAT GAT CTG T -3'	352962148c2
PAK1	Forward: 5'- AGG GGA GTT TAC GGG AAT GC -3'<br>Reverse: 5'- TCT TCT GCT CCG ACT TAG TGA TA -3'	190886455c2
BCL2A1	Forward: 5'- TAC AGG CTG GCT CAG GAC TAT -3'<br>Reverse: 5'- CGC AAC ATT TTG TAG CAC TCT G -3'	168480070c1
ELK1	Forward: 5'- TCC TAC GCA TAC ATT GAC CC -3'<br>Reverse: 5'- ACT GGA TGG AAA CTG GAA GG -3'	N/A
IL1A	Forward: 5'- AGA TGC CTG AGA TAC CCA AAA CC -3'<br>Reverse: 5'- CCA AGC ACA CCC AGT AGT CT -3'	27894329c2
IL1B	Forward: 5'- ATG ATG GCT TAT TAC AGT GGC AA -3'<br>Reverse: 5'- GTC GGA GAT TCG TAG CTG GA -3'	27894305c1
IL1R1	Forward: 5'- GGC TGA AAA GCA TAG AGG GAA C -3'<br>Reverse: 5'- CTG GGC TCA CAA TCA CAG G -3'	27894331c2
CASP1	Forward: 5'- GCC TGC CGT GGT GAT AAT GT -3'<br>Reverse: 5'- CCT CCA CAT CAC AGG AAC AGG -3'	N/A
CXCL2	Forward: 5'- TCA CAG TGT GTG GTC AAC ATT TC -3'<br>Reverse: 5'- TCT CTG CTC TAA CAC AGA GGG A -3'	N/A
BIRC3	Forward: 5'- TTT CCG TGG CTC TTA TTC AAA CT -3'<br>Reverse: 5'- GCA CAG TGG TAG GAA CTT CTC AT -3'	342307084c2
CSF1	Forward: 5'- TGG CGA GCA GGA GTA TCA C -3'<br>Reverse: 5'- AGG TCT CCA TCT GAC TGT CAA T -3'	166235149c1
RELB	Forward: 5'- CCA TTG AGC GGA AGA TTC AAC T -3'<br>Reverse: 5'- CTG CTG GTC CCG ATA TGA GG -3'	317595683c2
REL	Forward: 5'- CAA CCG AAC ATA CCC TTC TAT CC -3'<br>Reverse: 5'- TCT GCT TCA TAG TAG CCG TCT -3'	56550118c2
ICAM1	Forward: 5'- ATG CCC AGA CAT CTG TGT CC -3'<br>Reverse: 5'- GGG GTC TCT ATG CCC AAC AA -3'	167466197c1
HPRT1	Forward: 5'- ACG AGC CCT CAG GCG AAC CT -3'<br>Reverse: 5'- AAT CAC GAC GCC AGG GCT GC -3'	N/A

GAPDH	Forward: 5'- GGG GTC ATT GAT GGC AAC AAT A -3'<br>Reverse: 5'- ATG GGG AAG GTG AAG GTC G -3'	N/A
